# Oxygen Reduction to Hydrogen Peroxide on Hydrophilic Carbon Fiber Paper: Dependence of the Mechanism and Active Site Stability on Electrolyte pH and Potassium Ion Concentration

**DOI:** 10.1021/acscatal.5c08719

**Published:** 2026-03-26

**Authors:** Connor P. Cox, Madeleine K. Wilsey, Kendra R. Watson, Teona Taseska, Yiwen Sun, Lydia R. Schultz, Samira Siahrostami, Astrid M. Müller

**Affiliations:** † Materials Science Program, 6927 University of Rochester, Rochester, New York 14627, United States; ‡ Department of Chemical and Sustainability Engineering, 6927 University of Rochester, Rochester, New York 14627, United States; § Department of Chemistry, 1763 Simon Fraser University, Burnaby, British Columbia V5A 1S6, Canada

**Keywords:** oxygen reduction reaction, hydrogen peroxide electrosynthesis, hydrophilic carbon fiber paper, binder-free catalyst design, catalyst microenvironment, pH-dependent electrocatalysis, cation effects, computational electrocatalysis

## Abstract

Electrocatalytic H_2_O_2_ synthesis enables decentralized production and reduces reliance on energy-intensive large-scale infrastructure. Practical application, however, requires catalyst materials that are affordable, scalable, and durable. Here, we show that oxygenated carbon fiber paper, hydrophilized through a rapid mild chemistry process developed in-house, serves as an efficient electrocatalyst for the oxygen reduction reaction (ORR) to H_2_O_2_. This catalyst achieves (95 ± 4)% faradaic efficiency and long-term stability for more than 31 h in a divided cell and 100 h in an undivided cell, significantly surpassing traditional particulate carbon catalysts while eliminating the need for supporting electrodes or binders. The analysis of onset potentials versus the reversible hydrogen electrode reveals pH dependence, indicating a nonproton-coupled electron transfer mechanism. When referenced to the standard hydrogen electrode, the onset potentials further suggest that the rate-determining step of the ORR is proton-dependent. Mechanistic studies highlight the coupled roles of oxygenated carbon sites, electrolyte pH, and spectator potassium ions in steering ORR pathways and show that binder-free catalysts are essential for probing the true reaction environment. Higher H_2_O_2_ production rates are obtained at elevated pH, attributed to the greater stability of oxygenated active sites, as confirmed experimentally and supported by density functional theory (DFT) calculations. Hydrophilic carbon fiber paper thus emerges as a robust and viable platform for H_2_O_2_ electrosynthesis. These results also provide mechanistic insight into how oxygen functional groups, electrolyte pH, and potassium cations govern activity and selectivity in ORR.

## Graphical abstract

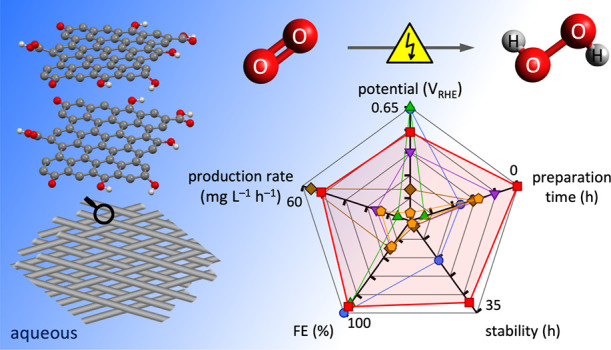

## Introduction

Sustainable chemical manufacturing is central to reducing greenhouse gas emissions and mitigating climate change. Hydrogen peroxide (H_2_O_2_), a vital compound with applications in water purification, bleaching, chemical synthesis,^1^ and semiconductor cleaning,^2^ also plays a critical role in environmental remediation, including the destruction of PFAS pollutants.^3^ Traditionally produced by the energy-intensive anthraquinone process,^4^ H_2_O_2_ production is shifting toward decentralized methods.^5^ Electrocatalysis, particularly when powered by renewable energy, offers a sustainable and cost-effective alternative to large-scale chemical manufacturing.^6^

Electrochemical H_2_O_2_ production relies on the selective 2-electron oxygen reduction reaction (2e-ORR) while suppressing the competing 4e-ORR to water and further reduction of H_2_O_2_. This is schematically illustrated in Figure 1a where structures were geometry-optimized using Avogadro^7^ for visualization, and standard redox potentials for the 2e-ORR to H_2_O_2_ and the 4e-ORR to water at pH 0 and 14 are shown, taken from standard potential tables.^8,9^ Selectivity for H_2_O_2_ arises when the activation barrier for the 4e-ORR exceeds that of the 2e-ORR, enabling kinetic control toward H_2_O_2_.^1^ The overall efficiency depends on both the ORR mechanism and mass transport of O_2_ and H_2_O_2_. Slow mass transport promotes further H_2_O_2_ reduction to water or hydroxyl radicals,^10^ decreasing faradaic efficiency.^11–13^ Although rotating ring-disk electrochemistry (RRDE) optimizes mass transport in laboratory studies, it is not scalable and therefore practical devices typically rely on H-cells or membrane electrode assemblies.^5,14–16^

**Figure 1:**
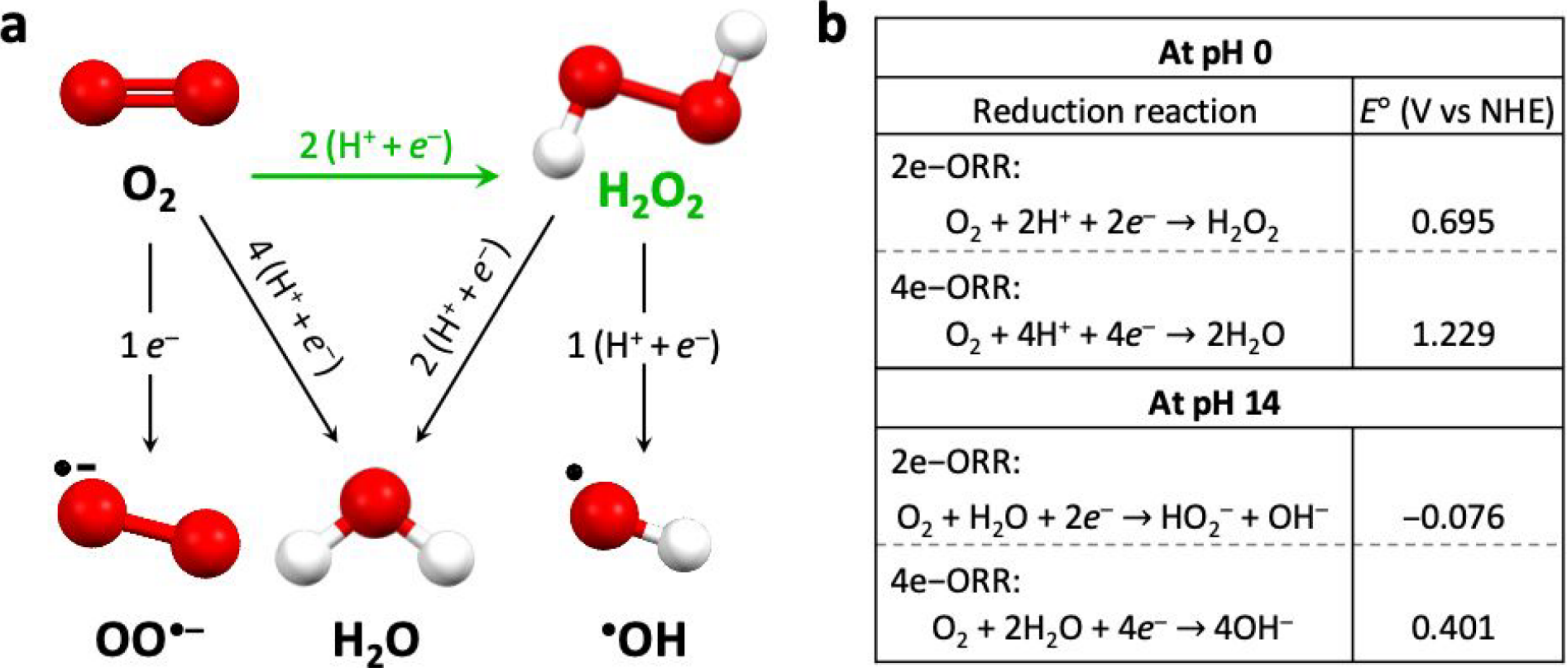
Schematic of aqueous reduction reactions of oxygen and hydrogen peroxide. (a) Aqueous ORR and H_2_O_2_ reduction reactions, with the desired 2e-ORR to H_2_O_2_ highlighted in green. Molecular structures were geometry optimized using Avogadro.^7^ Color code; O: red, H: white. (b) Standard redox potentials of the 2e-ORR to H_2_O_2_ and 4e-ORR to water at pH 0 and 14, taken from standard potential tables; NHE, normal hydrogen electrode.^8,9^

Previous studies employed particulate catalysts that required supporting electrodes and binders for adhesion to conducting supports or deduced mechanistic insights at overpotentials beyond ORR onset. These studies indicated that the ORR mechanism for H_2_O_2_ electrosynthesis depends strongly on electrolyte pH. Below pH 11.6, the protonated pathway dominates: oxygen adsorbs at the catalyst surface, undergoes one-electron reduction to a hydroperoxide intermediate, and either accepts a second electron to form H_2_O_2_ or dissociates to water.^5^ Above pH 11.6, H_2_O_2_ deprotonates and the alkaline mechanism is activated: oxygen undergoes one-electron reduction to a superoxo species,^17–19^ which forms a hydroperoxide intermediate and then deprotonated H_2_O_2_, subsequently reducible to hydroxide ions.^17–19^ H_2_O_2_ decomposition is also more likely in base (pH > 9),^20^ especially in the presence of trace transition metals such as Cu or Fe.^21^ In electrolyzer applications, acidic H_2_O_2_ electrosynthesis is often preferred due to the maturity of proton-exchange-membrane systems, which provide high stability and ionic conductivity, making them economically attractive.^22^ Nevertheless, while both acidic and alkaline conditions have been studied extensively, the highest production rates and faradaic efficiencies are consistently observed in alkaline media.^20,23–28^

Platinum-group metals and their amalgams are highly efficient catalysts for ORR to H_2_O_2_ in acidic electrolytes.^25,29–31^ However, the scarcity and high cost of noble metals,^32–34^ together with the toxicity of mercury, have driven research toward nontoxic, nonprecious alternatives such as carbon-based catalysts.^25,35,36^ Examples include few-layered reduced graphene oxide nanosheets,^26^ graphitic nanoplatelets,^27^ thermally activated carbon fibers,^37^ anodized graphite felt,^38^ and reduced graphene oxide synthetic fabric.^39^ Despite these advantages, most carbon materials exhibit poor ORR activity under acidic conditions.^25^ In alkaline electrolytes, by contrast, carbon materials enriched in surface oxygenates (e.g., COOH, COC, CO–COC) show enhanced activity and selectivity for H_2_O_2_ production.^24^ Oxidized multiwalled carbon nanotubes, reduced graphene oxide, and modified carbon black are prominent examples, achieving faradaic efficiencies of 90–100% through treatments such as oxidation, reduction, or plasma processing.^26^

Oxygenated carbon-based materials have been reported as effective 2e-ORR catalysts.^23^ Their major limitation, however, is that as particulate catalysts they require electrode supports and binders to maintain electrical contact, which reduces durability and energy efficiency and complicates mechanistic analysis by introducing extrinsic species into the catalyst microenvironment.^40–42^ The synthesis routes for particulate catalysts are often tedious, complex, and costly.^40^ Adhesion to electrode supports typically involves binders that alter the chemistry at the electrode–electrolyte interface.^43^ For accurate mechanistic studies across a wide pH range, binder-free conditions are essential to avoid uncontrolled changes in the catalyst microenvironment.^44–46^ Binder-free designs also reduce materials waste, enhance catalyst stability,^40,41^ and enable direct comparison of catalysts with theory unencumbered by binder chemicals,^47^ which is critical for establishing interfacial processes without interference from extrinsic species that complicate reaction mechanisms and give rise to competing pathways.^48–50^

In this study, we employed our in-house developed hydrophilic carbon fiber paper cathodes, featuring surface oxygen functional groups, as a nonprecious, effective ORR to H_2_O_2_ catalyst. We investigated this catalyst across seven aqueous electrolytes (pH 0.6–14) to identify the mechanistic descriptors governing activity and selectivity. The carbon catalyst is inexpensive, scalable, nontoxic, and electrically conductive, with surface oxygenates generated through a rapid, acid-free, green chemistry process^51^ that renders it hydrophilic and suitable for aqueous H_2_O_2_ electrosynthesis. Unlike particulate catalysts, it requires no supporting electrodes or binders, enabling precise control of interfacial microenvironments. A distinct feature of our hydrophilization process is that it preserves the inherent high porosity of 78% in carbon fiber paper,^52^ ensuring that mass transport in electrode processes is not impeded.^40^ After hydrophilization, we determined a 468-fold enhancement in surface area relative to the geometric area,^52^ corresponding to a total carbon surface area of 3767 cm^2^ for the catalytic cathode used here, which had geometric dimensions of 3.5 cm × 2.3 cm. Importantly, hydrophilization also promotes water attraction to the carbon surface,^53^ which is essential for ORR because water participates directly in turnover in base (Figure 1b).^1^

In electrocatalysis, potentials are commonly reported versus the standard hydrogen electrode (SHE), the normal hydrogen electrode (NHE), or the reversible hydrogen electrode (RHE), and careful distinction among these reference scales is essential for accurate comparison of kinetic and thermodynamic data. The SHE and NHE are closely related fixed reference scales, both defined for the H^+^/H_2_ redox couple. The SHE is defined at unit proton activity under standard-state conditions and corresponds to a hypothetical, ideal reference electrode. In contrast, the NHE is defined at unit proton concentration, which does not represent a true electrochemical standard state. Historically, the NHE was treated as an experimental realization of the SHE; however, the difference in potential between the NHE and the SHE is non-negligible because finite concentration and ideal behavior cannot be simultaneously satisfied.^54^ In contrast to the NHE, the RHE is a pH-dependent reference that explicitly accounts for proton activity in the electrolyte, such that the potential of the H^+^/H_2_ couple is defined as 0 V at all pH values. As a result, conversion between SHE and RHE requires explicit pH correction via the Nernst equation, according to *E*_SHE_ = *E*_RHE_ + 0.059 · pH at 298.15 K.^9,55,56^ Reporting potentials versus RHE is therefore particularly advantageous for proton-coupled electron transfer reactions, including aqueous ORR,^1,57–59^ as it enables direct comparison of catalytic activity across electrolytes of different pH while maintaining a consistent thermodynamic reference. Therefore, all potentials in this work were measured and are reported versus RHE.

By measuring H_2_O_2_ production rates, current densities, and faradaic efficiencies while systematically varying electrolyte pH and potassium ion concentration, we identify how electrolyte conditions and surface species govern H_2_O_2_ electrosynthesis at hydrophilic carbon fiber paper catalysts. Combined electrochemical performance data, XPS analysis, and DFT calculations disentangle the roles of proton dependence of the rate-limiting step, stability of oxygenated active sites, and potassium ion field and adsorption effects. This integrated approach establishes a mechanistic framework for optimizing selectivity and efficiency of the 2e-ORR pathway and guides the rational design of sustainable carbon-based catalysts for H_2_O_2_ electrosynthesis.

## Results and Discussion

### ORR Catalyst and H_2_O_2_ Electrosynthesis

We employed a rapid, green chemistry process to functionalize initially hydrophobic carbon fiber paper with surface oxygenates, rendering it hydrophilic and enabling adsorption of interfacial water. This environmentally benign treatment is suitable for large electrode areas.^53^ Specifically, commercial carbon fiber paper was first sonicated for 5 min in 1.0 M aqueous sodium dodecyl sulfate solution, then electrooxidized for 20 min in 0.1 M pH 8.7 aqueous KHCO_3_ electrolyte at +1.63 V vs Ag/AgCl.^53^ The complete oxygenation treatment required only 27 min and eliminated the use of harsh acids or transition metals, thereby avoiding associated environmental and safety concerns while ensuring the absence of transition-metal contamination on the carbon surface.^53^

Electrical conductivity of the carbon fiber paper is critical for its use as a macroscopic electrode material.^53,60–69^ To verify that the hydrophilization treatment did not affect conductivity, we collected electrochemical impedance spectroscopy (EIS) data and derived conductivity values before and after hydrophilization. EIS measurements were performed in acetonitrile electrolyte because untreated carbon fiber paper is hydrophobic, and poor wettability in water would introduce a large interfacial resistance that would dominate the impedance response in an aqueous system. Although hydrophilized carbon fiber paper was used in the ORR measurements, comparison with untreated material was necessary because untreated carbon fiber paper is a commonly used electrode substrate.^53,60–69^ EIS data (Supporting Information Figure S1) yielded resistance values of (7.2 ± 0.2) and (7.4 ± 0.2) Ω, for untreated and hydrophilized carbon fiber paper, consistent with literature values.^70^ Conductivity values derived from these resistances were (2.8 ± 0.2) and (2.7 ± 0.2) S m^–1^, respectively, confirming that hydrophilization did not change the carbon fiber paper conductivity within error.

The carbon fiber network was preserved during treatment, maintaining a porous architecture with high internal surface area, as evident from SEM imaging (Figure 2a). At the same time, the process roughened carbon fiber surfaces, creating graphitic edge sites where surface oxygenates are stabilized.^71^ Unlike graphene, basal-plane carbon atoms in graphite are unreactive toward oxygenate functionalization due to π-stacking interactions between adjacent layers,^41^ which render oxygenated species at basal sites thermodynamically unfavorable.^72^ Therefore, oxygenated carbon edges are the predominant reactive sites, although most carbon sites are located at the basal planes. These graphitic edges are clearly visible in SEM images of individual carbon fibers (Figure 2a). Cooperative effects between oxygenated graphitic edges and adsorbed interfacial water have been identified as the basis for the durable hydrophilicity of carbon fiber paper,^53^ which we have verified to persist for more than four years when the material is stored under water.

**Figure 2:**
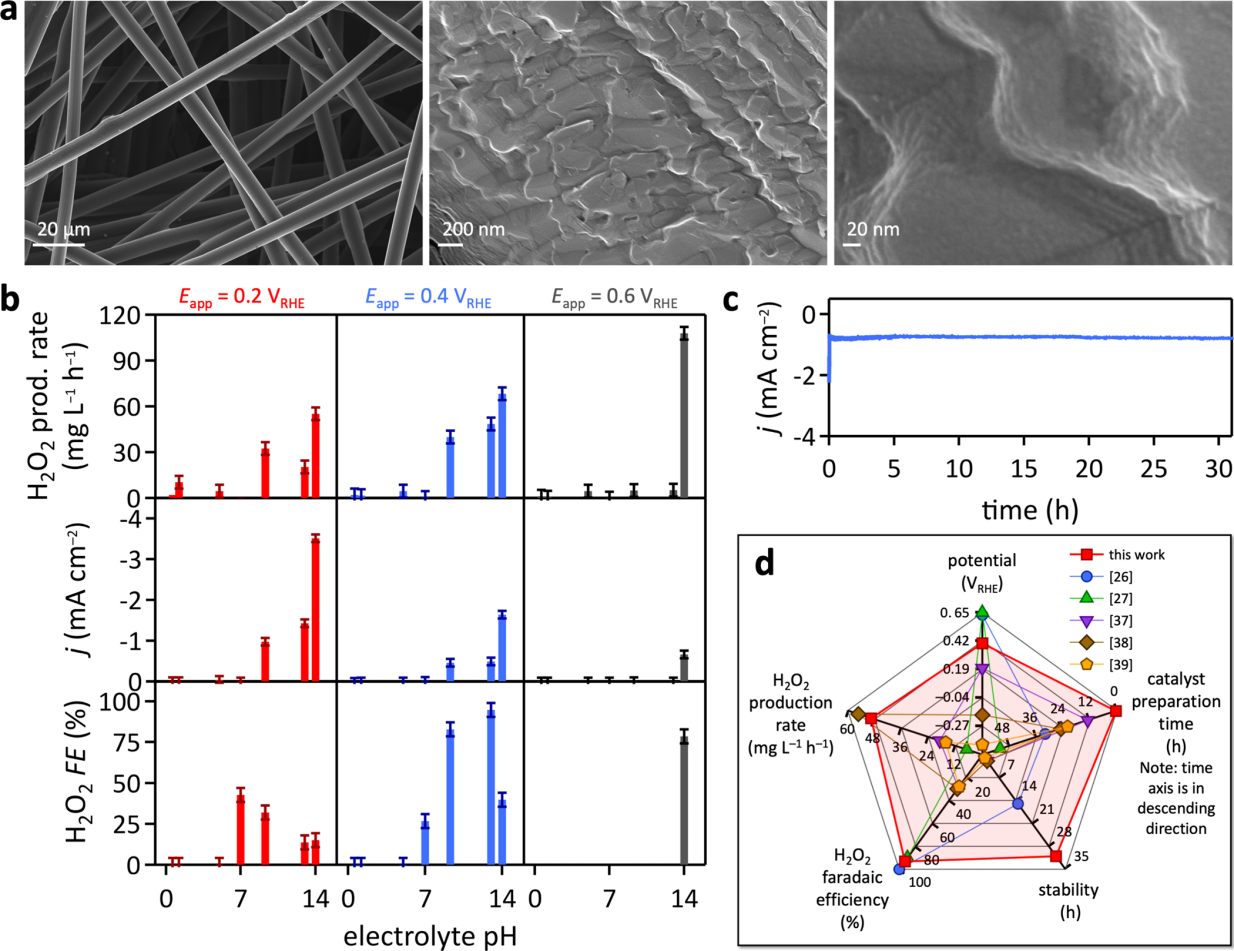
ORR catalyst structure and electrochemical performance. (a) SEM images of hydrophilic carbon fiber paper. (b) H_2_O_2_ production rate, generated current density, and H_2_O_2_ faradaic efficiency­(*FE*) as a function of applied potential and electrolyte pH in O_2_-saturated electrolytes. (c) Long-term stability of hydrophilic carbon fiber paper cathodes in ORR electrocatalysis at pH 13 at an applied potential of 0.4 V vs RHE. (d) Radar chart showing the superior performance of the hydrophilic carbon fiber paper ORR catalyst, compared to reported carbon ORR catalysts.^26,27,37–39^

### H_2_O_2_ Production

The rates of H_2_O_2_ generation at overpotentials of 0.095 V (*E*_app_ = 0.6 V vs RHE), 0.295 (*E*_app_ = 0.4 V vs RHE), or 0.495 (*E*_app_ = 0.2 V vs RHE) were consistently higher at higher pH, in agreement with previous reports on electrosynthesis of H_2_O_2_ on carbon-based materials (Figure 2b). H_2_O_2_ production was observed only in O_2_-saturated electrolytes, whereas no H_2_O_2_ was detected in Ar-saturated electrolytes (Figure S2), as expected for ORR. The highest H_2_O_2_ production rate was achieved in aqueous 1.0 M pH 14.0 KOH electrolyte at 0.6 V vs RHE, the least reducing potential studied, yielding (108 ± 4) mg L^–1^ h^–1^.

The actual H_2_O_2_ production could be even higher, as HO_2_^–^ formed along the high-pH mechanistic pathway can permeate through the alkaline exchange membrane and undergo oxidation in the counter electrode compartment, thereby escaping detection as H_2_O_2_. An alkaline exchange membrane was used instead of Nafion in aqueous 1.0 M pH 14.0 KOH electrolyte, because the Donnan exclusion mechanism required for Nafion to function as a proton exchange membrane fails at such high hydroxide concentrations. Specifically, Nafion 117 has an average sulfonate group concentration of 1.13 M,^73^ which is comparable to the hydroxide concentration in 1 M KOH. Ion exclusion in an ion exchange membrane is only effective if the fixed functional group concentration within the membrane exceeds the electrolyte ion concentration by at least an order of magnitude.^74^

Our hydrophilic carbon fiber paper electrode is a macroscopically porous material with a high surface area of 468 cm^2^ per geometric cm^2^, carbon fiber mean diameter of (6.8 ± 0.6) μm, and high porosity of 78%,^52^ at which axial rotation cannot produce laminar flow. As a result, the fundamentals of RRDE theory^75^ break down and are not applicable to hydrophilic carbon fiber paper electrodes (see Supporting Information Notes section for further discussion). In addition, establishing a stable dry electrical contact to hydrophilic carbon fiber paper remains an unsolved technological challenge. Consequently, RRDE measurements are not possible with the hydrophilic carbon fiber paper electrodes of this work. Nevertheless, hydrophilic carbon fiber paper provides the important advantage of enabling binder-free assessment of electrocatalytic performance.

Our H_2_O_2_ production rate at 0.4 V vs RHE in aqueous 0.1 M pH 13.0 KOH electrolyte is (49 ± 4) mg L^–1^ h^–1^, comparable to reported values for carbon catalysts in liquid H-cells operated under similar potentials (Supporting Information Table S1). Under these conditions, the maximum cumulative molar concentration of H_2_O_2_ was (2.9 ± 0.2) mM. While mass activity is often used to benchmark particulate ORR catalysts,^23,26^ it is not an appropriate metric for hydrophilic carbon fiber paper, a macroscopic, nonparticulate material that functions without a catalyst support. We therefore used the H_2_O_2_ production rate as the figure of merit. Temporal profiles of H_2_O_2_ concentration during ORR (Figures S3–S5) increased continuously and linearly with time, as expected. Although product generation is generally higher in gas diffusion systems than in liquid electrolyzers with gas-phase reactants,^76–79^ as in ORR electrocatalysis, we employed the liquid configuration here to probe the mechanistic origins of the observed pH dependence in electrocatalytic performance.

Trends of H_2_O_2_ production rates as a function of applied potential depend strongly on electrolyte pH, consistent with previous observations.^5,11,17–19,80–85^ In electrolytes with a pH < 9, higher H_2_O_2_ production rates were obtained at more negative applied potentials, whereas in alkaline electrolytes (pH > 9), the trend reversed, with higher H_2_O_2_ production rates observed at less reducing potentials (Figure 2b). Because H_2_O_2_ is prone to decomposition in base, especially at pH > 9,^20^ the measured concentrations may reflect steady-state equilibria between production and decomposition at each applied potential and electrolyte pH. Importantly, spectrophotometric titration detects only bulk H_2_O_2_ concentrations, not transient intermediates.

To suppress H_2_O_2_ decomposition, 400 ppm MgSO_4_ is sometimes added to the electrolyte as a stabilizer.^27^ We did not add MgSO_4_ in this study to avoid further complicating the electrocatalytic reaction network. In addition, aqueous base equilibrated with ambient air contains carbonate,^86^ which can react with Mg^2+^ to form insoluble magnesium (hydroxy)­carbonate solids that could foul the carbon catalyst.

### Selectivity for H_2_O_2_ Production

Current densities were obtained from chronoamperometry measurements in O_2_- and Ar-saturated electrolytes (Figures S6–S8). In Ar-saturated electrolytes, the hydrophilic carbon fiber paper catalyst exhibited current densities below −0.014 mA cm^–2^, confirming that virtually no reduction reactions occurred under anaerobic conditions and that dioxygen was the only reducible species in O_2_-saturated electrolytes (Figure 2b). At a given applied potential, current densities increased with electrolyte pH.

Interestingly, the current density trends are opposite to the H_2_O_2_ production rate trends at individual pH values across the three applied potentials. In alkaline electrolytes (pH > 9), higher current densities are observed at more reducing potentials, whereas H_2_O_2_ production rates decrease under those conditions. In contrast, in electrolytes with pH < 9, both current densities and H_2_O_2_ production rates are small but followed the same trend, with higher values obtained at more reducing applied potentials.

Current density data encompass all redox processes, including the desired 2e-ORR to H_2_O_2_ and the competing 4e-ORR to water. The balance between these pathways depends on electrolyte pH and applied potential, as described above. Additional loss channels include further reduction of generated H_2_O_2_ to water or hydroxyl radicals. The 1e-ORR pathways to radical species (Figure 1a) are not relevant under the conditions of this work because potential-leveling by proton-coupled electron transfer^87^ renders the 2e- and 4e-ORR routes dominant in aqueous electrolytes.^1^

Faradaic efficiencies for H_2_O_2_ electrosynthesis are shown in Figure 2b. At 0.6 V vs RHE in all electrolytes, and at all applied potentials in acidic electrolyte, H_2_O_2_ concentrations are low, leading to small denominators in the faradaic efficiency calculations and correspondingly large uncertainties. The highest faradaic efficiency of (95 ± 4) % for ORR to H_2_O_2_ is obtained at 0.4 V vs RHE in O_2_-saturated aqueous 0.1 M pH 13.0 KOH in the liquid H-cell configuration. This value is comparable to reported faradaic efficiencies in liquid H-cells under similar conditions, as well as in RDE and RRDE experiments, where mass transport is faster than in the H-cell geometry^23,25–28,88–91^ (see Table S1).

### Catalyst Stability

The hydrophilic carbon fiber paper cathodes display exceptional long-term electrochemical stability for ORR electrocatalysis in pH 13.0 aqueous KOH electrolyte at an applied potential of 0.4 V vs RHE, the condition of highest H_2_O_2_ faradaic efficiency. Current generation in the divided ORR cell remained virtually unchanged over the course of 31 h of continuous operation (Figure 2c), representing record long-term stability among reported carbon ORR catalysts operated without stabilizing agents.^26,37–39,88,92–98^ We found that the performance degradation observed after 31 h was caused by the divided cell configuration. The chemical stability of polymer membranes against reactive oxygen species generated during H_2_O_2_ evolution is a known challenge.^99^ Although use of an H-cell is standard practice in investigating H_2_O_2_ electrosynthesis,^1^ the membrane-containing configuration limits stable current generation to 31 h in hydrophilic carbon fiber paper-catalyzed ORR (Figure 2c). In contrast, chronoamperometry measurements performed in a membrane-free undivided cell showed that the hydrophilic carbon fiber paper catalyst remained stable for 100 h, with no detectable changes to the carbon fiber architecture or surface species compared to the state after 2 h (Figure S9). These results demonstrate that the catalyst stability far exceeds the 31 h observed in H-cell experiments.

The carbon fiber mesostructures remain intact after ORR electrocatalysis, as confirmed by SEM imaging (Figure S10), indicating that hydrophilic carbon fiber paper is a stable cathode material under reductive polarization across a wide pH range (0.6–14). Such durability is not attainable by most metal catalysts^100^ nor by particulate carbon catalysts, which suffer from poor adhesion to supports across broad pH ranges and from binder effects that alter the catalyst–electrolyte microenvironment.^44–46^

### Multivariate Performance Analysis

The overall performance of hydrophilic carbon fiber paper ORR catalyst was superior to reported carbon ORR catalysts,^26,27,37–39^ as illustrated in the radar chart (Figure 2d). The figures of merit include long-term stability (without additional stabilizing agents), H_2_O_2_ production rate, H_2_O_2_ faradaic efficiency, applied potential, and preparation time from commercially available chemicals and materials, capturing the complexity of carbon modification processes for practical application.

The catalyst of this work is compared to reported data obtained from ORR electrocatalysis in H-cell and one-compartment cell geometries, and not from gas diffusion electrode, flow, or RRDE cells, because cell geometry critically influences mass transport and therefore performance. The hydrophilic carbon fiber paper catalyst spans the largest area on the radar plot and outperforms reported carbon ORR catalysts, providing a substantial advantage over previously studied carbon materials for H_2_O_2_ electrosynthesis.

### Mechanistic Analysis

Having established the catalytic activity, selectivity, and stability of hydrophilic carbon fiber paper for ORR to H_2_O_2_, we now turn to the mechanistic factors that govern its performance. We focus on how electrolyte pH and applied potential shape the competition between 2e- and 4e-ORR pathways, how interfacial properties of oxygenated carbon sites and potassium ion concentrations influence reactivity, and how these descriptors explain the observed trends in H_2_O_2_ electrosynthesis performance.

### Onset Potential As a Function of Electrolyte pH

We began with analysis of the onset potential as a function of bulk electrolyte pH, because onset potential analysis requires lower applied overpotentials compared to steady-state Tafel data. The onset potential is the potential where electrocatalysis starts, i.e., where all thermodynamic and kinetic barriers are surmounted.^9^ Tafel slopes are linear only at overpotentials greater than approximately 150 mV,^9^ at which the undesired 4e-ORR pathway to water becomes more favorable. As a result, Tafel data cannot be used to isolate the 2e-ORR contribution. Therefore, analysis of onset potentials, determined from nonsteady-state linear sweep voltammetry data, is more appropriate for evaluating H_2_O_2_ electrosynthesis via the 2e-ORR than Tafel analysis.

We emphasize that we deliberately did not derive performance metrics from onset potential data, because only steady-state Tafel data prevent overestimation of catalytic performance due to inevitable charging processes at solid catalysts that occur upon a change of applied potential at early times.^9^ Nevertheless, onset potential analysis reveals mechanistic changes in ORR at hydrophilic carbon fiber paper cathodes as a function of electrolyte pH, which we varied from 0.6 to 14. Onset potentials generally depend on the nature of the catalyst, real surface area, mass transport of reactant and product species, local ion concentrations in the catalyst microenvironment, and the electrocatalytic mechanism.^9^

The onset potentials were obtained as the intersect of tangent lines from rising currents and baseline currents (Figure S11). Our approach follows a widely accepted methodology for determining onset potentials in electrochemistry.^9^ As the same catalyst and stirring conditions were used across all pH values, contributions from changes in catalyst nature, real surface area, and mass transport can be neglected. Thus, only mechanistic factors and electrode microenvironment effects influence the onset potential. Analysis of onset potentials as a function of electrolyte pH is therefore a useful tool for gaining mechanistic insight.

We found that onset potentials vs RHE showed a linear dependence on electrolyte pH with a slope of (0.055 ± 0.009) V per pH unit (Figure 3a). Since the RHE inherently accounts for pH,^9,55,101^ a proton-coupled electron transfer (PCET) reaction involving the same number of protons and electrons transferred should exhibit no shift in *E*_RHE_ as a function of pH. Therefore, the observed dependence of the potential on electrolyte pH suggests that the ORR does not proceed via a PCET mechanism. Non-PCET behavior has been reported for proton reduction on platinum and has been attributed to pH-dependent coadsorption of alkali cations,^102^ changes in reorganization energy,^103^ and shifts of the potential of maximum entropy.^104^

**Figure 3:**
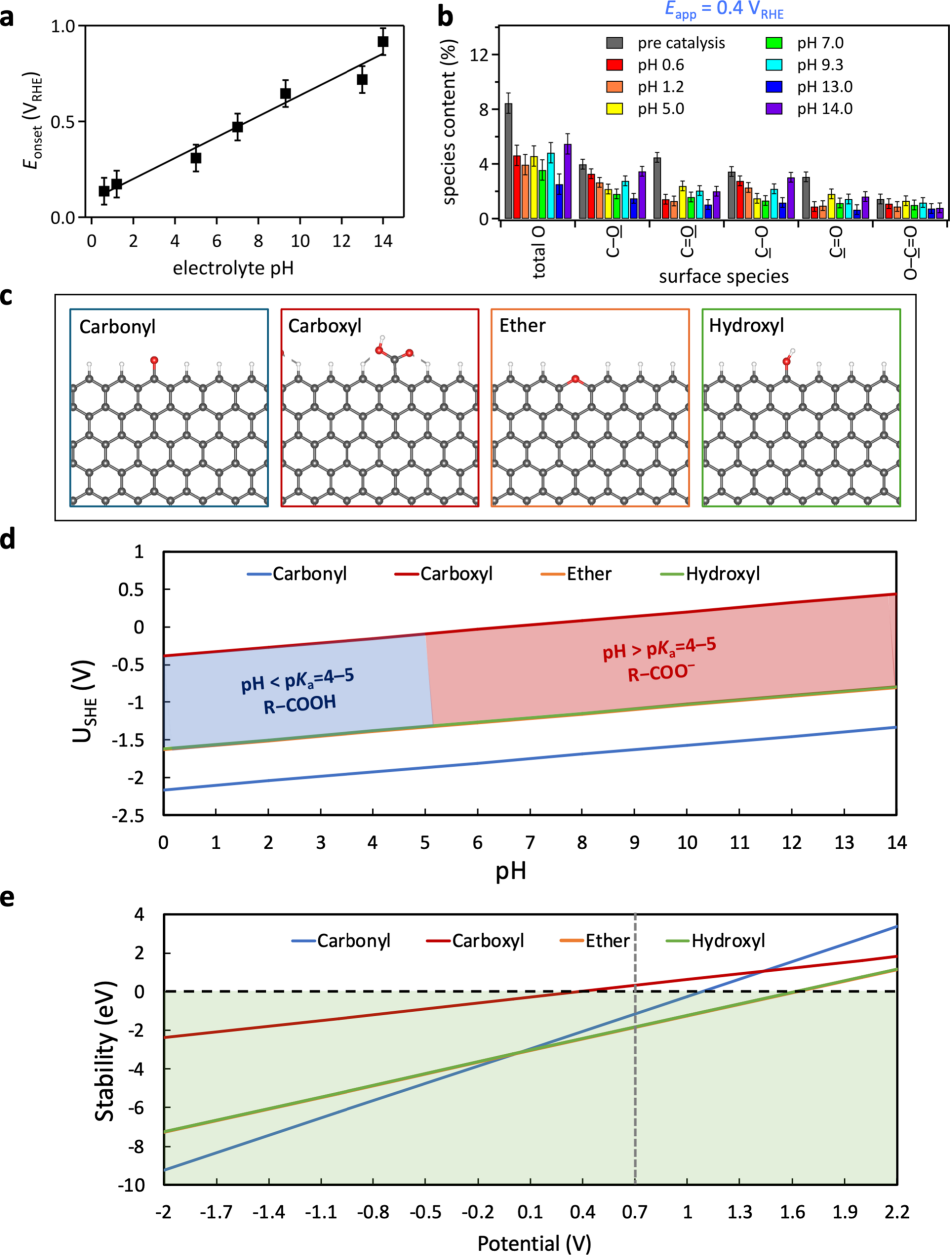
pH dependence of onset potential and carbon surface oxygenate stability. (a) Onset potentials for H_2_O_2_ generation as a function of electrolyte pH, obtained from ORR catalyzed by hydrophilic carbon fiber paper cathodes. Squares, data; line, linear fit. (b) Total carbon surface oxygenate contents derived from XPS data of hydrophilic carbon fiber paper pre catalysis and after 2 h of electrocatalysis in aqueous electrolytes with pH values ranging from 0.6 to 14 at an applied potential *E*_app_ of 0.4 V vs RHE. (c) Model structures for simulating the surface oxygenates. Color code; C: gray, O: red, H: white. (d) Calculated Pourbaix diagram for various surface oxygenates displayed in (c). Blue and red shaded areas mark the regions where carboxylic acids transition between protonated and deprotonated forms as the pH and potential change. (e) Calculated stability vs potential with the region of stability highlighted in green. The gray vertical dashed line marks the standard redox potential for the 2e-ORR (0.7 V), indicating that all oxygenates are stable (with negative formation energy). Because of their similarities in nature, the stability of C–O oxygenates such as hydroxyl and ether overlap.

### Mechanistic Phenomena Underlying the pH-Dependence of Onset Potentials

In principle, the observed pH-dependence of onset potentials on bulk electrolyte pH could arise from several distinct mechanistic contributions, which we evaluate individually to disentangle their roles in governing ORR selectivity and efficiency.

1. Proton-dependence of the rate-determining step (RDS). Observation of more negative ORR onset potentials at acidic pH, as seen here, has been attributed to a noncoupled proton–electron transfer mechanism for 2e-ORR in RDE experiments on nitrogen-doped reduced graphene oxide cathodes.^105,106^ A pH-independent RDS has been reported for precious metals, whereas the RDS involves H^+^ at nonprecious metals.^107^
2. Interfacial proton concentration differing from the bulk. The local proton concentration at the oxygenated carbon fiber paper surface may differ from that in the bulk electrolyte. In this context, the buffering capacity of activated carbon has been implicated in redox reactions.^108,109^ The cathode microenvironment can differ significantly from the bulk electrolyte during electrochemical reactions: changes in reactant, intermediate, and product concentrations, pH gradients, ion accumulation, and depletion of buffering species have all been reported in electrocatalytic CO_2_ reduction and hydrogen evolution reactions.^110–115^ However, it is unlikely that the local pH remains constant across the bulk pH range from 0.6 to 14, since the functional groups capable of buffering are carbon oxygenates, which are expected to have p*K*_a_ values in the range of 4–15. These estimates are based on p*K*_a_ values of phenol (10.0),^116,117^ benzyl alcohol (15.4),^118^ and benzoic acid (4.2),^118^ representing sp^2^ and sp^3^ alcohols and sp^3^ carboxylic acids, because the respective p*K*_a_ values of graphitic carbon oxygenates are unknown.
3. pH-dependent stability of active sites. The stability of active sites could depend on the bulk electrolyte pH value at the respective applied potential. The binder-free design of our catalyst is critical in this mechanistically complex system at the interfacial catalyst microenvironment, where extrinsic molecules fundamentally alter mechanistic pathways and electrocatalytic performance.^42^
4. Field effects of potassium ions. Field effects of alkali metal ions in the electrochemical double layer could influence ORR energetics.^119–121^

5. Potassium ion adsorption. Spectator ions, such as K^+^, may adsorb at ORR active sites in a pH-dependent manner, thereby blocking catalytic sites. For small adsorbates on metal surfaces, however, pH independence vs RHE have been observed.^101^

The five mechanistic phenomena outlined above are not mutually exclusive, and our data allow us to disentangle their relative importance as a function of electrolyte pH, potassium ion concentration, and applied potential. We next identify the governing factors that determine the ORR electrocatalysis of hydrophilic carbon fiber paper cathodes.

### Mechanistic Contribution 1: Proton Dependence of the RDS

To evaluate whether the RDS is proton dependent at the hydrophilic carbon fiber paper catalyst, we plotted onset potentials on the standard hydrogen electrode (SHE) scale, as is common practice to include the thermodynamic dependence on pH.^55,122^ The onset potential vs pH data on the SHE scale are shown in Figure S12. The electrode potential vs RHE is related to the SHE scale by the Nernst equation: *E*_SHE_ = *E*_RHE_ + 0.059 • pH.^9,55,56^ If the RDS is proton-independent, the onset potential should be independent of pH when plotted against the SHE. However, we found that the onset potentials vs SHE depended linearly on the bulk electrolyte pH with a slope of 0.114 ± 0.008 V pH^–1^.

This result indicates that the reaction thermodynamics depends on pH, in contrast to studies on mildly reduced graphene oxide supported on carbon paper, which concluded proton independence of the RDS^106^ due to a sign error introduced during the RHE-to-SHE conversion. As a result, the reported pH trend vs SHE does not reflect the actual thermodynamic behavior, and the mechanistic conclusions from that work are not applicable to our system. Our finding of a slope of 0.114 V vs SHE demonstrates that the RDS is not proton-independent but instead involves PCET, in which proton availability directly modulates reaction kinetics.

Entropically driven changes in activation free energy for proton transfer across the outer Helmholtz layer have also been proposed under alkaline conditions.^103^ However, for ORR at low overpotentials, electrode surface reaction barriers dominate over entropic transport barriers, regardless of pH.^123^ Unlike most metal catalysts^100^ or particulate carbon catalysts, which suffer from poor adhesion to electrode supports and require binders that alter the catalyst–electrolyte interface,^44–46^ the carbon fiber paper catalyst maintains stability and functionality across the entire pH range studied. We surmise that previously observed mechanistic changes^124^ arose from alterations in binder chemistry rather than genuine changes in the ORR mechanism at carbon surfaces. Only binder-free experiments with a nonparticulate solid catalyst material, such as the hydrophilic carbon fiber paper reported here, enable control of the catalyst microenvironment that is prerequisite for a quantitative mechanistic understanding of ORR at carbon surfaces.

### Mechanistic Contribution 2: Interfacial Proton Concentration

To assess whether differences between local and bulk proton concentrations could explain the observed pH-dependence of onset potentials, we examined our performance data and compared our results to the literature. Local proton buffering and potential-driven pH changes at catalyst surfaces have been reported for CO_2_ reduction systems,^125^ but such effects are unlikely to persist consistently across the wide bulk pH range from 0.6 to 14 studied here. The functional groups that could provide buffering are surface carbon oxygenates with estimated p*K*_a_ values between 4 and 15, making it improbable that local proton concentrations remain constant relative to the bulk under all conditions.

Related work on 2e-ORR catalysts consisting of platinum-group-metal-free carbon–nitrogen systems prepared as Nafion inks suggested that the local pH at the cathode equaled the bulk pH of the system.^124^ However, those results are difficult to interpret because of complicating binder effects. Nafion is widely used as a binder for ORR catalysts due to its high proton conductivity and oxygen permeability,^126^ but its own pH dependence^127^ and lack of buffering capacity^128^ can alter the catalyst–electrolyte microenvironment. In our binder-free hydrophilic carbon fiber paper catalyst, these complications are absent, indicating that local proton concentration differences are unlikely to account for the observed onset potential trend. Therefore, this behavior must arise from factors other than the interfacial proton concentration; these factors are discussed in the following sections.

### Mechanistic Contribution 3: pH-Dependent Stability of Active Sites

To evaluate how the stability of active sites depends on electrolyte pH and to gain mechanistic insights, we first identified and quantified carbon oxygenates on the hydrophilic carbon fiber paper catalysts before and after ORR electrocatalysis in electrolytes of different pH values. We then assessed the stability of these carbon surface oxygenates by DFT calculations. Edge oxygen functional groups such as ether and carboxyl have been reported to play an important role in H_2_O_2_ electrosynthesis.^24,25^ Accordingly, surface oxygenates on hydrophilic carbon fiber paper before catalysis and after 2 h of electrocatalysis were quantified by XPS, following ref 53. XPS enables direct detection of surface functional groups because of its shallow probe depth of about 8.7 nm for graphite.^129^ In contrast, Raman and FTIR spectroscopies, though often used to identify oxygen functionalities of bulk materials, lack sufficient surface sensitivity. For hydrophilic carbon paper, the underlying carbon framework dominates the signal from oxygenated surface species,^53^ making XPS the most effective technique for characterizing surface modifications, where catalysis necessarily occurs.

We observed oxygen functional groups in the high-resolution C 1s and O 1s spectra and assigned them using both elemental regions. The corresponding spectra, peak fits, and quantifications are shown in Figures S13 – S32. Surface oxygen contents are reported relative to total surface carbon, consistent with ref 53. The O 1s spectra required two to three peaks to match the experimental data, with central binding energies at 531.6–532.3 eV (C=O functional groups: carboxyls, aldehydes, carbonyls, esters, ketones), 533.0–533.7 eV (C–O species: carboxyls, hydroxyls, ethers),^130–133^ and 535.3–536.0 eV (adsorbed water).^134,135^ Correspondingly, we constrained the central binding energies of the three oxygenate peaks in the C 1s spectra (Figures S13 – S26) to the following ranges: 286.4–287.0 eV (C–O: hydroxyls, esters, ethers), 287.4–288.0 eV (C=O: aldehydes, carbonyls, ketones), and 288.7–289.2 eV (O–C=O: carboxyls, esters).^130–133,136–138^ These multipeak fits matched the measured data well (Figures S13 – S26) and provided quantifications of individual oxygenated carbon species as a function of electrolyte pH and O_2_ or Ar saturation. XPS data analysis showed that electrocatalysis affected the amount of oxygenates on carbon fiber paper (Figure 3b and Figure S33).

Surface O–C=O contents as a function of electrolyte pH, both pre-electrocatalysis and postcatalysis, are shown in Figure 3b and Figure S33. Surface O–C=O species (e.g., COOH, COO) together with COH groups are reported primary active sites for the 2e-ORR to H_2_O_2_.^24^ The XPS data together with the observed trend of ORR onset potentials as a function of pH highlight the need for an alternative mechanistic explanation to account for increased H_2_O_2_ production at higher pH values. Below, we discuss in more detail how electrolyte pH and composition impact the surface oxygenate content using XPS analysis.

Relative to pre-electrocatalysis electrodes, the total surface oxygen content decreased after 2 h of electrocatalysis in all electrolytes and at all applied potentials, regardless of pH (Figures 3b and S33). In aqueous perchloric acid (pH 0.6 and 1.2), total oxygen content was similar, although oxygenates are generally more stable in acidic environments (Figure 3c,d). High-resolution Cl 2p spectra revealed chlorine-containing surface species at a central binding energy of 209 eV (Figures S34 and S35), attributable to perchlorate,^139,140^ indicating that surface perchlorate contributed to the observed C–O signal at low pH. Prior reports have assigned C–Cl and Cl–O species to 286.2 and 532.5 eV, respectively,^141–144^ overlapping with the C–O and C–O binding energy ranges in both C 1s and O 1s core level regions, likely inflating the apparent C–O content under acidic conditions.

Above pH 1.2, in potassium acetate (pH 5.0) and potassium phosphate (pH 7.0) buffer electrolytes, total oxygen content was lower, consistent with the reduced ORR activity observed. This trend is explained by deprotonation of carbon oxygenates above pH 4,^124,145^ which destabilizes them thermodynamically under cathodic polarization by electrostatic repulsion. These deprotonated oxygenates were likely negatively charged, as potassium countercations were not detected (Figure S34). At pH ≥ 9.3, the total surface oxygen content was comparable to that in acidic electrolytes, although H_2_O_2_ production was negligible in acid but substantial in base (Figure 2b).

#### Computational Insights into Stability of Surface Oxygenates

To further investigate the stability of different surface oxygenates as a function of pH and electrode potential, we performed DFT calculations (Figure 3). In our simulated model structures, we take into account the possibility of C–O oxygenates in the form of ether and hydroxyl, as well as the possibility of C=O and O–C=O oxygenates in the form of carbonyl and carboxyl (Figure 3c). The reference for investigating the stability of these structures is the carbon edge saturated with hydrogen. The stability is measured by simulating the reduction of surface oxygenates to hydrogen saturated edge sites under ORR conditions. Figure 3d,e show the predicted stability of each surface oxygenate as a function of pH and potential.

Graphene fully terminated with continuous carbonyl groups is not expected to be stable under realistic conditions. The models shown in Figure 3c are therefore not intended to represent a fully relaxed or experimentally realizable graphene surface. Moreover, our model does not assume a fully continuous carbonyl network; instead, it incorporates spacing between carbonyl functional groups, which partially mitigates the instability associated with fully continuous carbonyl termination. At the same time, the exact configuration of functional groups in experimental systems is difficult to determine, and multiple structural variations may coexist. Thus, our models serve as simplified reference structures to probe the effect of applied potential and pH on the stability of individual oxygen functional groups. This approach disentangles the contributions of different functional groups and clarifies how their relative stability varies as a function of pH and applied potential.

The resulting computational analysis shows all surface oxygenates are more stable in acidic electrolytes and become less stable as the pH value increases. Figure 3e shows the calculated stability vs electrode potential, with the stability region highlighted in green. It illustrates the stability of different surface oxygenates across a potential range from negative to positive values. The experimental ORR measurements were taken within the positive potential range. Notably, at 0.7 V (the standard redox potential for 2e-ORR, indicated by the gray vertical dashed line), all oxygenates are stable (with negative formation energy), except for carboxyl, which begins to lose stability as the potential increases above 0.5 V. Because of their high stability, C–O oxygenates like ether and hydroxyl, as well as C=O oxygenates like carbonyl, are less affected by pH variation. It has been demonstrated that the combined presence of C–O (ether) and C=O (carbonyl) oxygenates synergistically promotes 2e-ORR.^25,146^ The O–C=O species, such as COOH, on the other hand, will be greatly affected by pH changes since they have the least stability of all functional groups and hence the greatest affinity to leave the surface and go to the solution. Of note, the 60 mV/pH slope indicates the redox processes are proton-coupled, meaning the number of electrons transferred is equal to the number of protons involved in the reaction. This behavior is linked to the stability regions where acids transition between their protonated and deprotonated forms as pH and potential change. For example, a carboxyl group (R-COOH) with a typical p*K*_a_ around 4–5 will exist predominantly in its protonated form (R-COOH) at pH values lower than the p*K*_a_. At pH values higher than the p*K*_a_, the carboxylate anion (R-COO^–^) becomes dominant. The potential at which the redox reaction occurs (such as during 2e-ORR) will influence the protonation state as highlighted in the Pourbaix diagram (Figure 3d). The above computational results agree with our experimental observations, confirming that O–C=O groups, such as COOH and COO, are the least abundant functional groups under 2e-ORR conditions.

We emphasize that additional structural complexity could arise from potential amorphous characteristics in carbon fibers. In our previous study, we attempted to identify evidence of such features but did not find conclusive indications.^53^ We also note that our computational model of oxygen functional groups at the edges is consistent with literature reports, which show that oxygen functional groups are highly abundant at edge sites, whereas the basal plane is generally much less favorable for their stabilization due to its low reactivity and limited availability of unsaturated sites. In certain cases, defects or dopants in the basal plane can locally enhance oxygen binding, but overall, edge sites remain the primary locations for stable oxygen functionalization.^72,147^ Lastly, we note that our models examine the intrinsic stability trends of the functional groups under different protonation states and do not represent the full electrochemical interface. Explicit solvent models can alter absolute energies, but the relative stability trends remain largely unchanged.^148^

### Mechanistic Contributions 4 and 5: Field Effects and Adsorption of Potassium Ions

To gain insights into the mechanistic role of potassium ions in the electrolyte, we investigated how K^+^ influences ORR activity and selectivity at hydrophilic carbon fiber paper catalysts. Electrolyte cations are known to alter the electrochemical double layer through field effects,^120,149^ thereby modifying the energetics of key reaction steps. In addition, cations can adsorb directly at catalytic sites, changing their accessibility and perturbing the local hydrogen-bonding structure of interfacial water. Disentangling these distinct contributions is critical for understanding the interplay between electrolyte composition and active site reactivity, and for establishing how cation effects shape the mechanistic landscape of the 2e- versus 4e-ORR pathways.

#### Role of Potassium Ions in H_2_O_2_ Production

Since the highest H_2_O_2_ production rates were observed under alkaline conditions, we evaluated the presence of potassium at cathodes after electrocatalysis in electrolytes of pH 9.3 to 14.0. The K 2s signals were analyzed instead of the stronger K 2p signals because the K 2p binding energies overlap with those of C 1s.^150^ The K 2s signals with a central binding energy of 377.7 ± 0.7 eV (Figure S36) are consistent with potassium oxygenates (C–O–K),^151^ which can serve as active sites for ORR electrocatalysis^152^ in addition to O–C=O sites.^24^ Potassium can also bind to O–C=O sites. While K 2s binding energy values for graphitic K–O–C=O sites have not been reported, the K 2p binding energies of KOC and KOOC moieties do not differ,^153^ suggesting that the K 2s binding energies of C–O–K and K–O–C=O sites are likewise not distinguishable.

Potassiation of carbon surface oxygenates decreases surface hydroxylation. Decreasing surface hydroxylation at perovskite catalysts steered the selectivity to the 4e-ORR pathway to water,^154^ which is the unwanted product in H_2_O_2_ electrosynthesis. At a more reducing potential of 0.2 V vs RHE, the 4e-ORR pathway to water was dominant. Formation of potassium oxygenates from deprotonated, negatively charged surface oxygenates stabilizes oxygenates at carbon surfaces, thus preserving surface oxygen content during electrocatalysis.^155^ To disentangle the effects of pH and K^+^ on ORR, we investigated H_2_O_2_ electrosynthesis in electrolytes of varying pH and constant K^+^ concentration of 1.0 M, achieved by addition of KCl, and in electrolytes of varying K^+^ concentration of 0.1 to 1.0 M and a constant pH of 13 (Figure S37). We chose KCl as a source of K^+^ ions because of its high aqueous solubility.^156^ The role of potassium was evaluated at applied potentials of 0.2 and 0.4 V vs RHE. These potentials were selected because the larger measured values are associated with lower relative error, which is particularly important for analyzing statistical correlations (see below).

Performance metrics, i.e., H_2_O_2_ production rate, catalytic activity (current density), and selectivity (H_2_O_2_ faradaic efficiency), obtained as a function of electrolyte pH differed between electrolytes with constant and varied K^+^ concentration, but overall showed similar trends with pH (Figure S37a). Here we analyze in more detail only the data collected in electrolytes where a single variable (either pH or K^+^ concentration, but not both) was varied. With increasing pH at all applied potentials, the H_2_O_2_ production rate and catalytic activity increased, whereas H_2_O_2_ selectivity decreased. The decrease in H_2_O_2_ faradaic efficiency was linear, with a slope of −9.8 ± 0.1 at 0.2 V and −13.8 ± 0.8 at 0.4 V vs RHE, suggesting that electrolyte pH effects are less pronounced at the more reducing potential. At the same time, the H_2_O_2_ faradaic efficiency was overall lower at the more reducing potential (Figure S37a).

At 0.2 V vs RHE, all H_2_O_2_ faradaic efficiencies in electrolytes with pH ≥ 9.3 were below 61%, indicating a contribution from the 4e-ORR to water. At pH values above 6, specific anion adsorption, particularly of hydroxide, must be considered in electrocatalytic ORR to water,^157^ as shown for Pt electrodes. Data for borate adsorption are available for Pt but not for carbon. In our pH 13.0 and 14.0 KOH electrolytes, the anions were hydroxide ions with a hydrodynamic radius of 1.10 Å,^158^ whereas in the pH 9.3 electrolyte, the anion was borate, with a larger hydrodynamic radius of 2.61 Å,^159^ hindering its specific adsorption at the cathode. Borate can specifically adsorb at Pt in acidic aqueous solution at pH ≤ 3, but at higher pH values hydroxide adsorption outcompetes borate adsorption.^160^ Our data show that at 0.2 V vs RHE, the H_2_O_2_ faradaic efficiency decreased linearly with pH at constant K^+^ concentration (Figure S37a), corroborating that ORR to water was promoted by the availability of hydroxide ions for specific cathodic adsorption at 0.2 V vs RHE, which is sufficiently reducing to access the 4-electron pathway.

In pH 13.0 electrolytes with varied K^+^ concentration, the H_2_O_2_ production rate and faradaic efficiency showed similar trends, both exhibiting a minimum at 0.5 M K^+^ (Figure S37b). The catalytic activity was independent of K^+^ concentration at 0.2 V vs RHE and reached a plateau at concentrations ≥ 0.5 M at 0.4 V vs RHE. Notably, the highest H_2_O_2_ faradaic efficiency was obtained at 0.1 M K^+^, corresponding to 0.1 M aqueous KOH, at 0.4 V vs RHE, but this occurred at the expense of overall current density (Figure S37b), consistent with H_2_O_2_ generation being a two-electron process compared to the four-electron oxygen evolution reaction.

#### Quantification of Surface Potassium

To gain deeper insights into the effect of surface potassium on H_2_O_2_ production, we quantified the surface potassium content by analyzing K 2s XPS data after ORR electrocatalysis. Here, surface K content refers to the amount of XPS-detected potassium relative to the total surface carbon (cf. section above on surface carbon oxygenates). This analysis is enabled by the binder-free design of our hydrophilic carbon fiber paper ORR catalyst, since the use of extrinsic species alters the electrified interface between the catalyst surface and the electrolyte.^161,162^

Interestingly, after electrocatalysis in pH 13.0 aqueous KOH, where the highest H_2_O_2_ selectivity was obtained with a faradaic efficiency of (95 ± 4) %, no surface potassium was detected, irrespective of the electrolyte K^+^ concentration (Figure S36). All postelectrocatalysis carbon fiber paper electrodes were cleaned using the same procedure, consisting of washing with deionized water for 5 min followed by drying in a nitrogen stream. The absence of surface K at pH 13.0 suggests that C–O–K sites did not form under these conditions, consistent with the high p*K*_a_ of sp^3^ alcohols at oxygenated carbon.^118^ This absence is unlikely to be related to the point of zero charge (pzc), which is more acidic, as inferred from reported pzc values of activated carbon and graphene oxide ranging from 3.3 to 7.5.^163–165^

In pH 9.3 electrolyte comprising a potassium borate buffer, C–O–K sites are also unlikely to form given the high p*K*_a_ of sp^3^ alcohols at oxygenated carbon.^118^ However, XPS analysis revealed a significant increase in surface O–C=O content after ORR (Figures S38 and S39), suggesting that potassiation of O–C=O (sp^3^ carboxylic acids with a p*K*_a_ of around 4)^118^ likely occurred. Such potassiation would block catalytically active sites for the 2e-ORR, explaining the inferior H_2_O_2_ electrosynthesis performance observed at pH 9.3.

By contrast, in ORR at pH 14.0 electrolyte, where surface K was detected by XPS, the surface C–O content was significantly higher than in pH 13.0 or 9.3 electrolytes (Figures S38, S40, and S41). This suggests that deprotonated hydroxyl groups, in addition to O–C=O species, were potassiated. This differentiation of potassiation sites is important for H_2_O_2_ electrosynthesis, since surface oxygenates serve as active sites for the 2e-ORR to H_2_O_2_, consistent with the highest faradaic efficiency being obtained in 0.1 M aqueous KOH (Figure 2b).

#### Statistical Correlation with ORR Performance

To visualize correlations between performance metrics and surface composition, we constructed bivariate plots of surface K, O–C=O, and total O content as a function of electrolyte pH and K^+^ concentration (Figure S38). In addition, we statistically correlated XPS-derived surface K, O–C=O, and total O contents with ORR performance metrics across electrolyte conditions. The resulting Pearson product–moment correlation coefficients showed similar trends, with stronger statistically significant correlations at the more reducing potential of 0.2 V than at 0.4 V vs RHE (Figure 4). The threshold for statistically significant correlation is an absolute Pearson coefficient of 0.5, consistent with the literature.^166^

**Figure 4:**
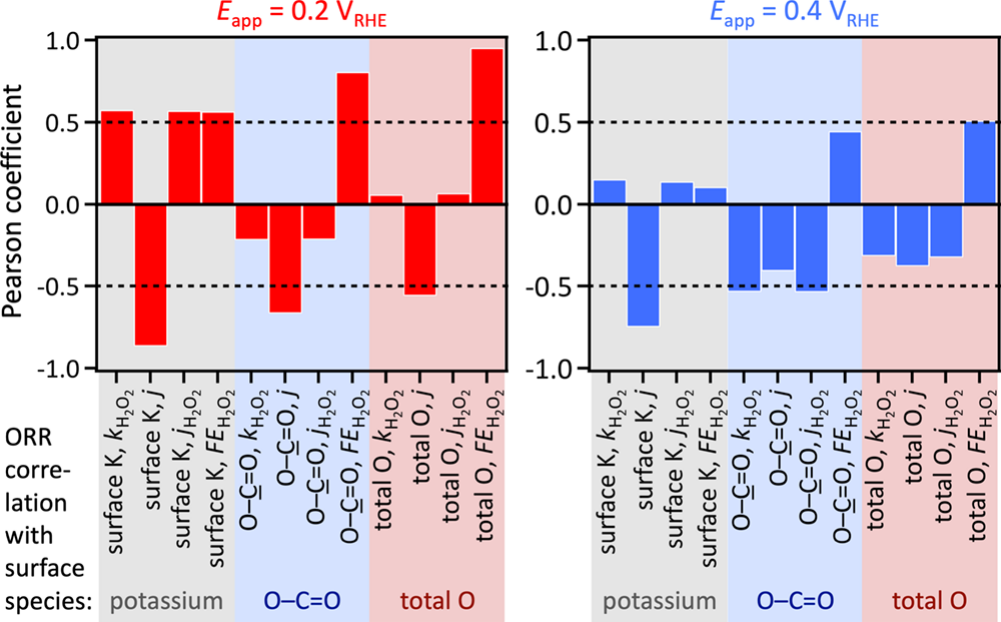
Statistical correlations of ORR performance metrics with post-ORR catalyst surface species, measured in electrolytes with systematically varied pH values and K^+^ concentrations. Dashed lines indicate the commonly accepted threshold for statistical significance.^166^ *k*_H_2_O_2__, H_2_O_2_ production rate; *j*_H2O2_, H_2_O_2_ partial current density.

At 0.2 and 0.4 V vs RHE, a strong anticorrelation was observed between the surface K, O–C=O, and total O contents and the total current density, which includes contributions from both 2e- and 4e-ORR. The anticorrelation of surface K content with current density suggests active site blocking by adsorbed potassium and indicates that no 2e-ORR promotion occurs when K is bound to the surface and detectable by XPS after electrocatalysis. However, promoting field effects of K^+^ ions in the interfacial microenvironment may still operate,^120,149^ provided that potassium does not adsorb directly on the surface, where its presence can be detected by XPS.

In the context of H_2_O_2_ electrosynthesis, H_2_O_2_-specific performance metrics, such as faradaic efficiency, partial current density, and production rate, are most relevant. The surface O–C=O content, and even more strongly the total O content, correlated positively with H_2_O_2_ faradaic efficiency, particularly at 0.2 V vs RHE, consistent with earlier reports identifying O–C=O functional groups as the primary active sites for the 2e-ORR to H_2_O_2_.^24^ Surface K content also correlated positively with H_2_O_2_ faradaic efficiency at 0.2 V, but not at 0.4 V vs RHE. Similarly, surface K content correlated positively with H_2_O_2_ partial current density and production rate at 0.2 V, but not at 0.4 V vs RHE. These results suggest that at the more reducing potential, where the 4e-ORR contributes significantly, surface K can suppress the undesired 4-electron pathway without potassiating H_2_O_2_ active sites.

While surface O–C=O and total O contents showed a strong positive correlation with H_2_O_2_ faradaic efficiency, consistent with their role as active sites for H_2_O_2_ generation,^24^ they also exhibited anticorrelation with the total current density at both applied potentials, as well as with the H_2_O_2_ partial current density and production rate at 0.4 V vs RHE. Current and rate reflect activity, whereas faradaic efficiency reflects selectivity. These results suggest that although surface O–C=O groups serve as active sites that promote H_2_O_2_ selectivity, a high density of these sites decreases activity, likely because significant aqueous solvent reorganization is required due to disruption of the interfacial water structure at the catalyst surface. Such reorganization imposes a kinetic barrier that lowers activity. Computational studies have shown that O–C=O groups on graphene disrupt the structure of adsorbed water more strongly than alcohol groups.^167^ Our results suggest that an optimal number of active O–C=O sites exists as a trade-off between activity and selectivity, consistent with our observation at 0.4 V of the highest selectivity (H_2_O_2_ faradaic efficiency) in 0.1 M aqueous KOH and the highest activity (H_2_O_2_ production rate) in 1.0 M KOH, where the surface O–C=O content was 2.5 times higher than in 0.1 M KOH (Figure S42).

#### Computational Insights into Understanding K^+^ and pH Effects

Many different hypotheses have been proposed to computationally understand the pH effect. For example, it has been hypothesized that the kinetic barriers of the competing 4e-ORR to water are higher in alkaline than acidic water due to the shift in the proton source.^5,17,18,168^ For weak binding surfaces such as metallic gold, it has been shown that kinetic barriers of the ORR to H_2_O_2_ depend on the electrolyte pH in strongly acidic media, with theoretically predicted overall barrier heights decreasing as pH values increase.^89^ This was suggested to be due to the field effect arising from the concentrated cations in the alkaline solution. Computational ab initio molecular dynamics (AIMD) simulation on alkali metal cations such as Na^+^ shows that adsorption of cations on the electrode surface creates a local coordination environment that drives H^+^ atoms away from the surface and therefore reduces the H_2_O_2_ reduction reaction (H_2_O_2_RR).^89^ The local electric field effect induced by cations has been recently studied on various carbon defects and functional groups using DFT calculations.^120^ More positive applied local electric field is a manifestation of the increased cation concentration under alkaline pH. The strength of the local electric field originating from solvated K^+^ in the vicinity of the catalysts surface was reported to be in the range 0.60–0.65 eV.^120^ The computational results indicated the stabilization of OOH*, the key intermediate of 2e-ORR to H_2_O_2_, in the presence of a positive applied local electric field arising from concentrated cations at the surface which in turn increases the selectivity toward H_2_O_2_.^120^ Another hypothesis is that in base, the kinetic barriers preventing *H_2_O_2_ or *OOH dissociation are lower than in acid.^5^ Ergo, faradaic efficiencies for H_2_O_2_ production should increase as pH values increase and applied potentials are more reducing. However, we experimentally observe decreasing H_2_O_2_ faradaic efficiencies with increasing pH and more reducing potentials when K^+^ concentrations are kept constant (Figure S37). Furthermore, the onset potentials increase linearly with pH (Figures 3a and S12), which does not support the hypothesis of a mechanistic change when switching from acidic to alkaline conditions, at least at low overpotentials.

## Conclusions

We developed a rapid and environmentally benign process to transform hydrophobic commercial carbon fiber paper into a hydrophilic, binder-free ORR catalyst by introducing surface oxygenates at graphitic edges. The resulting porous carbon fiber network retained structural integrity while exhibiting extended hydrophilicity and high surface area, enabling adsorption of interfacial water and stable H_2_O_2_ electrosynthesis. The hydrophilic carbon fiber paper cathodes outperformed previously reported carbon catalysts, achieving a maximum faradaic efficiency of (95 ± 4) % for ORR to H_2_O_2_ at 0.4 V vs RHE in O_2_-saturated 0.1 M pH 13.0 aqueous KOH and maintaining activity without structural degradation or stabilizing agents for more than 31 h in a divided cell and 100 h in an undivided cell.

The binder-free design enabled combined experimental and computational mechanistic analysis without binder-induced, pH-dependent interfacial changes confounding interpretation. Analysis of onset potentials revealed a linear dependence on electrolyte pH with a slope of (0.055 ± 0.009) V per pH unit on the RHE scale, indicating a non-PCET mechanism. This behavior likely arises from a combination of active site stability, field effects of interfacial potassium ions, and active site blocking by potassiation of surface carbon oxygenates. In addition, the observed slope of 0.114 V vs SHE indicates that the rate-limiting step is proton dependent, consistent with computational predictions.

Mechanistic studies further showed that H_2_O_2_ production depends sensitively on electrolyte pH and K^+^ concentration. XPS analysis correlated performance metrics with surface species, revealing that while O–C=O groups serve as primary active sites for the 2e-ORR, excessive coverage decreases activity, likely by disrupting interfacial water structure. Potassium played a dual role: surface-adsorbed potassium blocked active sites and suppressed overall activity, whereas solvated K^+^ in the interfacial microenvironment promoted H_2_O_2_ selectivity by inhibiting the competing 4e-ORR pathway. These findings highlight the importance of distinguishing surface-bound from interfacial cations in rationalizing ORR performance.

Finally, higher H_2_O_2_ production rates at elevated pH were attributed to the superior stability of C–O and C=O oxygenates, supported by XPS and DFT stability analysis. Together, these results establish that optimized balances of surface O–C=O content and interfacial K^+^ effects are required to maximize both selectivity and activity. Hydrophilic carbon fiber paper thus emerges as a robust, sustainable, binder-free platform for H_2_O_2_ electrosynthesis, offering mechanistic insights into the coupled roles of oxygenated carbon sites, electrolyte pH, and spectator ions in steering ORR pathways.

## Experimental Section

All chemicals were used as received. Deionized water was obtained from a Thermo Scientific Barnstead Smart2Pure Pro UV/UF 15 LPH Water Purification System and had a resistivity of ≥17.5 MΩ·cm. All experiments were performed at room temperature. Data analysis and graphing were performed with Igor Pro 8.04 (Wavemetrics).

### Catalyst Preparation

The process to make carbon fiber paper hydrophilic is described elsewhere.^51,53^ Briefly, we selectively functionalized surfaces of as-purchased carbon fiber paper (FuelCellStore, AvCarb MGL190) by sonication in 1 M aqueous sodium dodecyl sulfate solution, followed by electrooxidation in 0.1 M pH 8.7 aqueous KHCO_3_ electrolyte at +1.63 V vs Ag/AgCl for 20 min.^51,53^ Cathodes used for ORR had geometric dimensions of 3.5 cm (length) × 2.3 cm (width), amounting to a geometric electrode area of 8.05 cm^2^.

### Physical Characterization

X-ray photoelectron spectra (XPS) were collected with a Kratos Axis Ultra XPS instrument at UR-Nano, which was equipped with a monochromatized Al Kα radiation source, operated in high-power mode at 200 W and 15 kV, with a base chamber pressure of 3.0 × 10^–8^ mbar. Samples were immobilized on double-sided adhesive copper tape. Survey scans were obtained between 0 and 1200 eV with a step size of 1 eV, a dwell time of 200 ms, and an analyzer pass energy of 140 eV averaged over 5 scans. Core level region scans for C 1s, O 1s, Cl 2p, and K 2s regions were obtained at the corresponding binding energy ranges with a step size of 0.1 eV, an average dwell time of 260 ms, and an analyzer pass energy of 20 eV averaged over 5 scans. Binding energies were referenced to the C 1s peak arising from adventitious carbon, taken to have a binding energy of 284.8 eV.^169^ Binding energies and quantitative peak areas were derived after Shirley background subtraction^170^ and Gaussian/Lorentzian envelope peak fitting. For the quantification of different components, instrument-specific atomic sensitivity factors determined from standard materials were used. XPS analysis was performed with CasaXPS (Version 2.3.24). More details regarding the analysis of carbon fiber paper XPS data are in ref 53.

Scanning electron microscopy (SEM) images were acquired with a JEOL JSM-5900LV SEM instrument equipped with a thermionic tungsten electron gun, operated at 25 kV with a working distance of 10 mm. Select SEM images were collected at UR-Nano, using a Zeiss Auriga scanning electron microscope, equipped with a Schottky field emission emitter, and operated at 20.00 kV with a working distance of 5.1 mm. Carbon fiber paper samples were immobilized on 1-in. diameter aluminum SEM stubs (Ted Pella) with carbon tape (Electron Microscopy Sciences).

### Electrocatalysis

#### Electrochemical Setup

We used two virtually identical sealed electrochemical H-type replaceable membrane electrolytic cells in parallel, together with two potentiostats (CHI660); one cell was purged with oxygen gas (Airgas), while the other was purged with argon (Airgas). The cell bodies were made of glass, and their Teflon lids and electrode feed-throughs were sealed with O-rings; septa sealed gas inlets and pressure relief needles. Each H-cell was purged with gas of virtually the same pressure in both compartments, and pressure relieve needles to the ambient air ensured 1 atm pressure conditions in both compartments. Gas bubbles were supplied to the liquid by plastic pipet tips, to prevent metal contamination of the electrolyte from a needle. Each cell was purged with gas for 30 min before experiments for data acquisition were started. The two compartments in each H-cell were separated by a Nafion 117 membrane (Sigma-Aldrich) for electrolytes with pH values from 0 to 13 or a Selemion anion exchange membrane for pH 14 electrolyte. Each compartment was filled with 50 mL electrolyte, and each compartment was stirred at 350 rpm on a multiposition stir plate (G-Biosciences BT1016). Hydrophilic carbon fiber paper served as counter electrode material. Experiments were conducted with a Pt wire pseudoreference electrode that was calibrated in each electrolyte against a hydrogen reference electrode (Gaskatel HydroFlex). This calibration was performed by measuring the open-circuit potential for 12 h in O_2_-purged electrolyte stirred at 350 rpm, using a two-electrode setup in a 30 mL beaker in which the RHE electrode served as the reference electrode and the Pt wire served as the working electrode. The potential stabilized after 5 min, and the averaged value thereafter was taken as the calibration value. Platinum wire has been shown to be a suitable and stable reference electrode in various electrochemical systems.^171^

#### Electrolytes

A Thermo Scientific Accumet Excel XL20 pH meter was used to measure electrolyte pH values. Aqueous 1.0 M pH 0.6 HClO_4_, 0.1 M pH 1.2 HClO_4_, 0.1 M pH 5.0 potassium acetate buffer, 0.1 M pH 7.0 potassium phosphate buffer, 0.1 M pH 9.3 potassium borate buffer, 0.1 M pH 13.0 KOH, or 1.0 M pH 14.0 KOH served as electrolytes. The aqueous 1.0 or 0.1 M HClO_4_ electrolytes were purchased from Honeywell or Grainger (NIST certified), respectively. Buffers were prepared on the day of use. For the 0.1 M pH 5.0 potassium acetate buffer, 9.82 g potassium acetate (Sigma-Aldrich, ≥ 99%) were added to a 1 L volumetric flask that was subsequently filled to the 1 L mark with water; glacial acetic acid (Mallinckrodt) was titrated until the solution reached a pH of 5.0. For the 0.1 M pH 7.0 potassium phosphate buffer, 9.344 g of monobasic potassium phosphate (Fisher Scientific, Certified ACS) and 9.308 g of dibasic potassium phosphate (Fisher Scientific, Certified ACS) were added to a 1 L volumetric flask that was subsequently filled to the 1 L mark with water; dibasic potassium phosphate was added until the solution reached a pH of 7.0. For the 0.1 M pH 9.3 potassium borate buffer, 30.55 g of potassium tetraborate tetrahydrate (Sigma-Aldrich, ≥99%) were added to a 1 L volumetric flask that was subsequently filled to the 1 L mark with water; boric acid (Fisher, Certified ACS) was added until the solution reached a pH of 9.3. The aqueous 1.0 or 0.1 M KOH electrolytes were prepared by adding 56.12 or 5.612 g of KOH (Thermo Scientific, 99.98%), respectively, to a 1 L volumetric flask that was subsequently filled to the 1 L mark with water.

Electrolytes of varying pH and constant K^+^ concentration of 1.0 M were prepared by adding 0.8 M KCl to 0.1 M pH 9.3 potassium borate buffer and 0.9 M KCl to 0.1 M pH 13.0 KOH. As 1.0 M pH 14.0 KOH already contained 1.0 M K^+^, no KCl was added. The aqueous 0.1 M pH 9.3 potassium borate buffer, 0.1 M pH 13.0 KOH, and 1.0 M pH 14.0 KOH electrolytes were prepared as described above. The aqueous 0.1 M pH 9.3 potassium borate buffer with 0.8 M KCl electrolyte was prepared by adding 59.641 g of potassium chloride (Mallinckrodt) to a 1 L volumetric flask containing 0.1 M pH 9.3 potassium borate buffer, while the 0.1 M pH 13.0 KOH with 0.9 M KCl electrolyte was prepared by adding 67.096 g of potassium chloride (Mallinckrodt) to a 1 L volumetric flask containing 0.1 M pH 13.0 KOH. For electrolytes of varying K^+^ concentration of 0.1 to 1.0 M and a constant pH of 13.0, 0.4, and 0.9 M KCl was added to 0.1 M pH 13.0 KOH to obtain 0.5 and 1.0 M K^+^, respectively. As 0.1 M pH 13.0 KOH already contained 0.1 M K^+^, no KCl was added. The aqueous 0.1 M pH 13.0 KOH electrolyte was prepared as described above. The 0.1 M pH 13.0 KOH with 0.4 M KCl electrolyte was prepared by adding 29.821 g of potassium chloride (Mallinckrodt) to a 1 L volumetric flask containing 0.1 M pH 13.0 KOH, while the aqueous 0.1 M pH 13.0 KOH with 0.9 M KCl electrolyte was prepared as described above.

#### Electrochemical Data Acquisition and Analysis

All potentials are reported vs RHE. Linear sweep voltammograms were collected in each O_2_- or Ar-saturated electrolyte at a scan rate of 10 mV s^–1^ in an applied potential range of 0.0 to 0.9 V vs RHE. Onset potentials were obtained as the intersect of tangent lines from rising currents and baseline currents. Chronoamperometry data were collected in each electrolyte for 2 h at three applied potentials (0.2, 0.4, and 0.6 V vs RHE) in H-cells that were cleaned with aqua regia between electrolytes. Electrocatalysis experiments in electrolytes with added KCl were performed under a fume exhaust because of anodic generation of toxic chlorine gas. After 5, 15, 30, 60, 90, and 120 min of electrocatalysis, electrolyte aliquots of 0.2 mL were collected from the working electrode compartment through a septum for H_2_O_2_ quantification.

Long-term stability testing in the H-cell was performed in O_2_-saturated aqueous 0.1 M pH 13.0 KOH electrolyte at an applied potential of 0.4 V vs RHE for 31 h; 0.2 mL aliquots for H_2_O_2_ quantification were collected every 5 h. Long-term stability testing in the undivided cell was conducted in O_2_-saturated aqueous 0.1 M pH 13.0 KOH electrolyte stirred at 350 rpm at an applied potential of 0.4 V vs RHE for 100 h in a 50 mL beaker covered with a Teflon lid equipped with O-ring-sealed electrode feed-throughs and a septum-sealed O_2_ inlet and pressure-relief needle. Gas bubbles were introduced into the liquid through a plastic pipet tip, and the electrolyte was purged with O_2_ for 30 min before data acquisition began. Hydrophilized carbon fiber paper with geometric dimensions of 3.5 cm × 2.3 cm served as both working and counter electrodes, spaced 16 mm apart, and a Pt pseudoreference electrode was used. Because of timeout limitations of the CHI660 potentiostat after 36 h, the chronoamperometry experiment was restarted every 24 h, with a 5 s period at open circuit potential after each 24 h interval.

### Electrochemical Impedance Spectroscopy

Electrical impedance spectroscopy (EIS) data were collected at open circuit potential in aqueous 0.1 M LiClO_4_-supported acetonitrile electrolyte, using a Bio-Logic potentiostat (8-slot VSP3e potentiostat/galvanostat/EIS system). A standard one-compartment three-electrode setup was used with hydrophilized or untreated carbon fiber paper as working electrode, untreated carbon fiber paper as counter electrode, and a Pt pseudoreference electrode. Working and counter electrodes each had geometric dimensions of 3.5 cm × 2.3 cm, and the interelectrode distance was 16 mm. The sinusoidal perturbation for EIS was set to an amplitude of 10 mV, with a frequency range spanning from 100 kHz to 1 Hz, in keeping with the literature.^40,41,70^ The resolution was set to 10 points per decade with each point being an average of three measurements. The EIS data were fitted and analyzed using the Bio-Logic EC-Lab software package, which provided the stated errors in resistance values. The electrical conductivity σ in units of S m^–1^ was calculated from the resistance *R* in units of Ω, the interelectrode distance *L* in units of m, and the electrode area *A* in units of m^2^, using the following equation:

$$\sigma =\frac{1}{R(\Omega )}\times \frac{L(m)}{A(m^{2})}=\frac{1}{R(\Omega )}\times \frac{0.016\;m}{0.0805\;m^{2}}$$

### Quantification of H_2_O_2_

Cerium­(IV) sulfate titration monitored by spectrophotometry was employed to quantify H_2_O_2_ production in each electrolyte (Figures S43–S52). Stock solutions of 0.10 or 0.16 M Ce­(SO_4_)_2_ (Thermo Scientific, 99%) in 0.5 M aqueous H_2_SO_4_ (Fisher Chemical) were prepared. Calibration curves were measured for each electrolyte, using known concentrations of Ce­(SO_4_)_2_ in 0.5 M aqueous H_2_SO_4_, and covering the H_2_O_2_ concentration range of electrocatalysis experiments (Figures S43–S45). Optical spectra (Figures S46–S52) were measured using 1 cm path length quartz cuvettes and a fiber-optic ultraviolet to near-infrared optimized spectrometer (OCEAN-HDX-XR). Air blanks were used, and spectra of neat electrolytes were collected and subtracted from spectra of solutions of the respective electrolytes that contained Ce­(SO_4_)_2_. Spectra were integrated in the 260–447 nm range for improved accuracy over previously reported single-wavelength measurements.^25^ Formed Ce­(III) has a peak absorbance of 265 nm. We compared spectral integration in the range of 260–447 nm to that of 280–447 nm, where Ce­(III) does not absorb, using data for our highest performing conditions, at pH 13 at 0.4 V_RHE_, which showed a large bleach (Figure S51). We did not find a significant difference in deduced H_2_O_2_ production data, indicating that the Ce­(III) absorption did not affect our results. Aliquots of 0.2 mL of working electrode compartment liquid were added to 1, 2, 3, 5, 8, 10, 15, 20, or 25 mL of the Ce­(SO_4_)_2_ stock solution, ensuring an observable bleach in the measured optical spectra within the calibration range and a pH value of the mixture below 3.2 to prevent precipitation of cerium-containing solid; 1 mL of the analyte plus Ce­(SO_4_)_2_ solution were used in the cuvette.

Based on the observed linear relationship of spectral features in the 260–447 nm range (Figures S46–S52s) and known initial Ce^4+^ concentrations in the calibration curves, H_2_O_2_ concentrations were calculated from Ce^4+^ concentrations, using the Beer–Lambert law and the equation [H_2_O_2_] = $\frac{1}{2}$ × [Ce^4+^], because H_2_O_2_ bleaches the yellow Ce^4+^ to colorless Ce^3+^ according to the chemical eq 2 Ce^4+^ + H_2_O_2_ → 2 Ce^3+^ + 2 H^+^ + O_2_.^25^ We note that in ref 25 the concentration of H_2_O_2_ was calculated as [H_2_O_2_] = 2 × [Ce^4+^], which is inconsistent with the chemical equation stated in ref 25 (2 Ce^4+^ + H_2_O_2_ → 2 Ce^3+^ + 2 H^+^ + O_2_), inflating H_2_O_2_ concentration numbers in ref 25 by a factor of 4; as a consequence, H_2_O_2_ production rates in ref 25 are also inflated by a factor of 4.

The hydrogen peroxide production rate *k*_H2O2_ in units of mg L^–1^ h^–1^ was calculated from the H_2_O_2_ concentration [H_2_O_2_] in units of mol L^–1^, using the equation:

$$k_{H_{2}O_{2}}=[H_{2}O_{2}]\times 34.0147\times 10^{3}\;(\mathrm{mg}\;{\mathrm{mol}}^{-1})\times \frac{1}{2\;h}$$

### Calculation of Faradaic Efficiencies

Faradaic efficiencies (FE) were calculated from molar concentrations of produced H_2_O_2_ in combination with the average charge transferred during the 2 h of electrocatalysis, using the following equation:

$$\begin{array}{ll} \mathrm{FE}(\%) & =\frac{N_{\mathrm{product}}}{N_{\mathrm{total}}}=\frac{c_{\mathrm{product}}(\mathrm{ppm})\cdot n_{e}\cdot F(C\;{\mathrm{mol}}^{-1})\cdot 0.05\;L}{M(\mathrm{mg}\;{\mathrm{mol}}^{-1})\cdot |I_{\mathrm{average}}|(A)\cdot t(s)}\\ & =\frac{c_{\mathrm{product}}(\mathrm{ppm})\cdot n_{e}\cdot 19.69\times 10^{-6}\;A\;L\;{\mathrm{mg}}^{-1}}{\left|I_{\mathrm{average}}|(A\right)} \end{array}$$

In this equation, *N*_product_ is the number of electrons transferred to make H_2_O_2_, *N*_total_ is the total number of electrons transferred, *c*_product_ is the molar concentration of produced H_2_O_2_ product in units of ppm (equal to mg L^–1^), *n*_e_ is the number of electrons required to reduce one molecule of O_2_ to one molecule of H_2_O_2_ (equal to 2), *F* is Faraday constant (equal to 96485.34 C mol^–1^), *M* is the molar mass of H_2_O_2_ (equal to 34.0147 × 10^3^ mg mol^–1^), *I*_average_ is average current during electrocatalysis in units of A, and *t* is the total time of electrolysis in units of seconds. The constant parameters in the equation led to the number 19.69 × 10^–5^ in units of A L mg^–1^; this number was derived from multiplying the Faraday constant (96485.34 C mol^–1^) with the volume of electrolyte present in the cell (0.05 L), dividing by the molar mass of H_2_O_2_ (34.0147 × 10^3^ mg mol^–1^), and then dividing by the total time of electrolysis (7200 s).

### Statistical Analysis

Pearson product–moment correlation coefficients (*r*) for two data sets *X* and *Y*, each with n entries, were calculated using the following equation, where *X*_*n*_ and *Y* are the values of the *x*- and *y*-variables and X̅ and Y̅ are their respective means:^166^

$$r=\frac{\sum_{n}(X_{n}-X®)(Y_{n}-Y®)}{\sqrt{\sum_{n}(X_{n}-X®{)}^{2}}\sqrt{\sum_{n}(Y_{n}-Y®{)}^{2}}}$$

### Computational Details

Atomic Simulation Environment (ASE)^172^ and QUANTUM ESPRESSO program package^173^ were used to handle the simulations and perform the electronic structure calculations, respectively. The electronic wave functions were expanded in plane waves up to a cutoff energy of 500 eV, while the electron density is represented on a grid with an energy cutoff of 5000 eV. Core electrons are approximated using GBRV ultrasoft pseudopotentials.^174^ Perdew–Burke–Ernzerhof (PBE) exchange-correlation functional^175^ with dispersion corrections^176^ was used, which has been shown to accurately describe the formation energies of each model structure. Carbon-edge structures were modeled using as one layer nanoribbon of graphene. A vacuum region of 20 Å was used to decouple the periodic replicas. A supercell of lateral sizes 4 × 3 was used, and the Brillouin zones were sampled with (1 × 4 × 1) Monkhorst–Pack k-points, respectively.

## Supplementary Material



## Notes

Notes on ORR mechanism and product and catalyst characterization data (PDF)

## Abbreviations

2e-ORR: two-electron–two-proton oxygen reduction reaction
4e-ORR: four-electron–four-proton oxygen reduction reaction
AIMD: *ab initio* molecular dynamics
ASE: atomic simulation environment
DFT: density functional theory
*E*_app_: applied potential
*FE*: faradaic efficiency
H_2_O_2_RR: H_2_O_2_ reduction reaction
*j*_H_2_O_2__: H_2_O_2_ partial current density
*k*_H_2_O_2__: H_2_O_2_ production rate
NHE: normal hydrogen electrode
ORR: oxygen reduction reaction
PBE: Perdew–Burke–Ernzerhof
PFAS: per- and polyfluoroalkyl substances
RHE: reversible hydrogen electrode
RRDE: rotating ring disk electrochemistry
SEM: scanning electron microscopy
SHE: standard hydrogen electrode
XPS: X-ray photoelectron spectroscopy
