# Supplementary material for: Oxygen Reduction to Hydrogen Peroxide on Hydrophilic Carbon Fiber Paper: Dependence of the Mechanism and Active Site Stability on Electrolyte pH and Potassium Ion Concentration
Source: ACS Catal. 2026 Mar 26;16(8):7376–94. doi: 10.1021/acscatal.5c08719 (PMC13097141; doi:10.1021/acscatal.5c08719)
Supplement: Supplementary file 1 [file cs5c08719_si_001.pdf]

# Supporting Information

# Oxygen Reduction to Hydrogen Peroxide on Hydrophilic Carbon Fiber Paper: Dependence of Mechanism and Active Site Stability on Electrolyte pH and Potassium Ion Concentration

Connor P. Cox,[1,†] Madeleine K. Wilsey,[1,†] Kendra R. Watson,[2] Teona Taseska,[2] Yiwen Sun,[2] Lydia R. Schultz,[2] Samira Siahrostami,*[3] Astrid M. Müller*[1,2]

[1] Materials Science Program, University of Rochester, Rochester, New York 14627, United States.
[2] Department of Chemical Engineering, University of Rochester, Rochester, New York 14627, United States.
[3] Department of Chemistry, Simon Fraser University, Burnaby, BC V5A 1S6, Canada.

[†] These authors contributed equally.

* Email: samira_siahrostami@sfu.ca; astrid.mueller@rochester.edu.

# NOTES

## $H_2O_2$ production

$H_2O_2$ production rates are often measured in aqueous 0.1 M pH 13.0 KOH electrolyte (Table S1). $H_2O_2$ generation depends on the electrochemical cell geometry. Higher selectivity for $H_2O_2$ has been reported in rotating ring disk electrochemistry (RRDE) experiments, in which mass transport of product species away from the cathode is optimized, compared to in H-cell geometries and membrane electrode assemblies.[1,2] RRDE experiments require a flat electrode surface to comply with the assumptions and mass transport equations of axial laminar flow at the electrode surface, to reliably deduce the number of transferred electrons from measured data.[3]

**Table S1.** Comparison of $H_2O_2$ production rates and faradaic efficiencies of this work with those of reported carbon catalysts.

| Material | $E_{app}$ (V vs RHE) | Production Rate (mg $L^{-1}$ $h^{-1}$) | Faradaic Efficiency (%) | Conditions | Ref. |
|---|---|---|---|---|---|
| Hydrophilic carbon fiber paper | 0.40 | 49 ± 4 | 95 ± 4 | H-cell, aq 0.1 M KOH electrolyte, pH 13 | This work |
| Hydrophilic carbon fiber paper | 0.60 | 108 ± 4 | 79 ± 4 | H-cell, aq 1.0 M KOH electrolyte, pH 14 | This work |
| Graphitic nanoplatelets | 0.65 | 6.92 | 95 | H-cell, aq 0.1 M KOH electrolyte, pH 13 (400 ppm $MgSO_4$ added to stabilize $H_2O_2$) | 4 |
| Few-layered reduced graphene oxide | 0.63 | Not reported at 0.63 V vs RHE | 100 | H-cell, aq 0.1 M KOH electrolyte, pH 13, 800 ± 20 torr $O_2$ | 5 |
| Oxidized carbon nanotubes | −1.45 | 988* | Not reported | Gas diffusion electrode, aq 1.0 M KOH electrolyte, pH 14 | 6 |
| Carbon black | −0.60 | 384.62 | 75% – 95% during 13-hour cycle | Gas diffusion electrode with solid electrolyte configuration | 7 |
| Honeycomb carbon nanofibers | 0.50 | 216.67 | 90 | Flow cell, aq 0.1 M KOH electrolyte, pH 13 | 8 |
| Anodized graphite felt | 0.26 | 210 | Not reported | Flow cell in static mode, aq 0.05 M $Na_2SO_4$ electrolyte, pH 7 | 9 |
| Reticulated vitreous carbon | −0.026 | 15.87 | Not reported | Flow cell, aq 0.50 M $Na_2SO_4$ electrolyte, pH 3 | 10 |
| Carbon black | 0.52 | Not reported | 100 | Rotating ring disk electrode, aq 0.1 M KOH electrolyte, pH 13 | 11 |
| Carbon-PTFE composite on rotating cylindrical carbon cloth cathode | −0.46 | 59.1 | Not reported | Rotating cylinder electrode reactor with multiple anodes, aq 0.05 M $Na_2SO_4$ electrolyte, pH 3 | 12 |
| Carbon black and PTFE on carbon fiber paper | +1.46 | 204.5 | 99.8 during the first 30 min | One-compartment cell, aq 0.05 M $Na_2SO_4$ electrolyte, pH 7 | 13 |
| Reduced graphene oxide synthetic fabric, treated with $NaBH_4$ and $H_2SO_4$ | −0.42 | 16.33 | 63.9 | One-compartment cell, aq 0.05 M $Na_2SO_4$ electrolyte, pH 6.5 | 14 |

| Material | $E_{app}$ (V vs RHE) | Production Rate (mg L$^{-1}$ h$^{-1}$) | Faradaic Efficiency (%) | Conditions | Ref. |
|---|---|---|---|---|---|
| Graphene foam | −0.236 | 25.5 | Not reported | One-compartment cell, aq 0.05 M $K_2SO_4$ electrolyte, pH 3 | 15 |
| Polypyrrole – multi-walled carbon nanotube nanocomposite | −0.13 | 33.6 | Not reported | One-compartment cell, aq 0.10 M $Na_2SO_4$ electrolyte, pH 3 | 16 |
| Carbon felt | −0.48 | ca. 90 | 30 | One-compartment cell, aq 0.05 M $Na_2SO_4$ electrolyte, pH 3 | 17 |
| Activated carbon fiber | 0.20 | 18.9 | 64.5 | One-compartment cell, aq 0.05 M $Na_2SO_4$ electrolyte, pH 3 | 18 |
| Anodized graphite felt | −0.03 | 110 | 79.1 | One-compartment cell, aq 0.05 M $Na_2SO_4$ electrolyte, pH 6.4 | 19 |
| Anodized graphite felt | 0.00 | 187.5 | Not reported | One-compartment cell, aq 0.05 M $Na_2SO_4$ electrolyte, pH 3 | 20 |
| Anodized graphite felt | −0.18 | 81.65 | 42.4 | One-compartment cell, aq 0.05 M $Na_2SO_4$ electrolyte, pH 3 | 21 |
| Anodized graphite felt | −0.43 | 121.2 | 78 | One-compartment cell, aq 0.05 M $Na_2SO_4$ electrolyte, pH 3 | 22 |
| Anodized graphite felt | −0.18 | 55.25 | 65 | One-compartment cell, aq 0.05 M $Na_2SO_4$ electrolyte, pH 3 | 23 |
| *Tert*-butyl-anthraquinone modified vitreous carbon | −1.00 V vs SCE | 200.67 | 89.6 | One-compartment cell, aq 0.10 M $K_2SO_4$ + 0.10 M $H_2SO_4$ electrolyte, pH not reported | 24 |

* $H_2O_2$ production rates in ref. 6 are inflated by a factor of 4 because of an error in concentration calculations. We report here the correct number based on the data of ref. 6.

## FIGURES

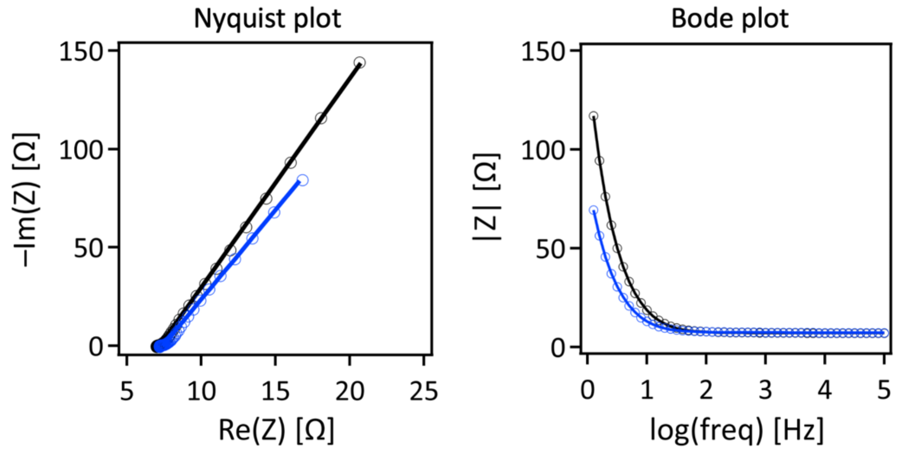


**Figure S1.** EIS data of untreated (black) and hydrophilized (blue) carbon fiber paper.

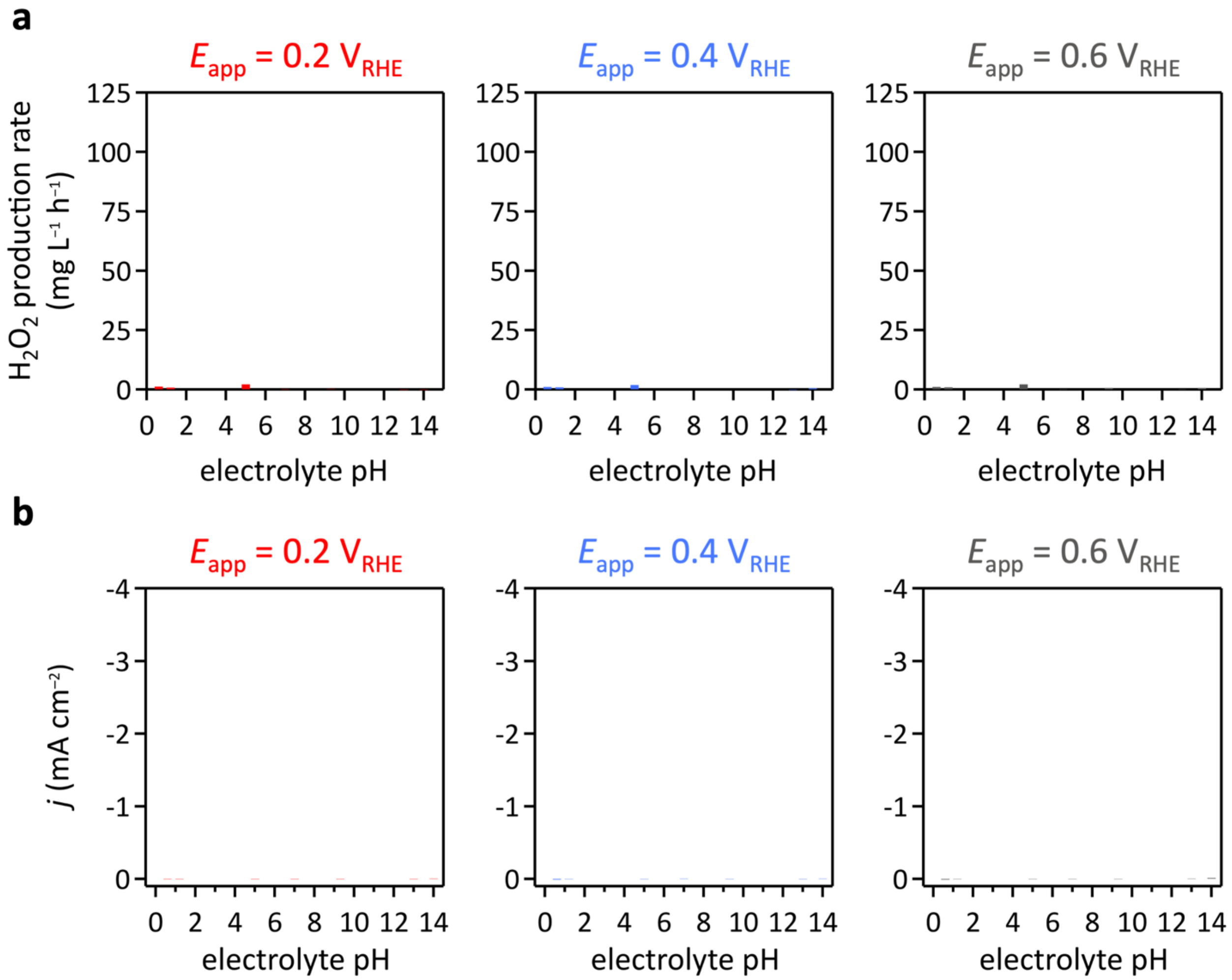


**Figure S2.** Performance. (a) Hydrogen peroxide production rates or (b) generated current densities as a function of applied potential and electrolyte pH in Ar-saturated electrolytes.

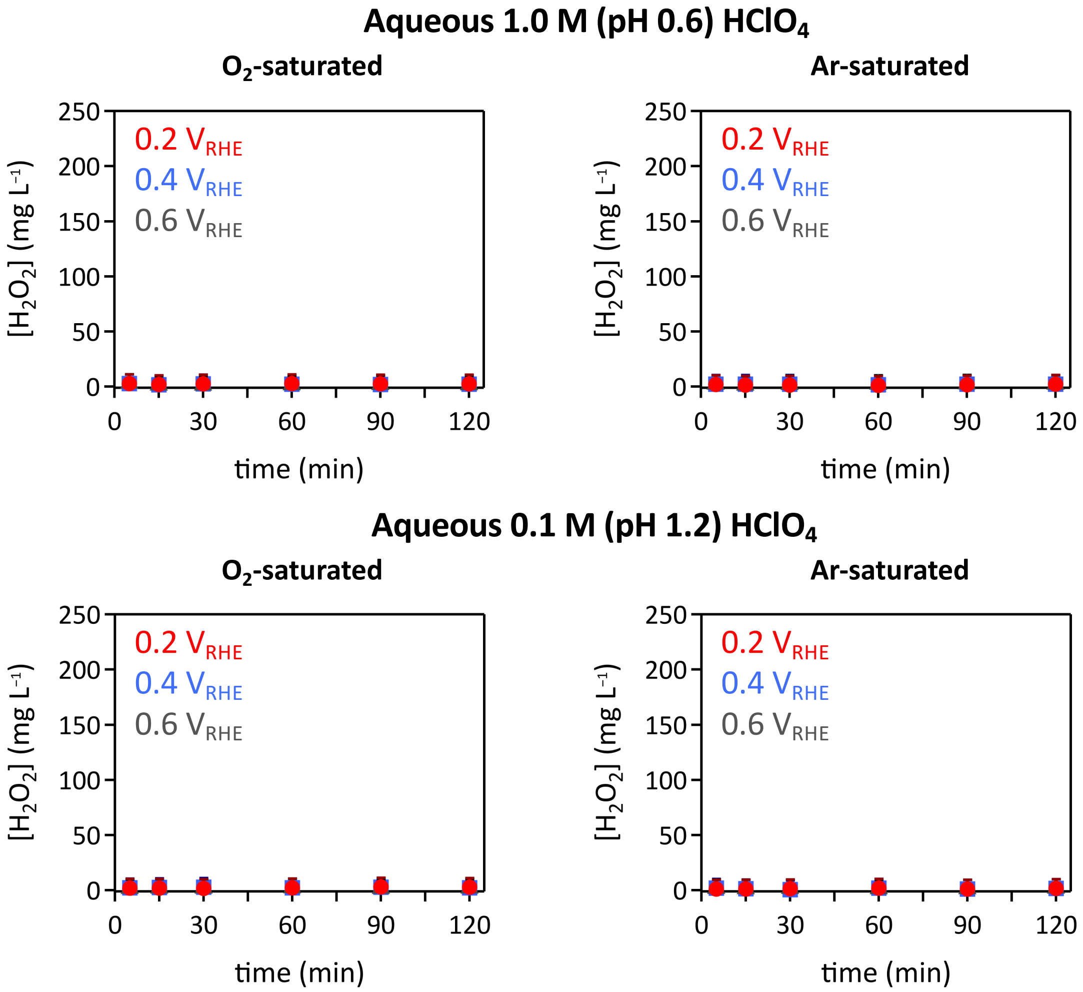


**Figure S3.** Concentrations of generated $H_2O_2$ as a function of electrocatalysis time for electrolytes with pH values of 0.6 and 1.2. Error in concentrations of generated $H_2O_2$ is a constant 8.4 mg $L^{-1}$.

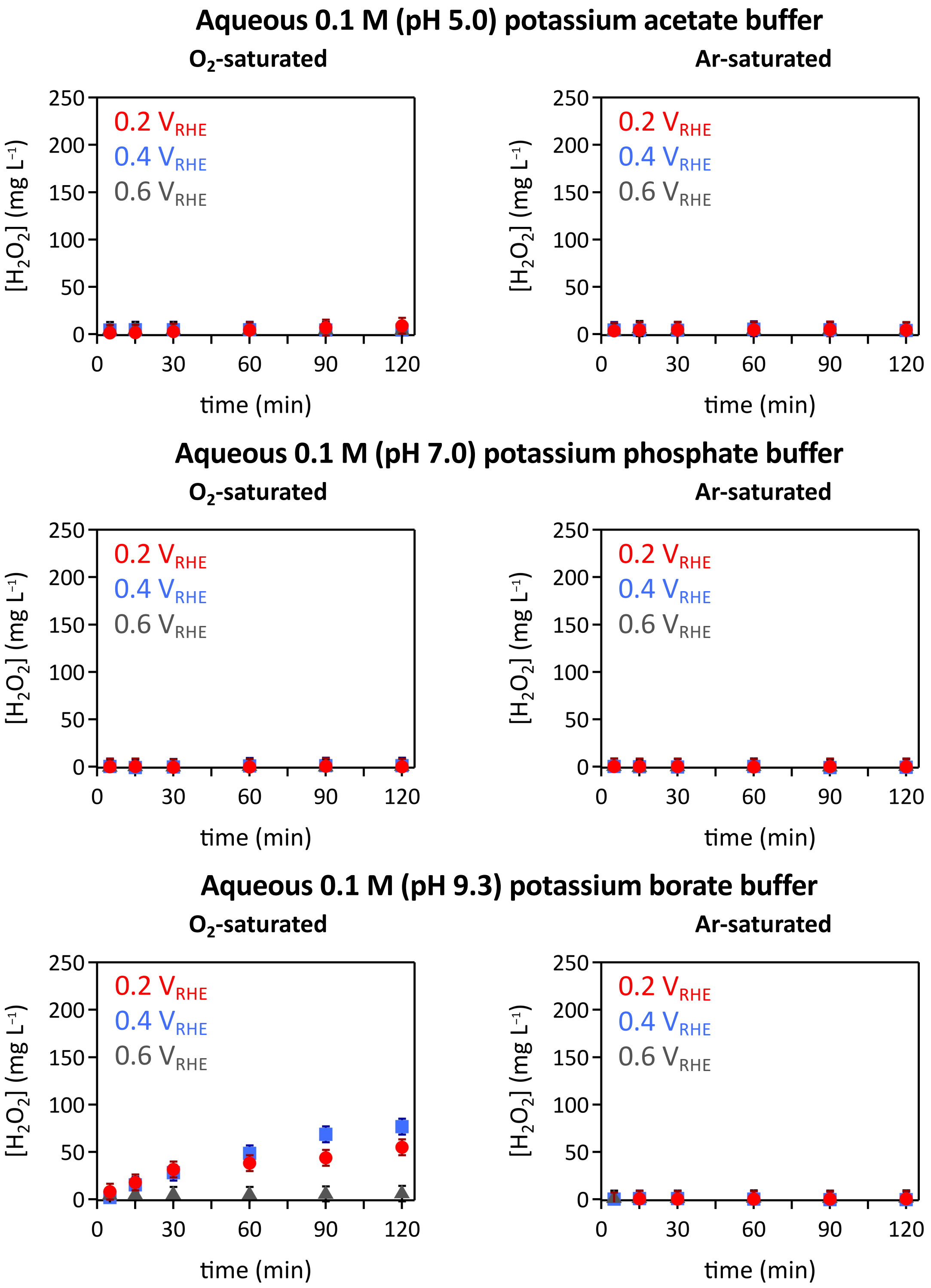


**Figure S4.** Concentrations of generated $H_2O_2$ as a function of electrocatalysis time for electrolytes with pH values of 5.0 to 9.3. Error in concentrations of generated $H_2O_2$ is a constant 8.4 mg $L^{-1}$.

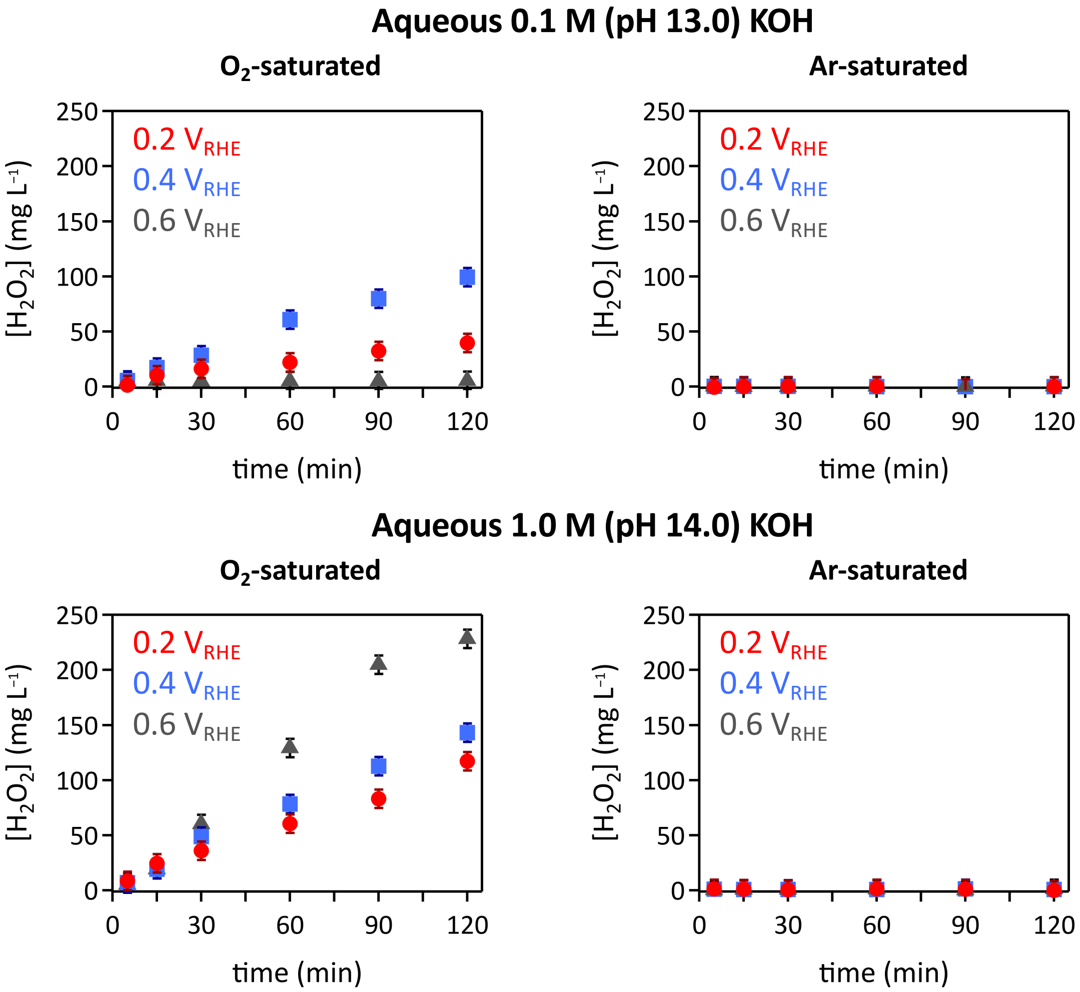


**Figure S5.** Concentrations of generated $H_2O_2$ as a function of electrocatalysis time for electrolytes with pH values of 13.0 and 14.0. Error in concentrations of generated $H_2O_2$ is a constant 8.4 mg $L^{-1}$.

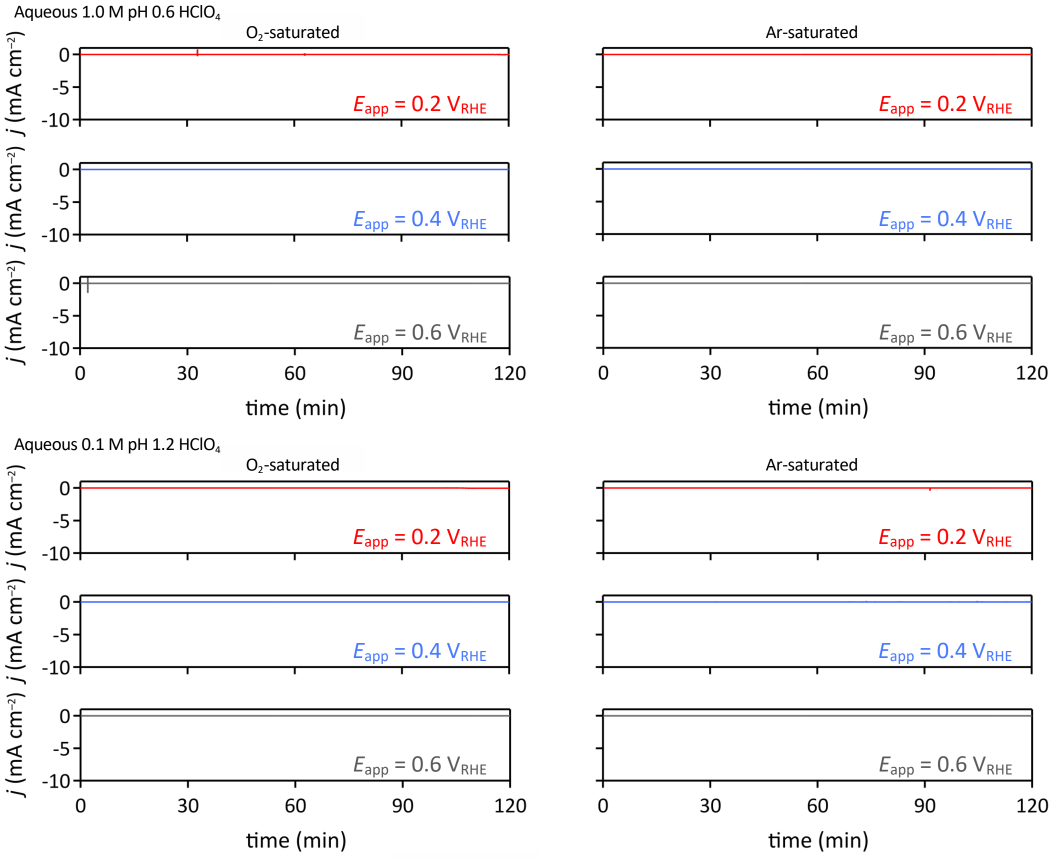


**Figure S6.** Chronoamperometry data for electrocatalysis at controlled applied potentials of 0.2, 0.4, or 0.6 V vs RHE in $O_2$- or Ar-saturated aqueous electrolytes with pH values of 0.6 and 1.2.

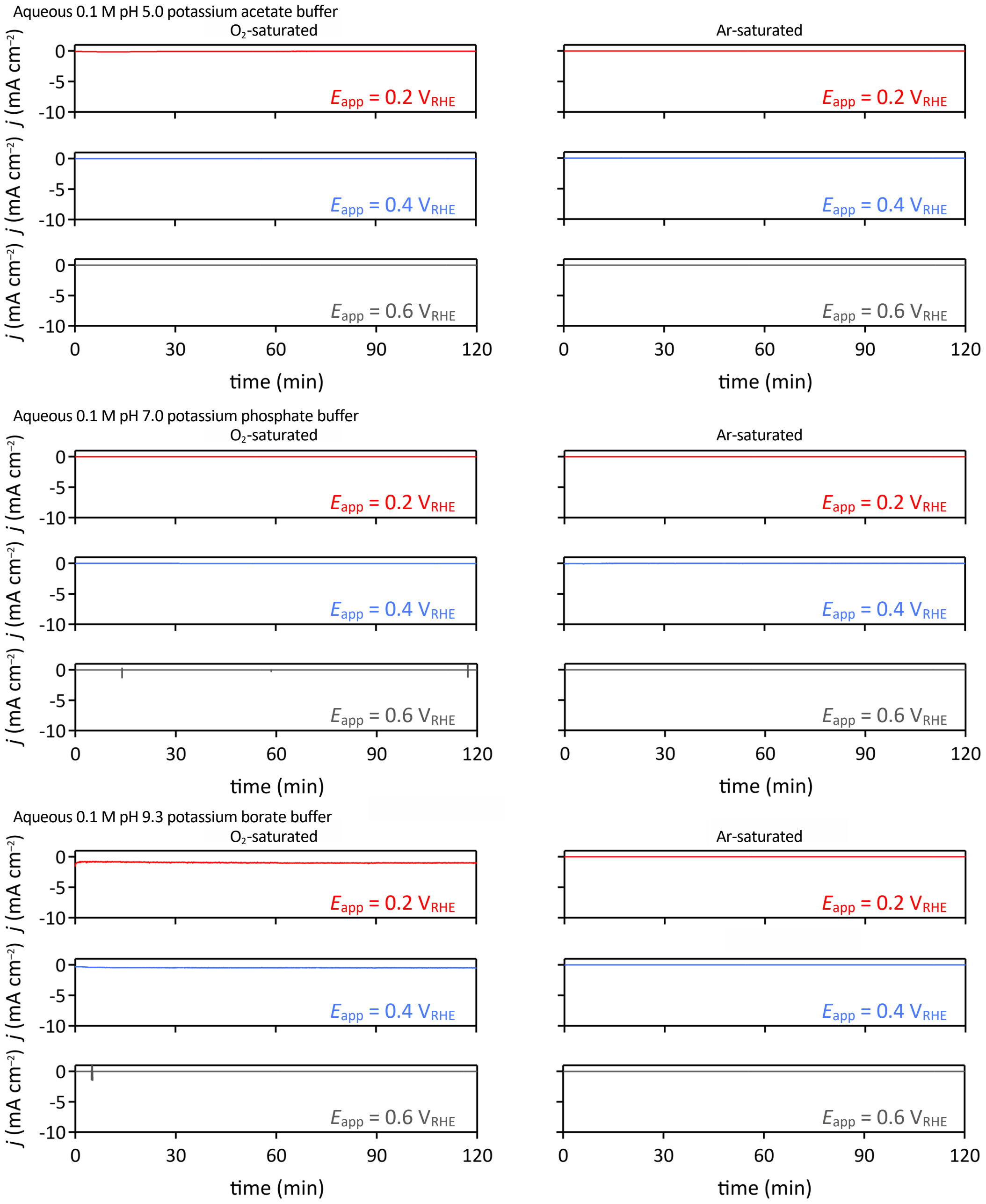


**Figure S7.** Chronoamperometry data for electrocatalysis at controlled applied potentials of 0.2, 0.4, or 0.6 V vs RHE in $O_2$- or Ar-saturated aqueous electrolytes with pH values from 5.0 to 9.3.

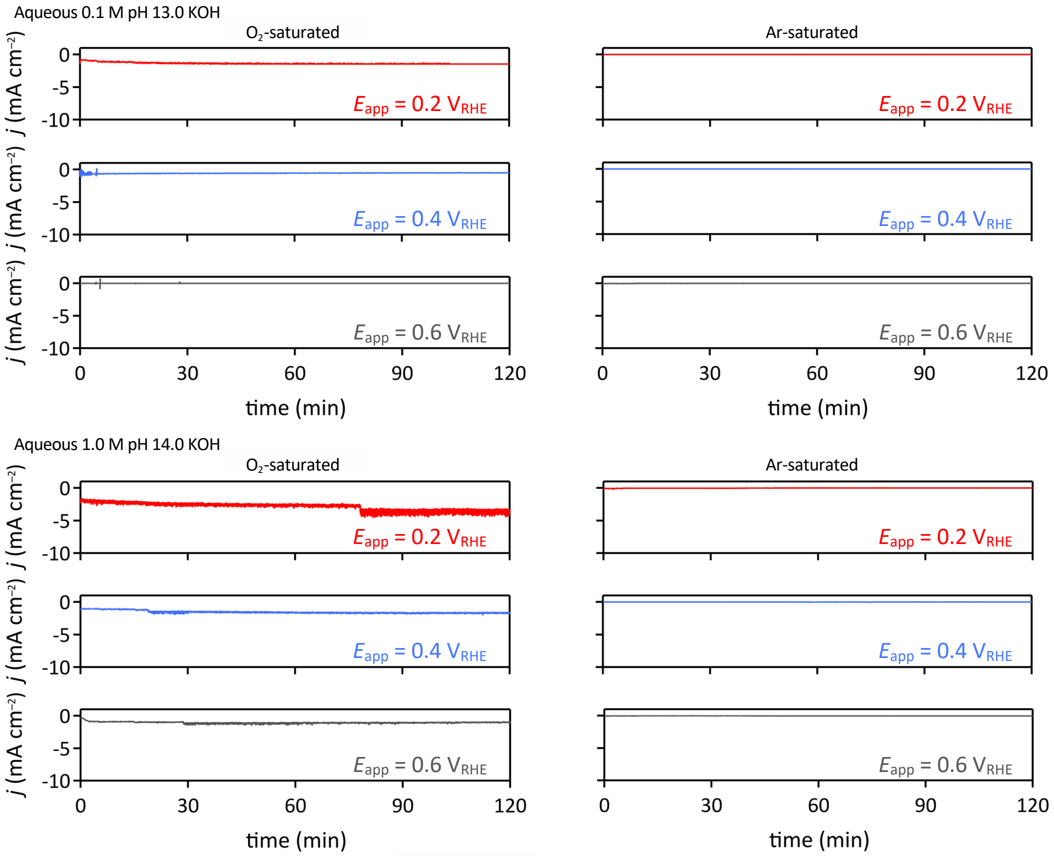


**Figure S8.** Chronoamperometry data for electrocatalysis at controlled applied potentials of 0.2, 0.4, or 0.6 V vs RHE in $O_2$- or Ar-saturated aqueous electrolytes with pH values of 13.0 and 14.0.

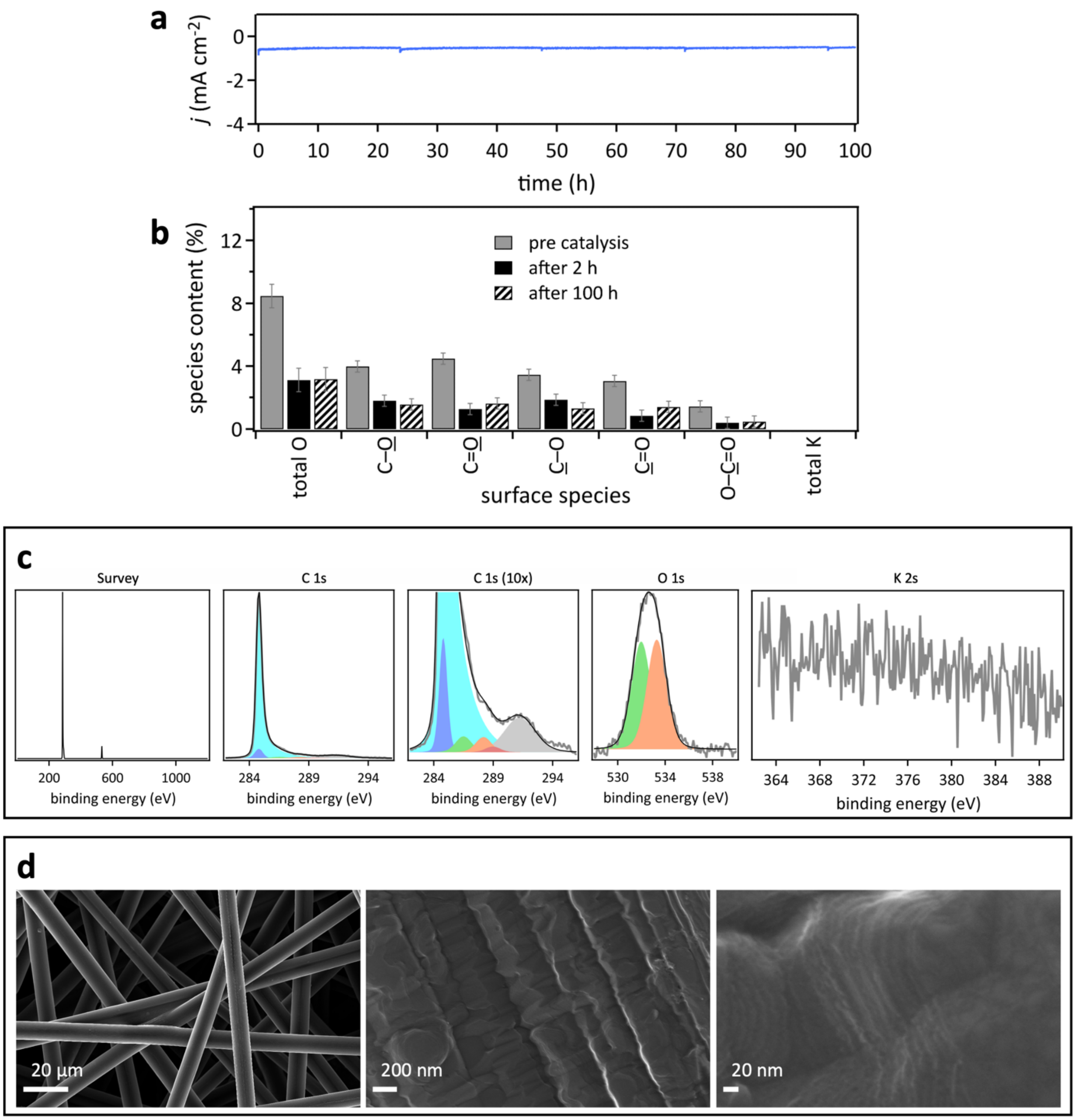


**Figure S9.** Long-term stability and characterization of hydrophilic carbon fiber paper during electrocatalysis in $O_2$-saturated 0.1 M pH 13.0 KOH electrolyte at 0.4 V vs RHE. (a) Chronoamperometry showing long-term stability. (b) Total carbon surface oxygenate contents from XPS before catalysis and after 2 or 100 h. (c) XPS spectra and peak fits after 100 h; peaks: graphitic carbon (cyan), adventitious carbon (lavender), shake-up (gray), C–O (orange), C=O (green), O=C–O (red). (d) SEM images after 100 h of electrocatalysis.

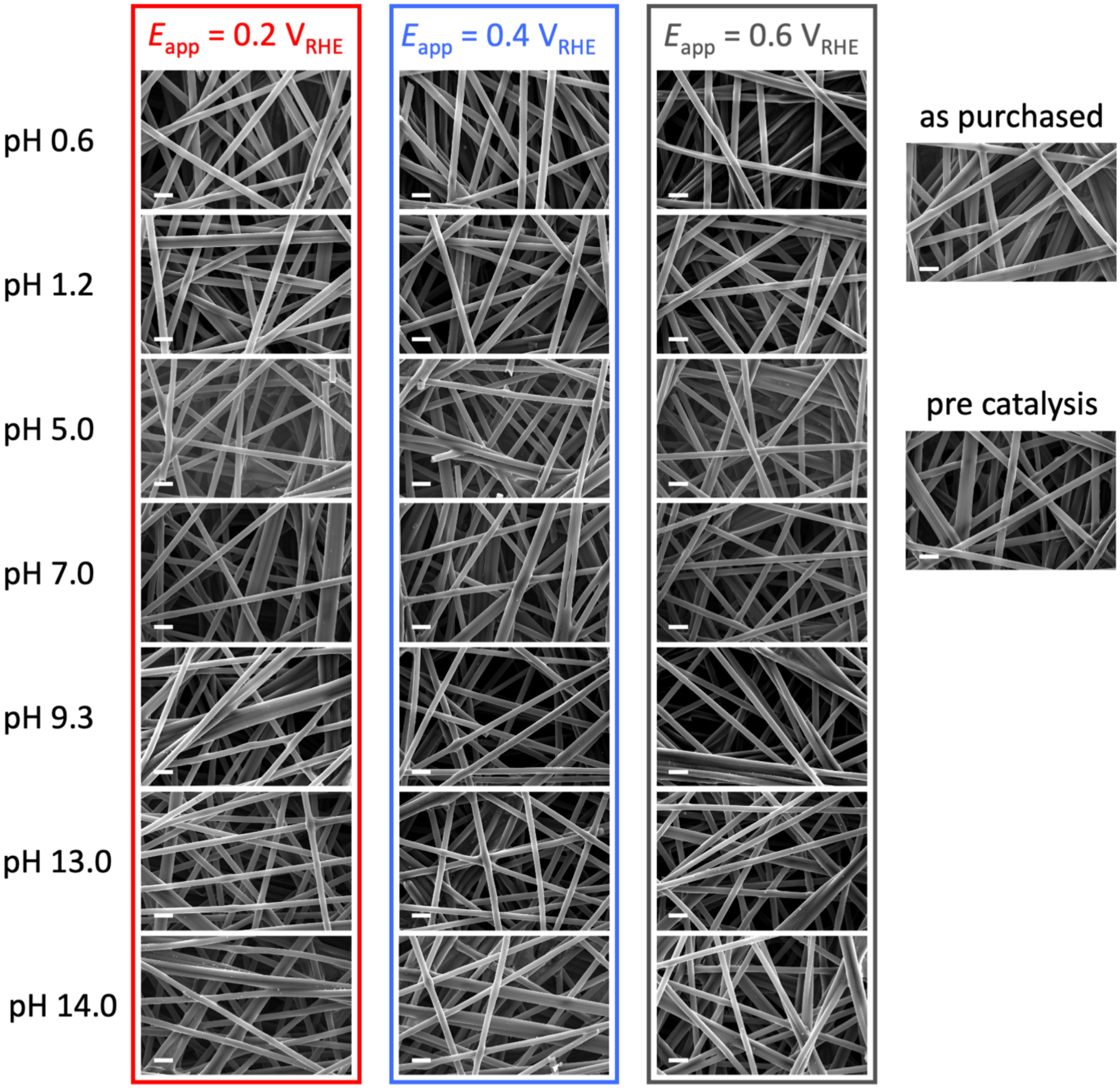


**Figure S10.** Structural integrity. SEM images of as-purchased hydrophobic carbon fiber paper, hydrophilic carbon fiber paper pre catalysis, and carbon fiber paper catalysts post ORR electrocatalysis as a function of electrolyte pH and applied potential. All scalebars are 20 μm.

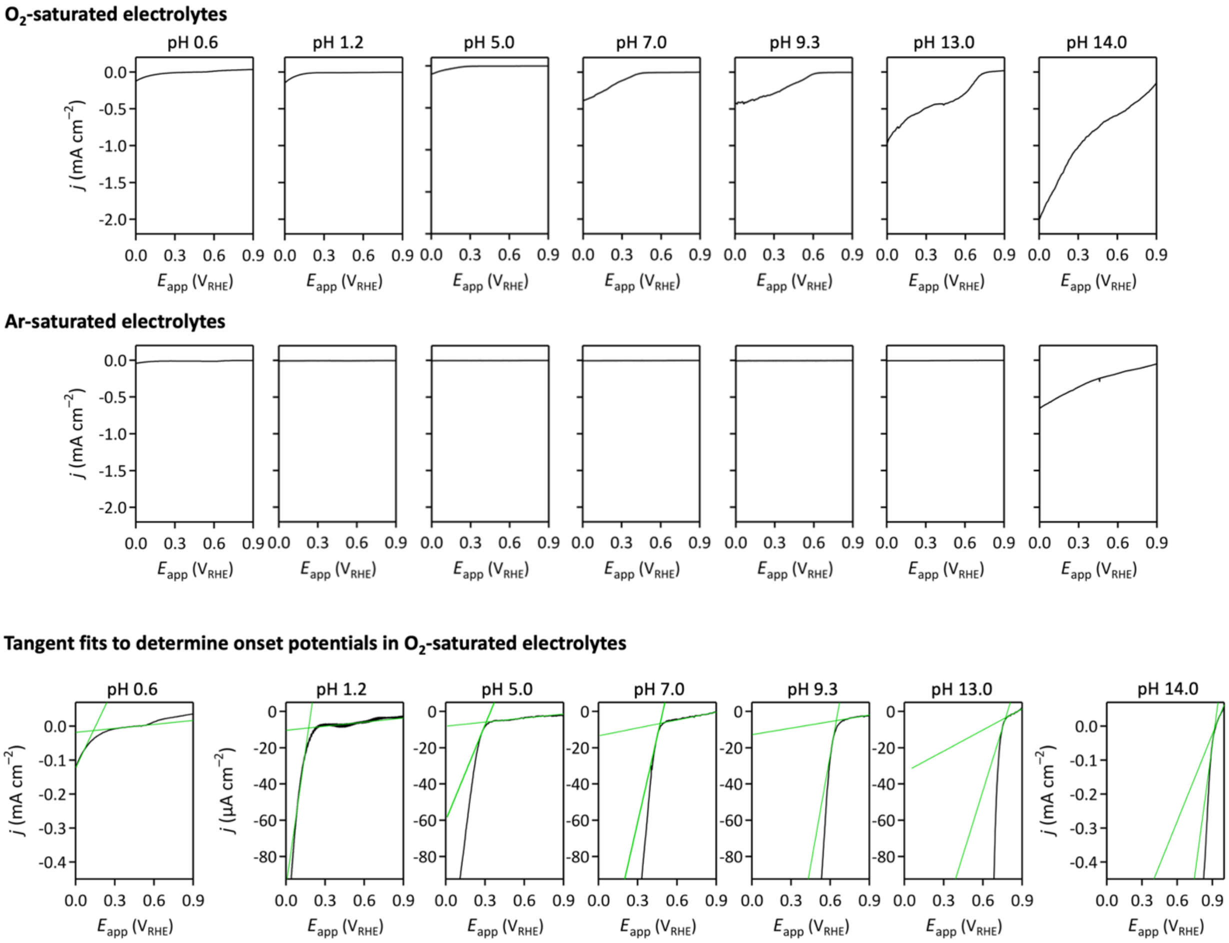


**Figure S11.** Linear sweep voltammograms for electrocatalysis in $O_2$- or Ar-saturated aqueous electrolytes with pH values ranging from 0.6 to 14.0, along with tangent fits (green lines).

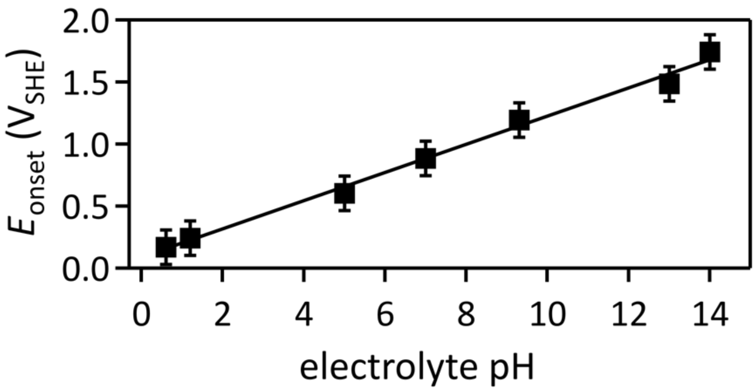


**Figure S12.** Onset potentials ($V_{SHE}$) for $H_2O_2$ generation as a function of electrolyte pH, obtained from ORR catalyzed by hydrophilic carbon fiber paper cathodes. Squares, data; line, linear fit.

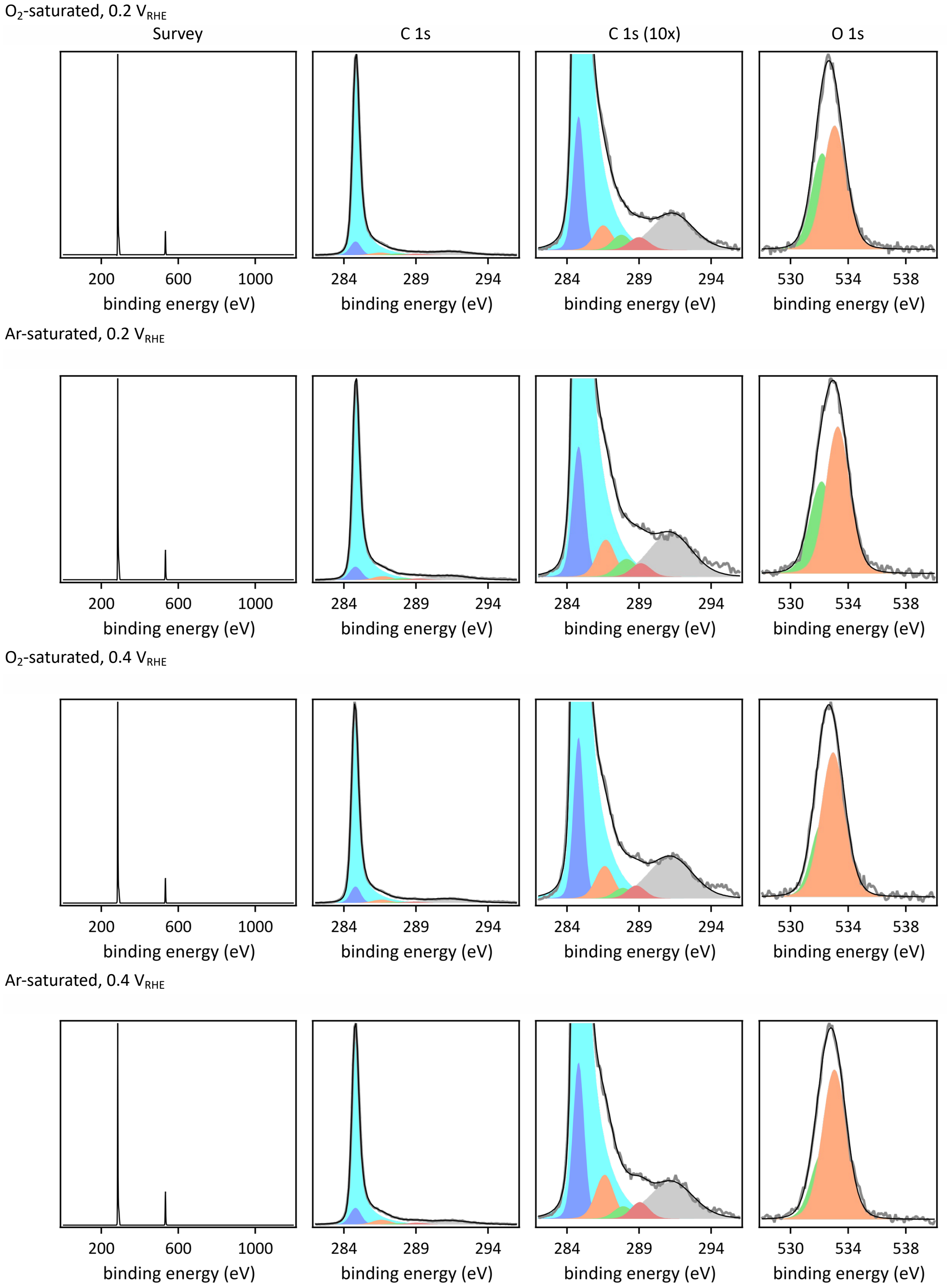


**Figure S13.** XPS data and peak fits of hydrophilic carbon fiber paper after 2 h of electrocatalysis in $O_2$- or Ar-saturated aqueous 1.0 M pH 0.6 perchloric acid electrolyte at 0.2 or 0.4 V vs RHE; peaks: graphitic carbon (cyan), adventitious carbon (lavender), shake-up peak (gray), C–O (orange), C=O (green), O=C–O (red).

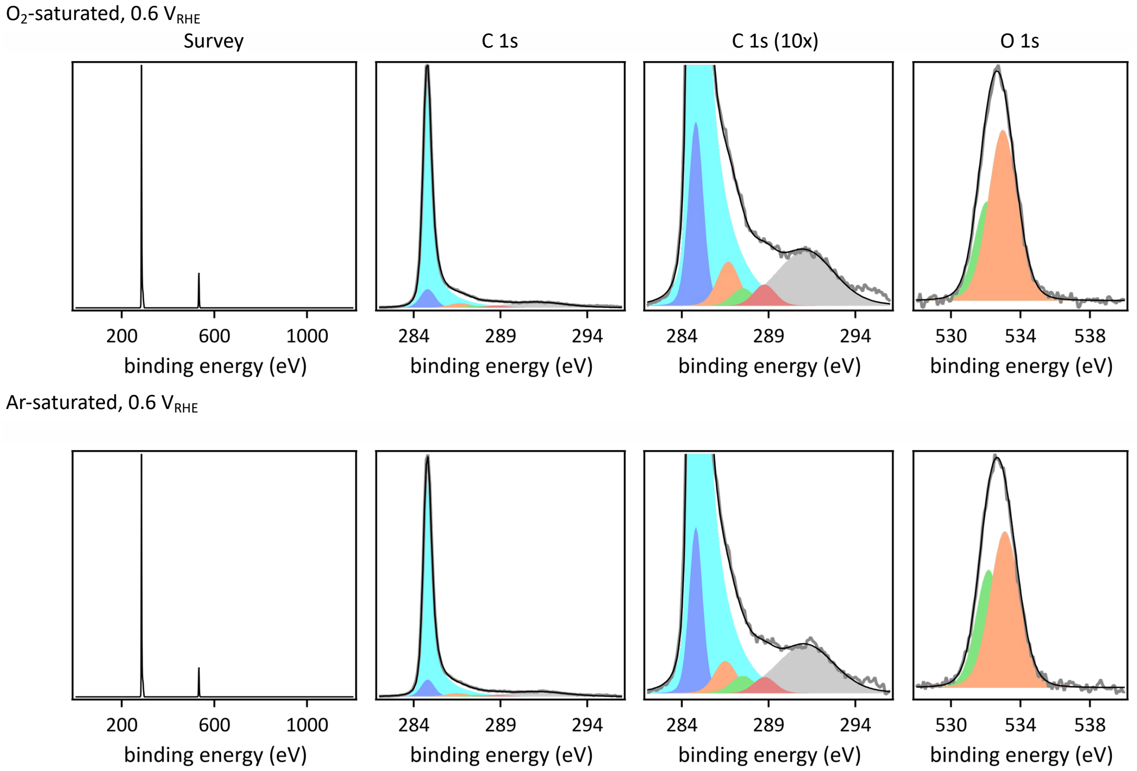


**Figure S14.** XPS data and peak fits of hydrophilic carbon fiber paper after 2 h of electrocatalysis in $O_2$- or Ar-saturated aqueous 1.0 M pH 0.6 perchloric acid electrolyte at 0.6 V vs RHE; peaks: graphitic carbon (cyan), adventitious carbon (lavender), shake-up peak (gray), C–O (orange), C=O (green), O=C–O (red).

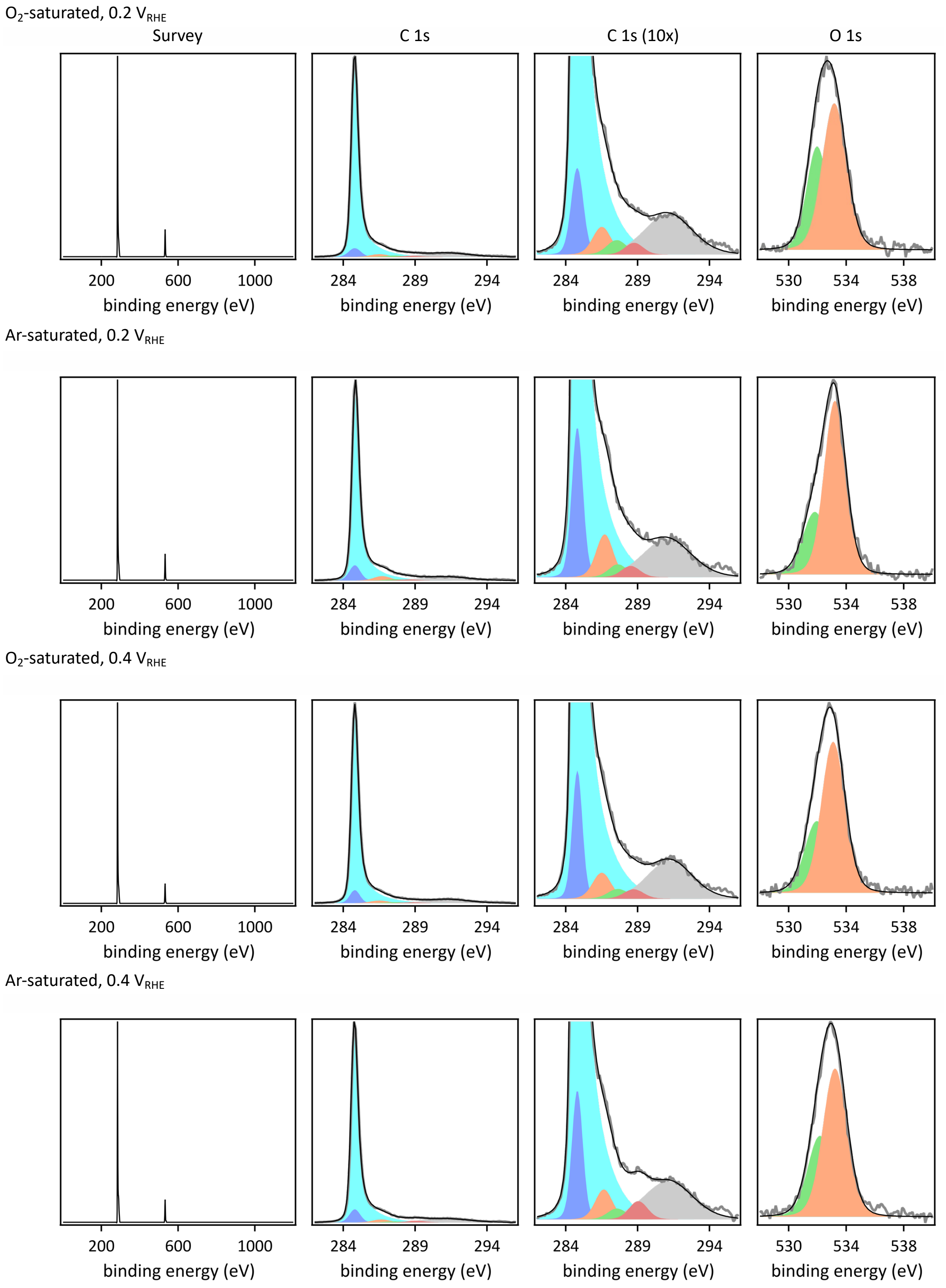


**Figure S15.** XPS data and peak fits of hydrophilic carbon fiber paper after 2 h of electrocatalysis in $O_2$- or Ar-saturated aqueous 0.1 M pH 1.2 perchloric acid electrolyte at 0.2 or 0.4 V vs RHE; peaks: graphitic carbon (cyan), adventitious carbon (lavender), shake-up peak (gray), C–O (orange), C=O (green), O=C–O (red).

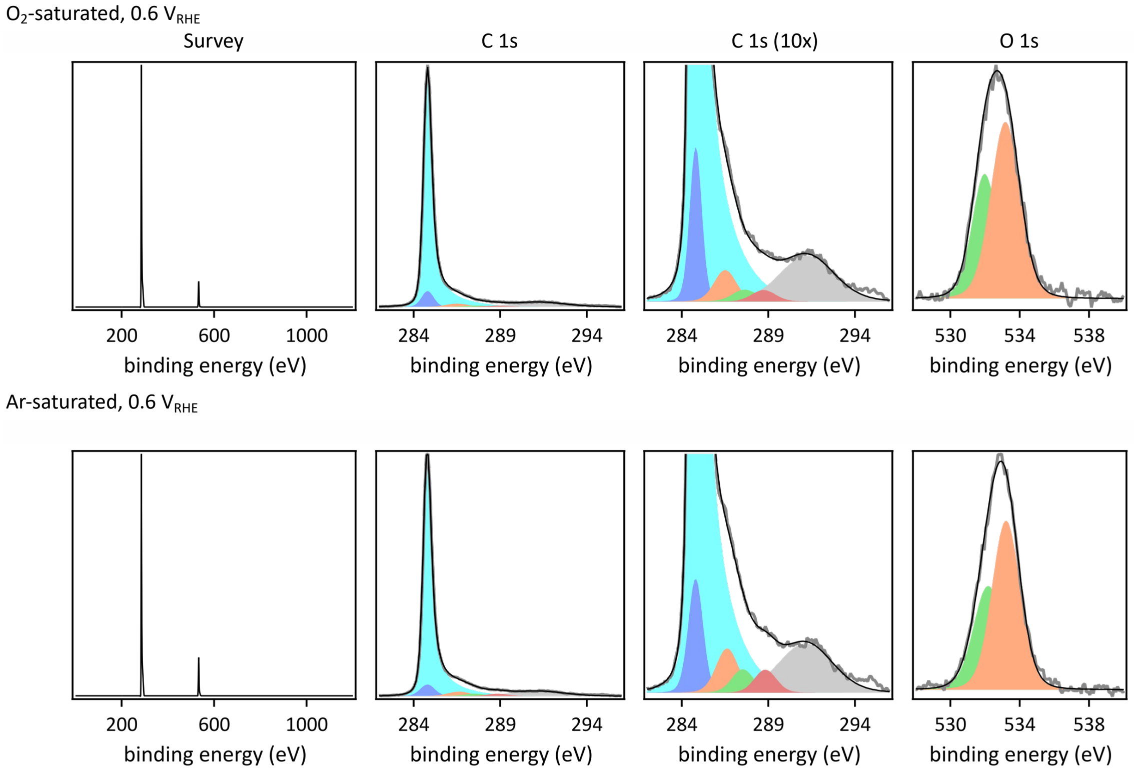


**Figure S16.** XPS data and peak fits of hydrophilic carbon fiber paper after 2 h of electrocatalysis in $O_2$- or Ar-saturated aqueous 0.1 M pH 1.2 perchloric acid electrolyte at 0.6 V vs RHE; peaks: graphitic carbon (cyan), adventitious carbon (lavender), shake-up peak (gray), C–O (orange), C=O (green), O=C–O (red).

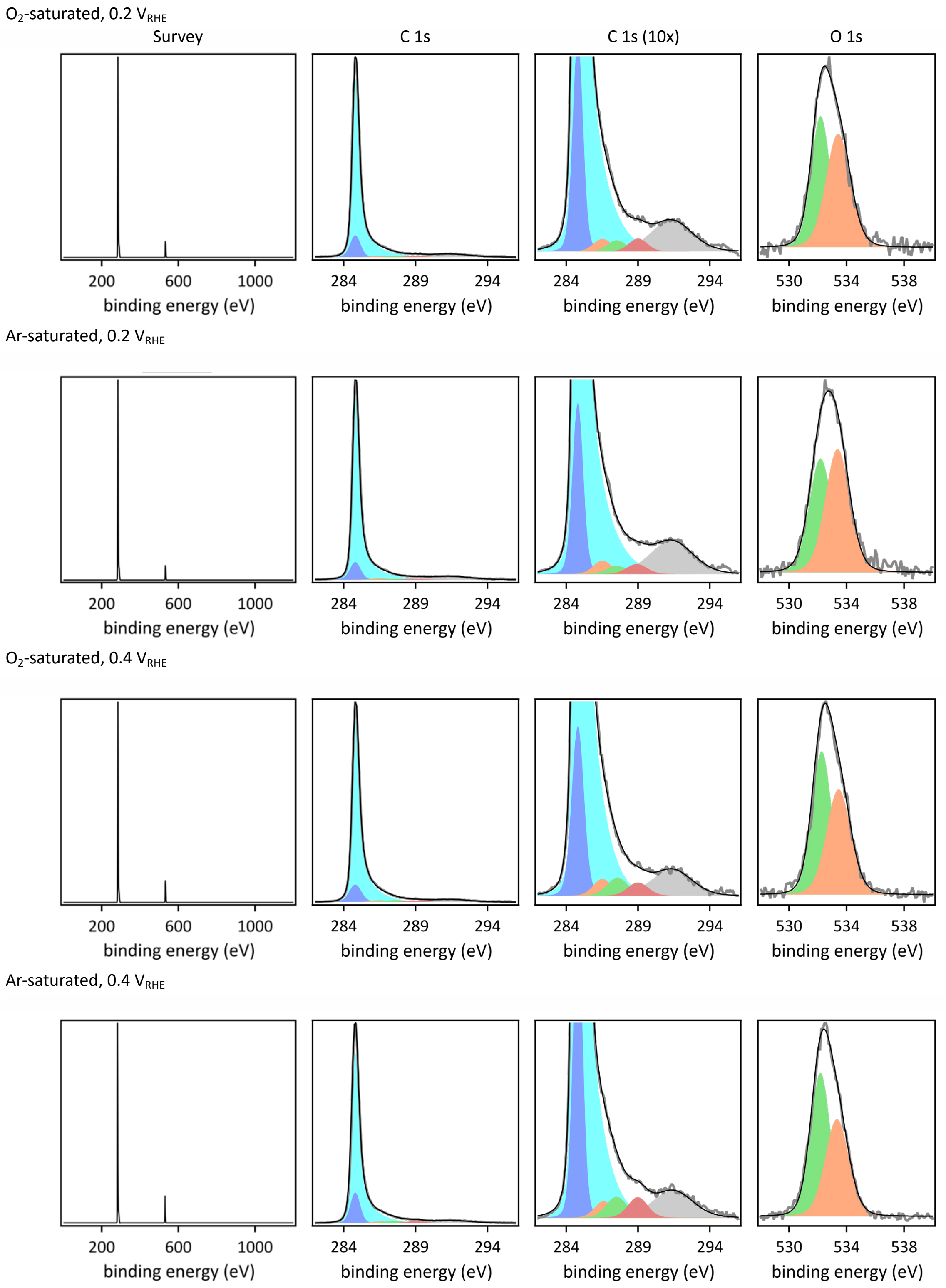


**Figure S17.** XPS data and peak fits of hydrophilic carbon fiber paper after 2 h of electrocatalysis in $O_2$- or Ar-saturated aqueous 0.1 M pH 5.0 potassium acetate buffer electrolyte at 0.2 or 0.4 V vs RHE; peaks: graphitic carbon (cyan), adventitious carbon (lavender), shake-up peak (gray), C–O (orange), C=O (green), O=C–O (red).

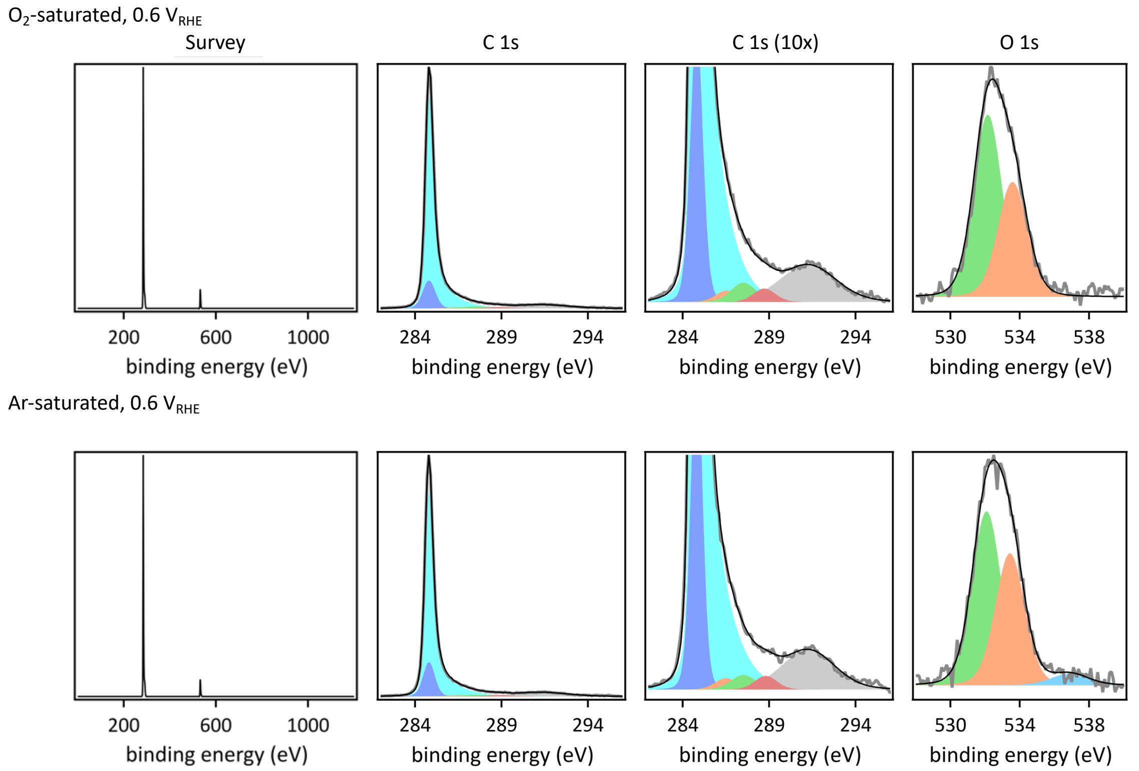


**Figure S18.** XPS data and peak fits of hydrophilic carbon fiber paper after 2 h of electrocatalysis in $O_2$- or Ar-saturated aqueous 0.1 M pH 5.0 potassium acetate buffer electrolyte at 0.6 V vs RHE; peaks: graphitic carbon (cyan), adventitious carbon (lavender), shake-up peak (gray), C–O (orange), C=O (green), O=C–O (red).

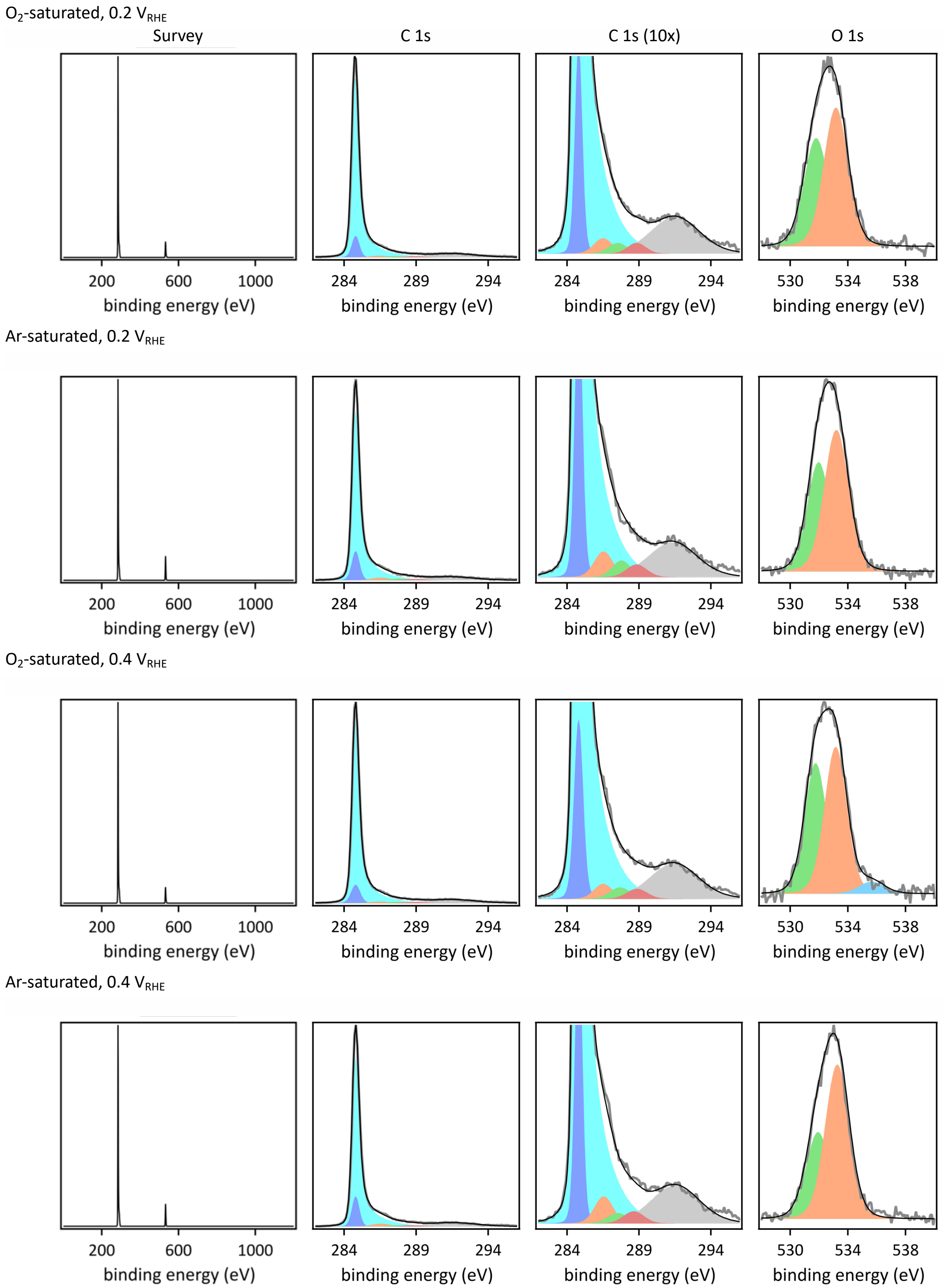


**Figure S19.** XPS data and peak fits of hydrophilic carbon fiber paper after 2 h of electrocatalysis in $O_2$- or Ar-saturated aqueous 0.1 M pH 7.0 potassium phosphate buffer electrolyte at 0.2 or 0.4 V vs RHE; peaks: graphitic carbon (cyan), adventitious carbon (lavender), shake-up peak (gray), C–O (orange), C=O (green), O=C–O (red).

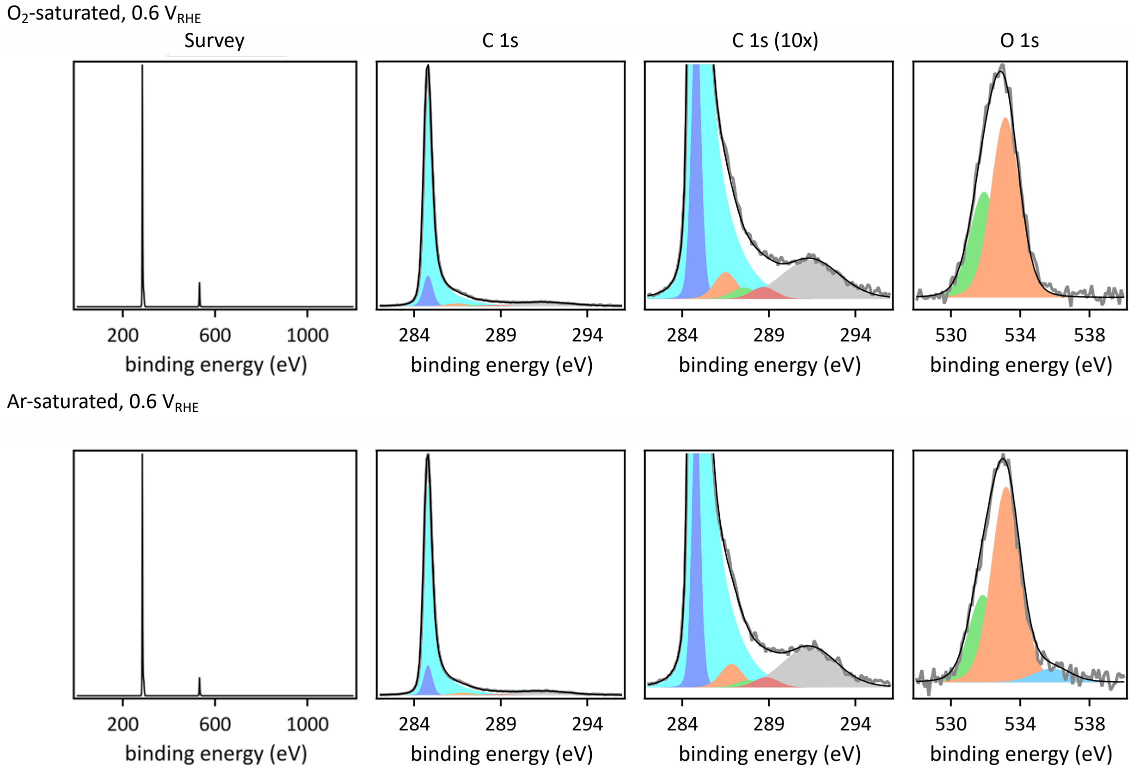


**Figure S20.** XPS data and peak fits of hydrophilic carbon fiber paper after 2 h of electrocatalysis in $O_2$- or Ar-saturated aqueous 0.1 M pH 7.0 potassium phosphate buffer electrolyte at 0.6 V vs RHE; peaks: graphitic carbon (cyan), adventitious carbon (lavender), shake-up peak (gray), C–O (orange), C=O (green), O=C–O (red).

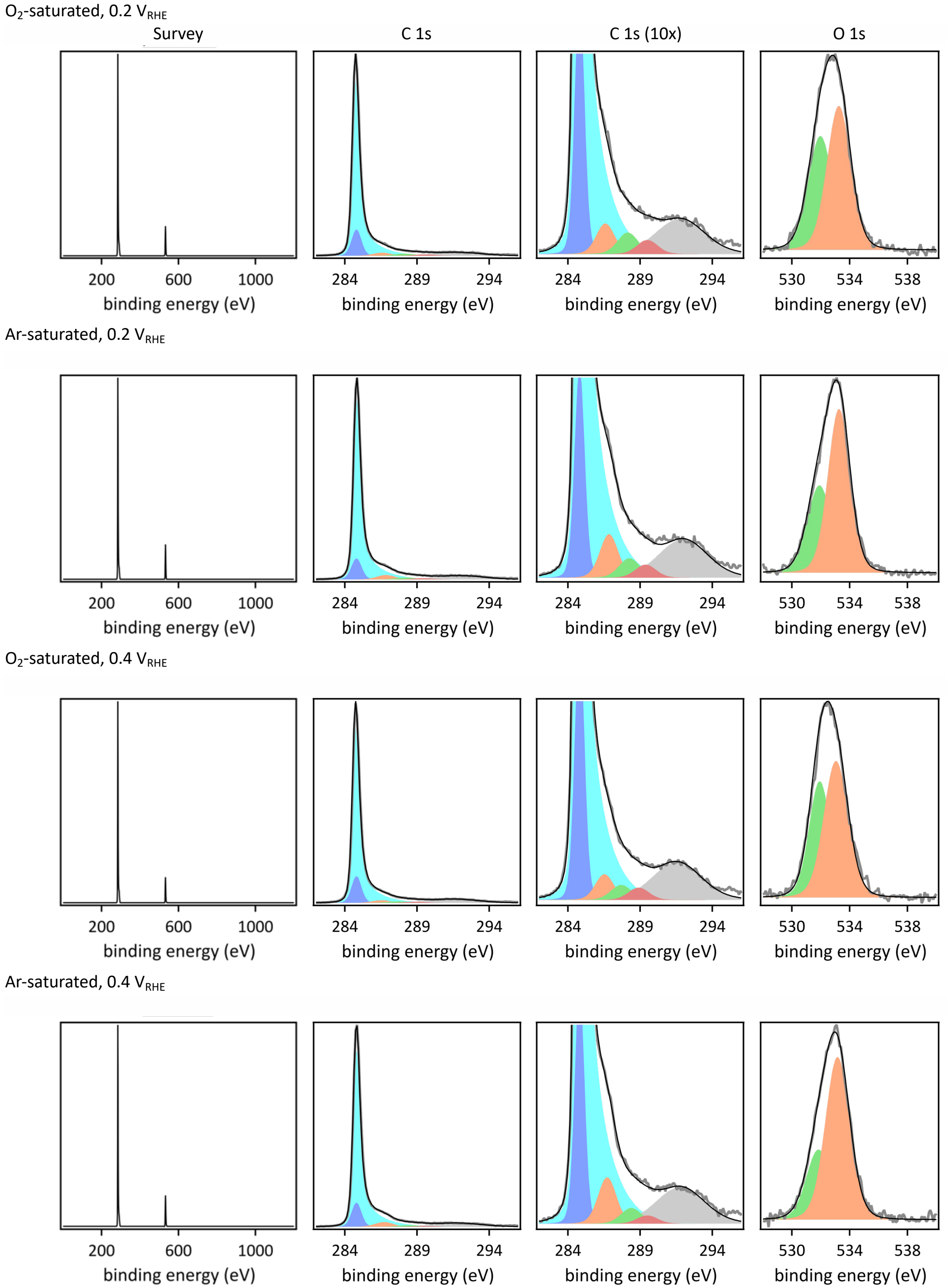


**Figure S21.** XPS data and peak fits of hydrophilic carbon fiber paper after 2 h of electrocatalysis in $O_2$- or Ar-saturated aqueous 0.1 M pH 9.3 potassium borate buffer electrolyte at 0.2 or 0.4 V vs RHE; peaks: graphitic carbon (cyan), adventitious carbon (lavender), shake-up peak (gray), C–O (orange), C=O (green), O=C–O (red).

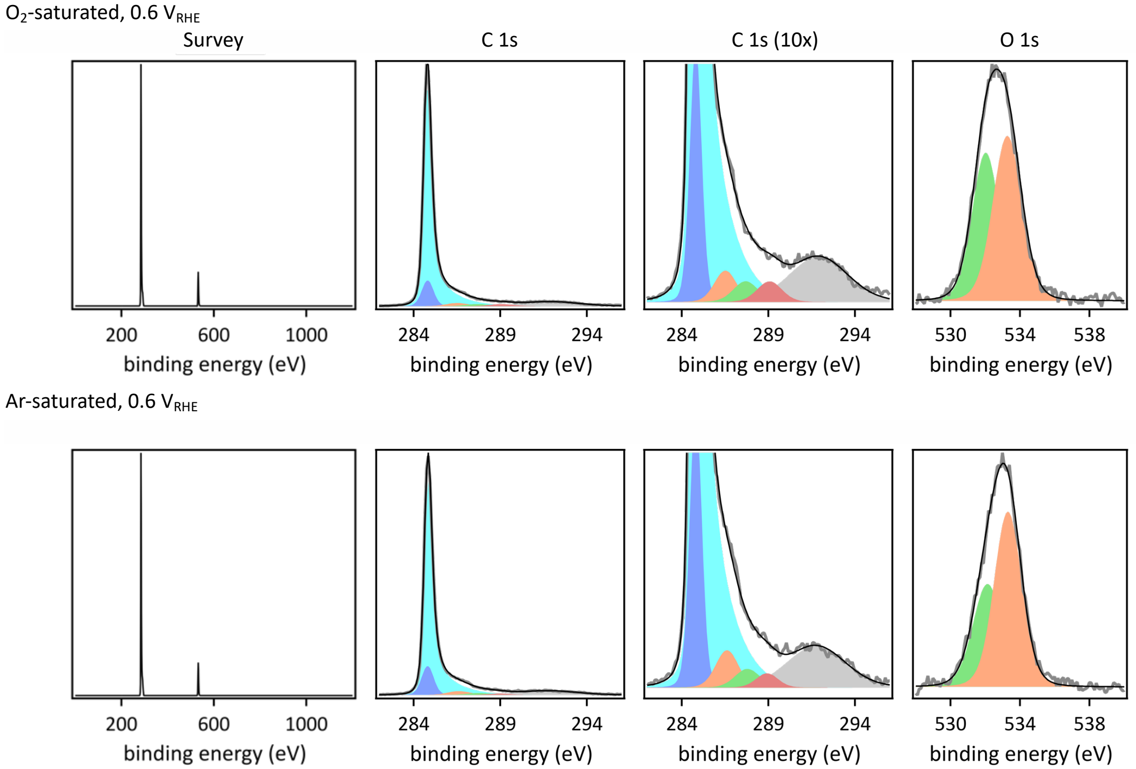


**Figure S22.** XPS data and peak fits of hydrophilic carbon fiber paper after 2 h of electrocatalysis in $O_2$- or Ar-saturated aqueous 0.1 M pH 9.3 potassium borate buffer electrolyte at 0.6 V vs RHE; peaks: graphitic carbon (cyan), adventitious carbon (lavender), shake-up peak (gray), C–O (orange), C=O (green), O=C–O (red).

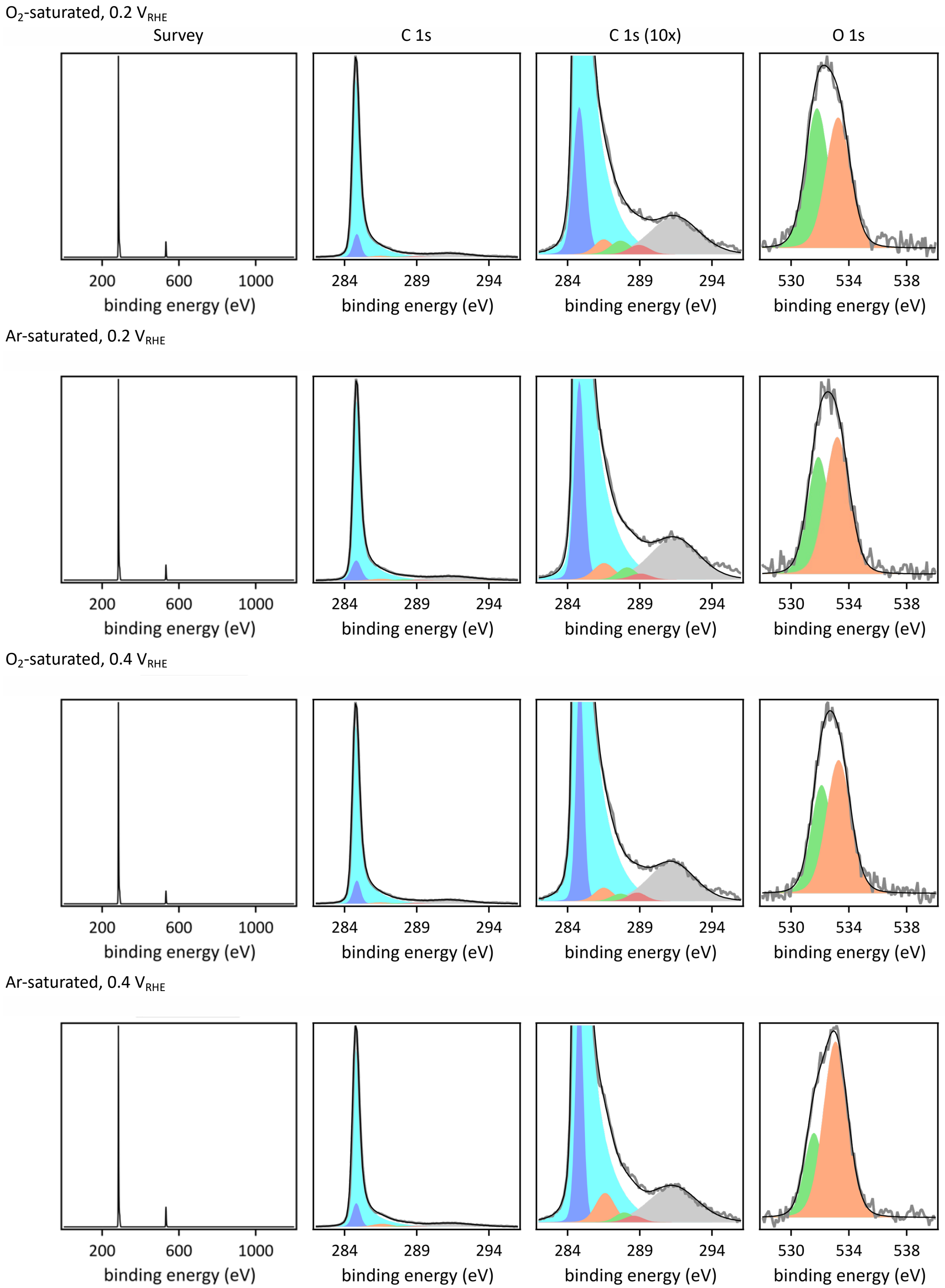


**Figure S23.** XPS data and peak fits of hydrophilic carbon fiber paper after 2 h of electrocatalysis in $O_2$- or Ar-saturated aqueous 0.1 M pH 13.0 potassium hydroxide electrolyte at 0.2 or 0.4 V vs RHE; peaks: graphitic carbon (cyan), adventitious carbon (lavender), shake-up peak (gray), C–O (orange), C=O (green), O=C–O (red).

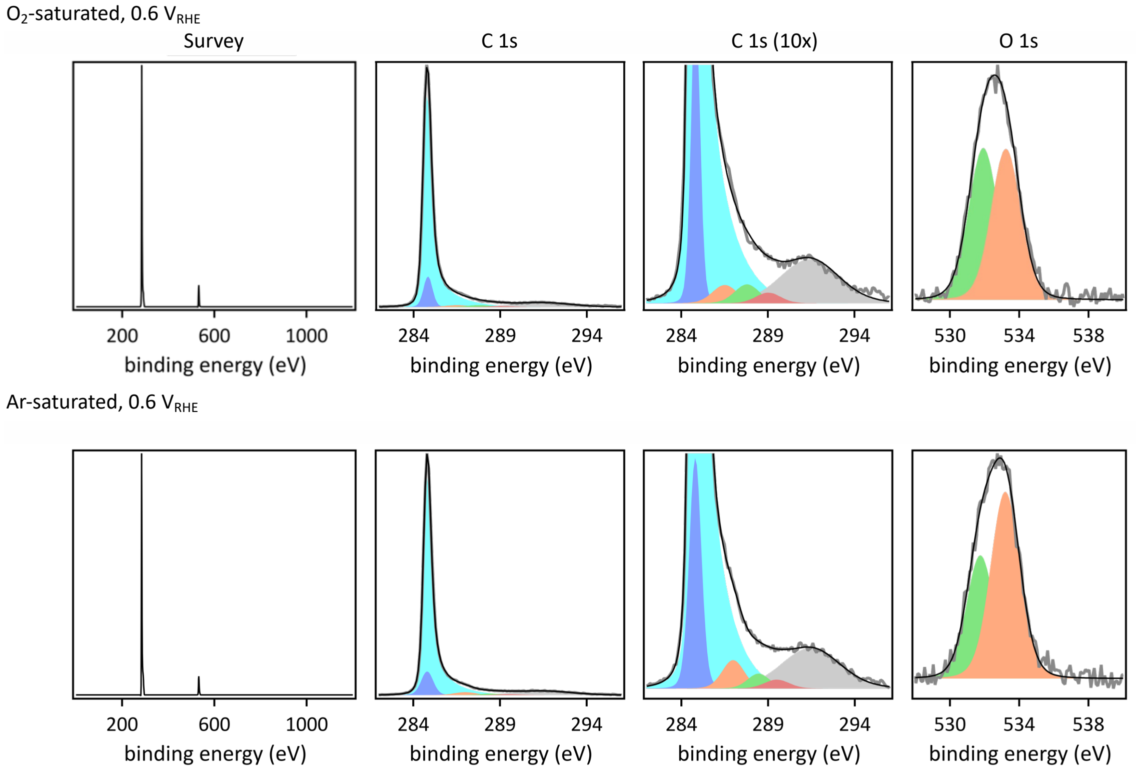


**Figure S24.** XPS data and peak fits of hydrophilic carbon fiber paper after 2 h of electrocatalysis in $O_2$- or Ar-saturated aqueous 0.1 M pH 13.0 potassium hydroxide electrolyte at 0.6 V vs RHE; peaks: graphitic carbon (cyan), adventitious carbon (lavender), shake-up peak (gray), C–O (orange), C=O (green), O=C–O (red).

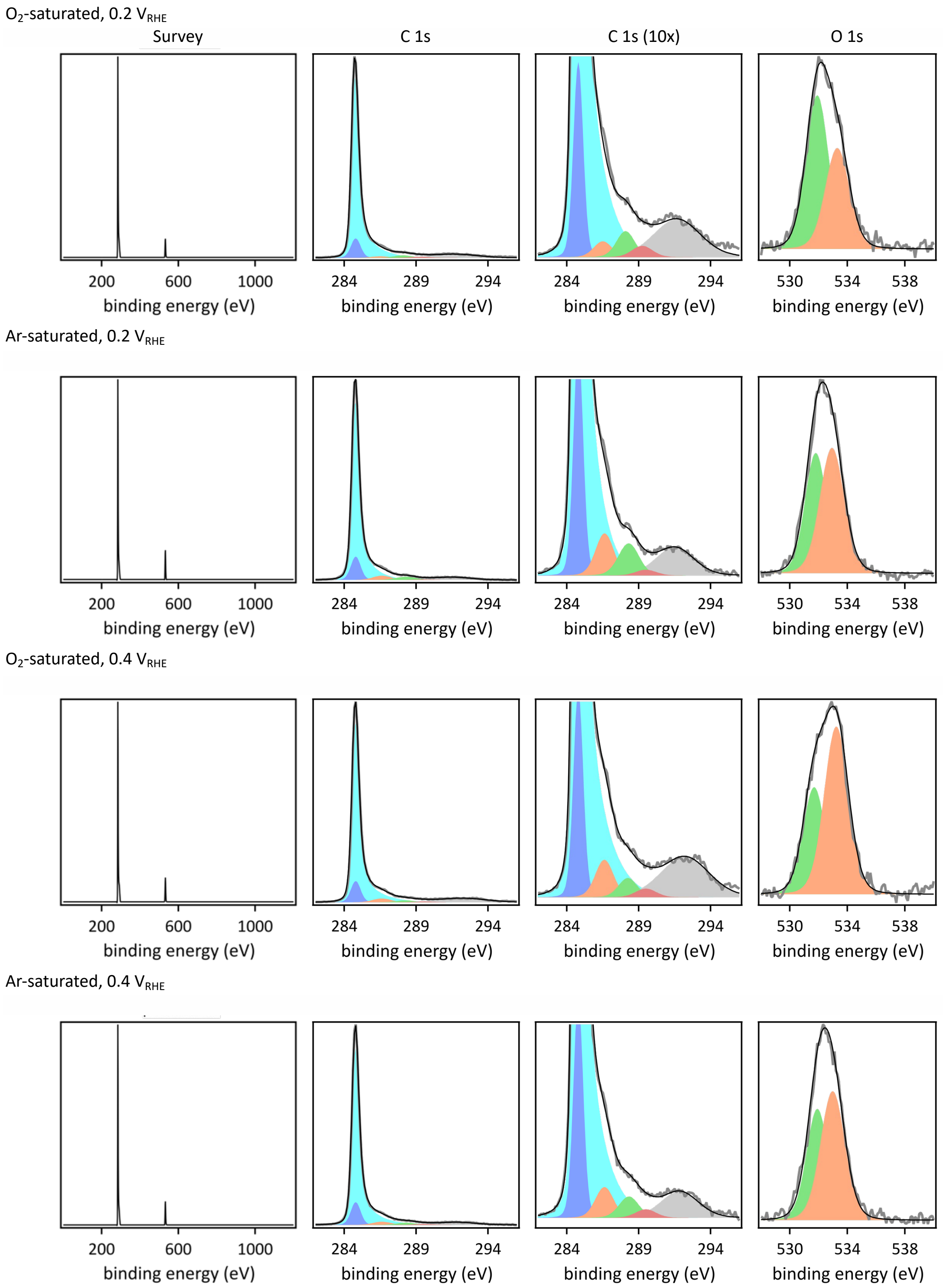


**Figure S25.** XPS data and peak fits of hydrophilic carbon fiber paper after 2 h of electrocatalysis in $O_2$- or Ar-saturated aqueous 1.0 M pH 14.0 potassium hydroxide electrolyte at 0.2 or 0.4 V vs RHE; peaks: graphitic carbon (cyan), adventitious carbon (lavender), shake-up peak (gray), C–O (orange), C=O (green), O=C–O (red).

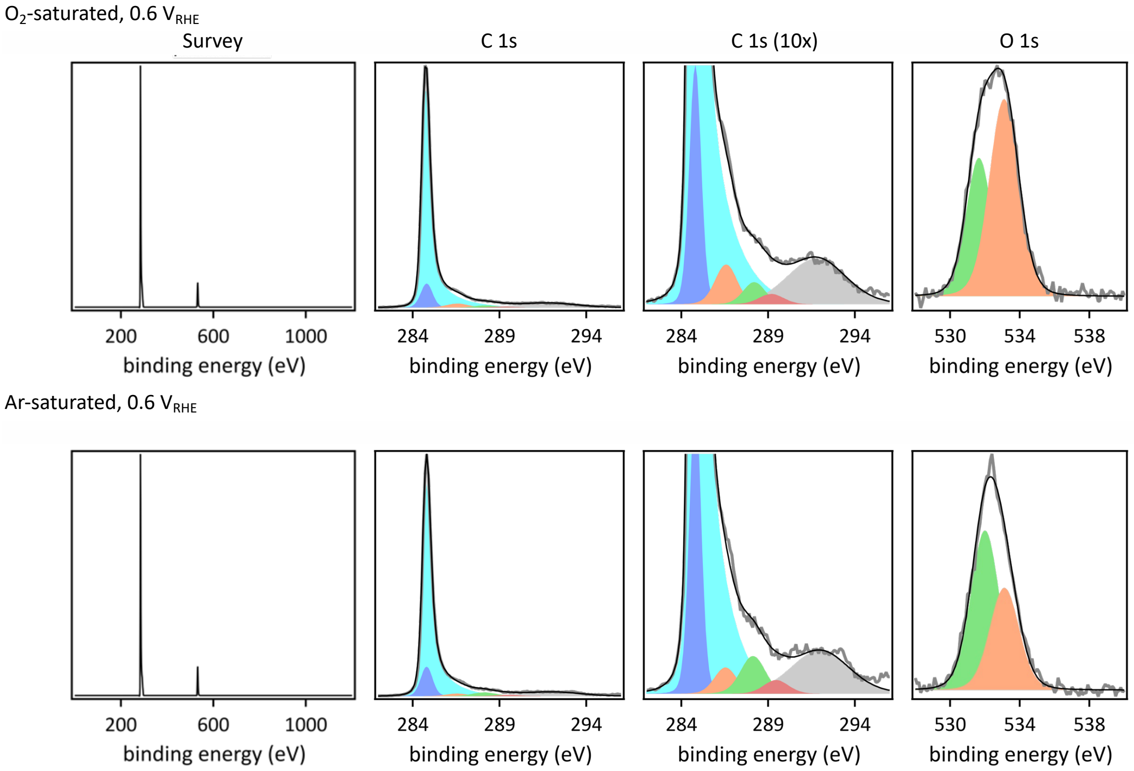


**Figure S26.** XPS data and peak fits of hydrophilic carbon fiber paper after 2 h of electrocatalysis in $O_2$- or Ar-saturated aqueous 1.0 M pH 14.0 potassium hydroxide electrolyte at 0.6 V vs RHE; peaks: graphitic carbon (cyan), adventitious carbon (lavender), shake-up peak (gray), C–O (orange), C=O (green), O=C–O (red).

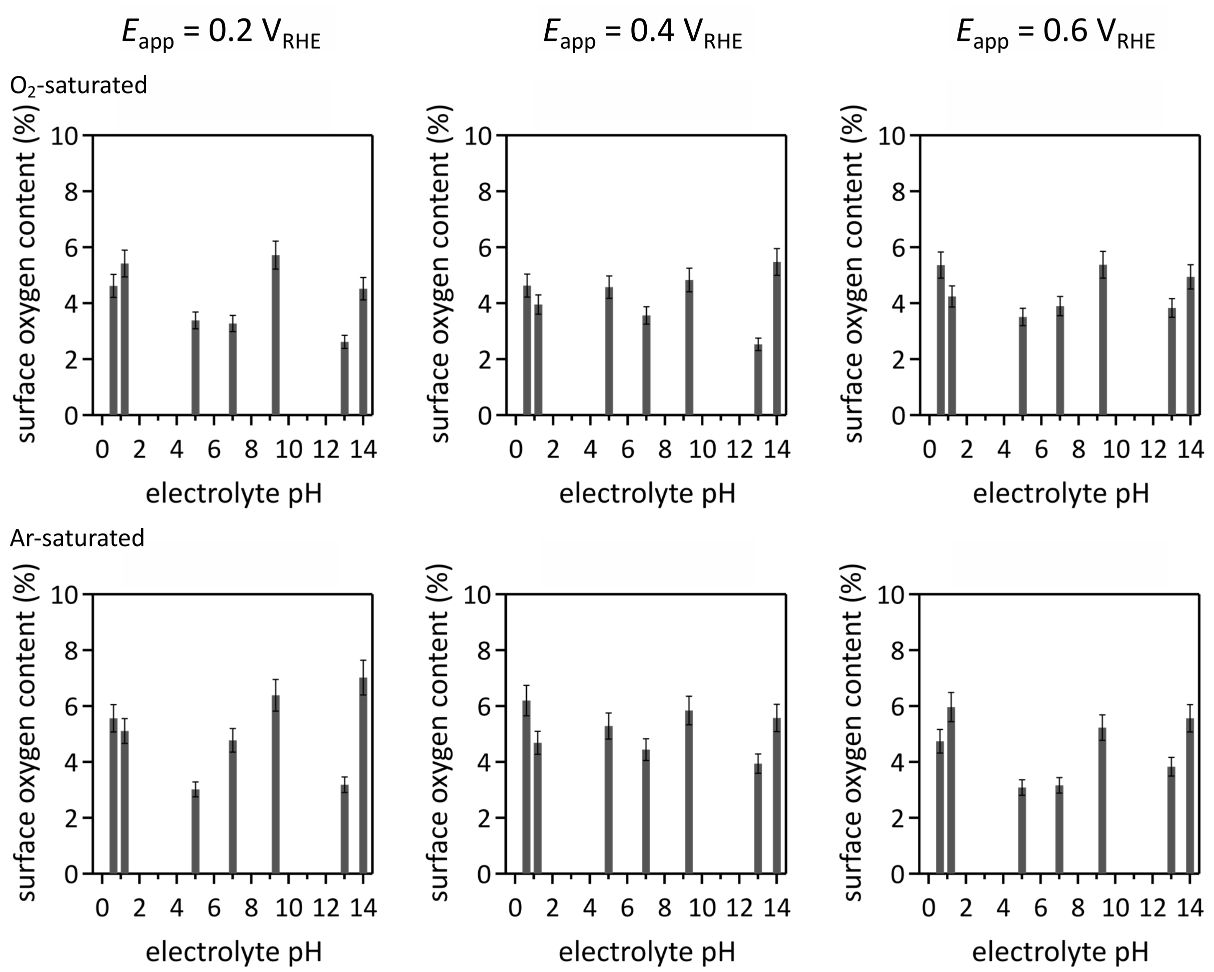


**Figure S27.** Total surface oxygen content on hydrophilic carbon fiber paper post catalysis, derived from peak fits of XPS data.

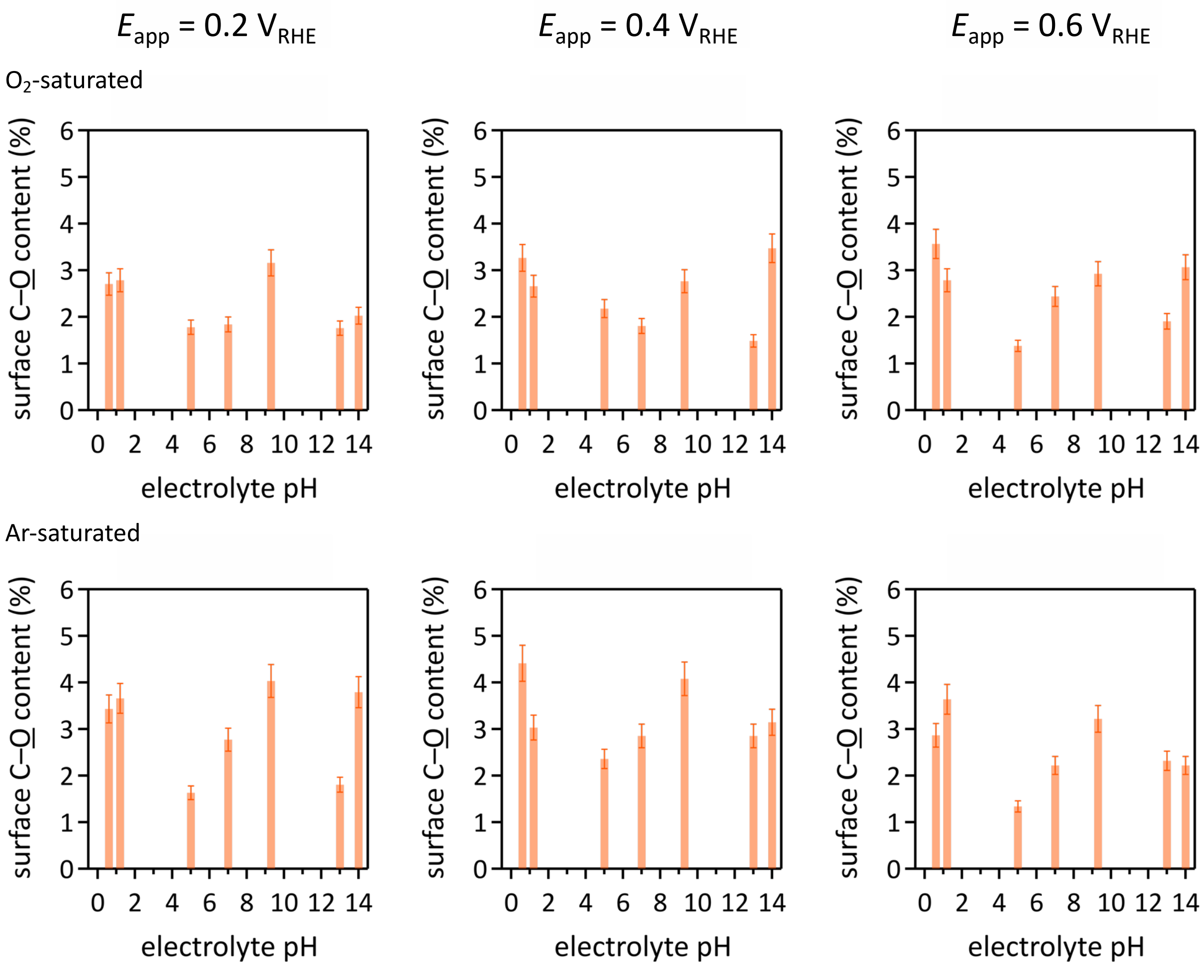


**Figure S28.** Surface C–O content on hydrophilic carbon fiber paper post catalysis, derived from peak fits of XPS data.

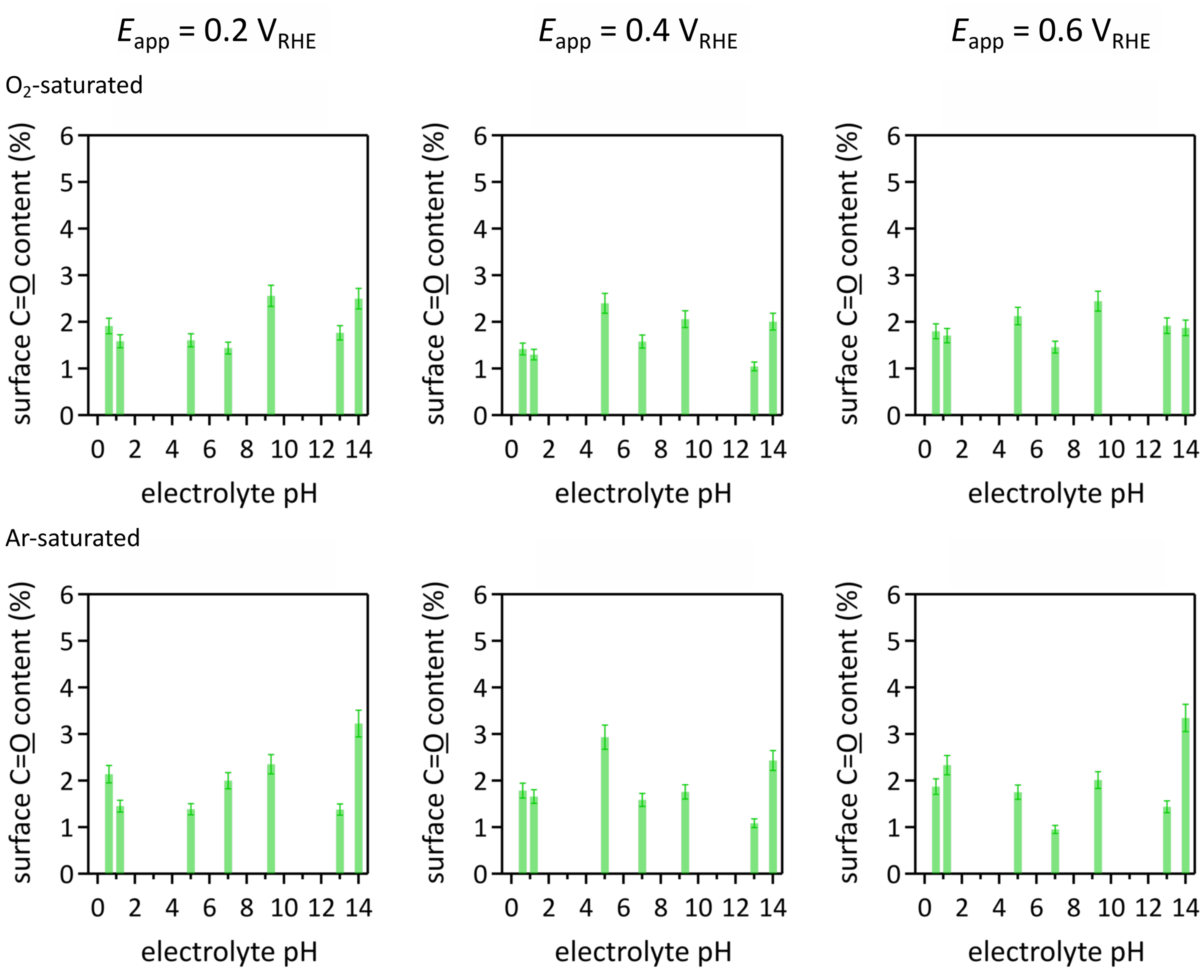


**Figure S29.** Surface C=O content on hydrophilic carbon fiber paper post catalysis, derived from peak fits of XPS data.

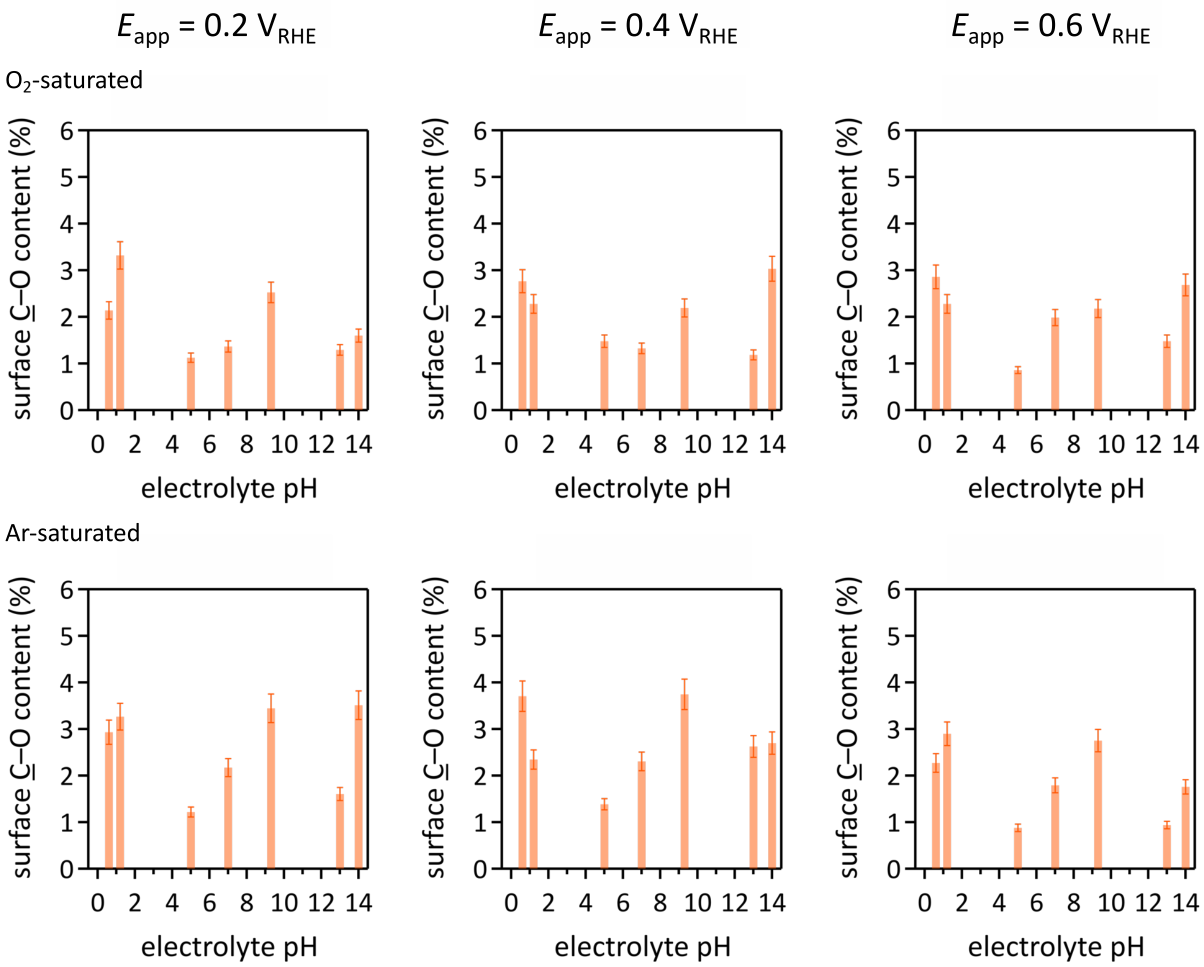


**Figure S30.** Surface C–O content on hydrophilic carbon fiber paper post catalysis, derived from peak fits of XPS data.

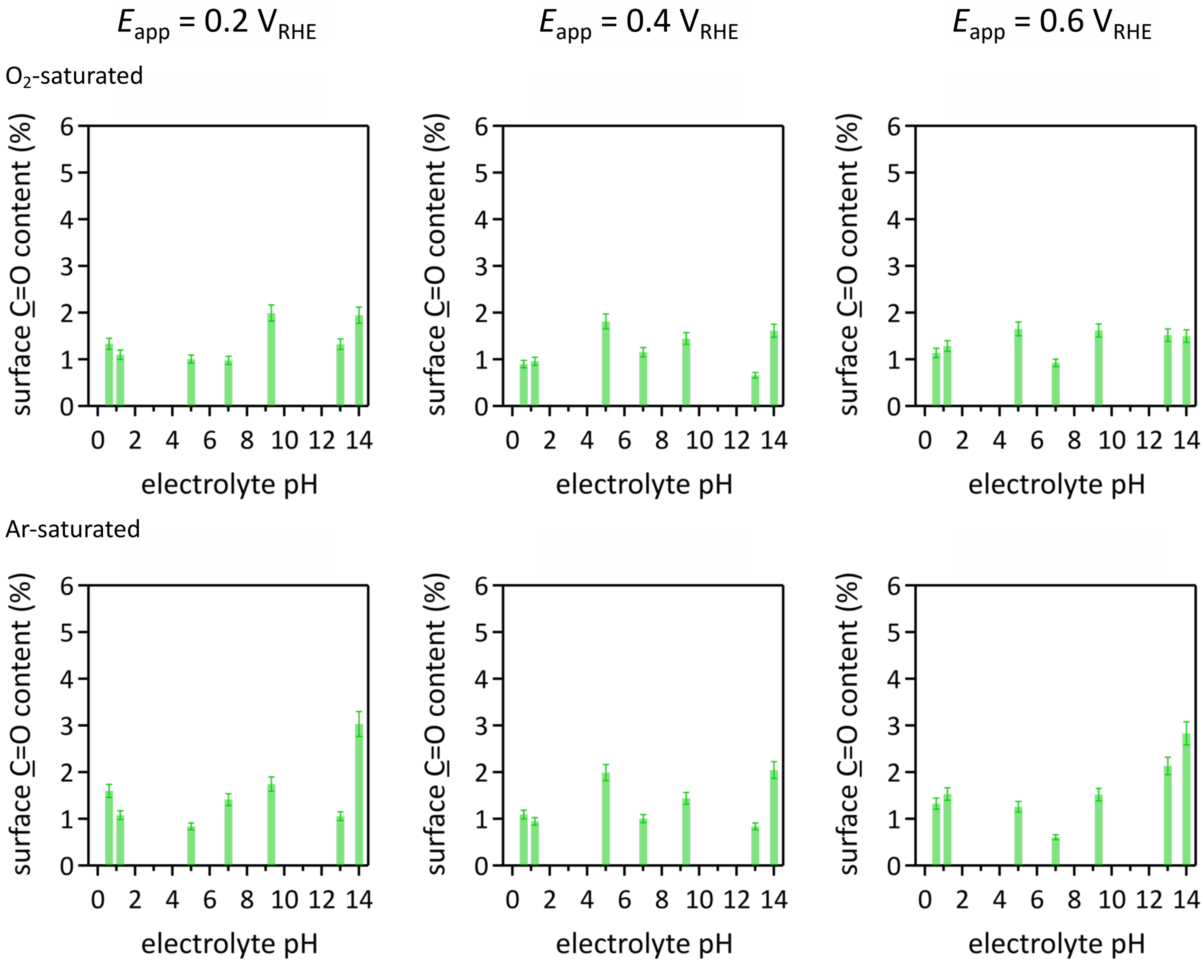


**Figure S31.** Surface C=O content on hydrophilic carbon fiber paper post catalysis, derived from peak fits of XPS data.

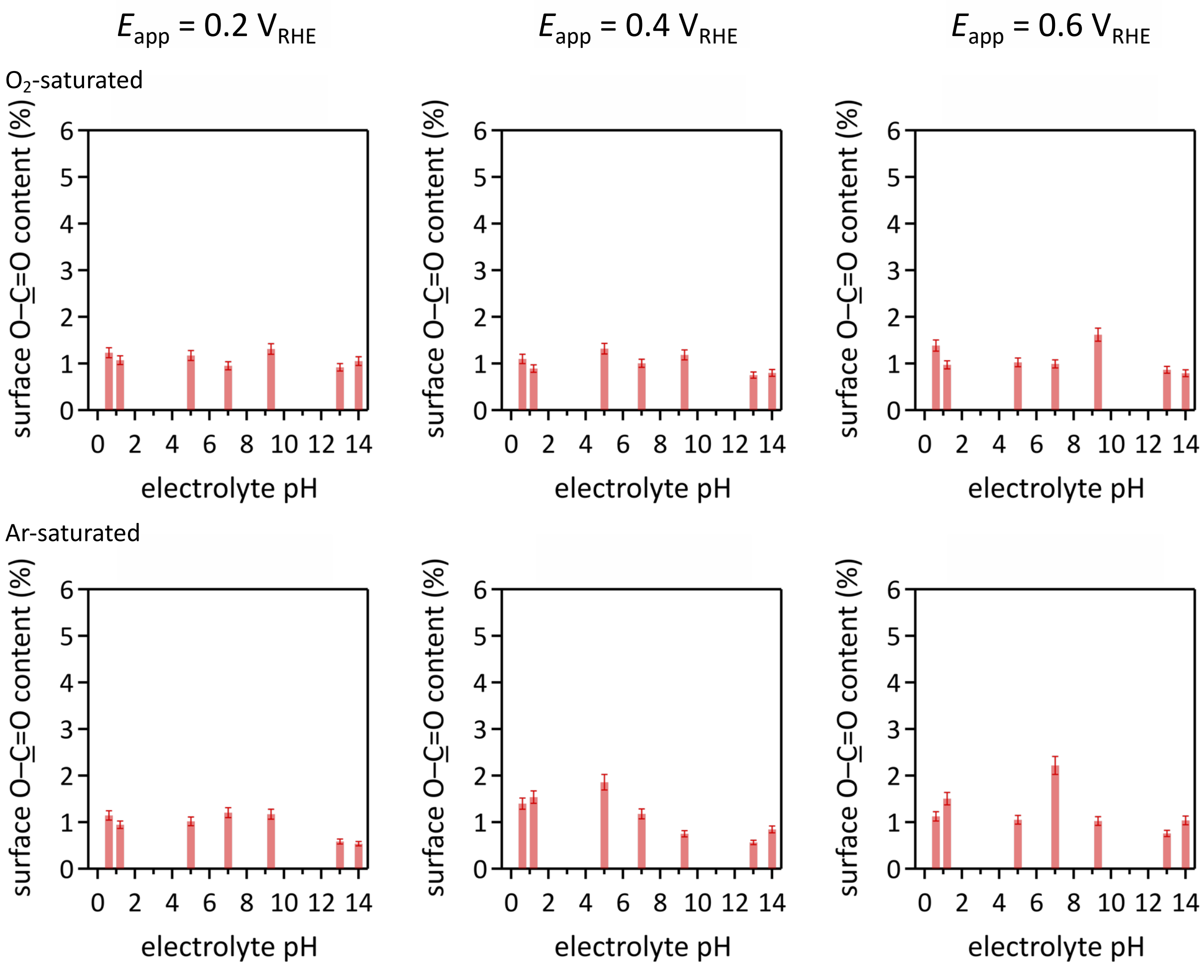


**Figure S32.** Surface O–C=O content on hydrophilic carbon fiber paper post catalysis, derived from peak fits of XPS data.

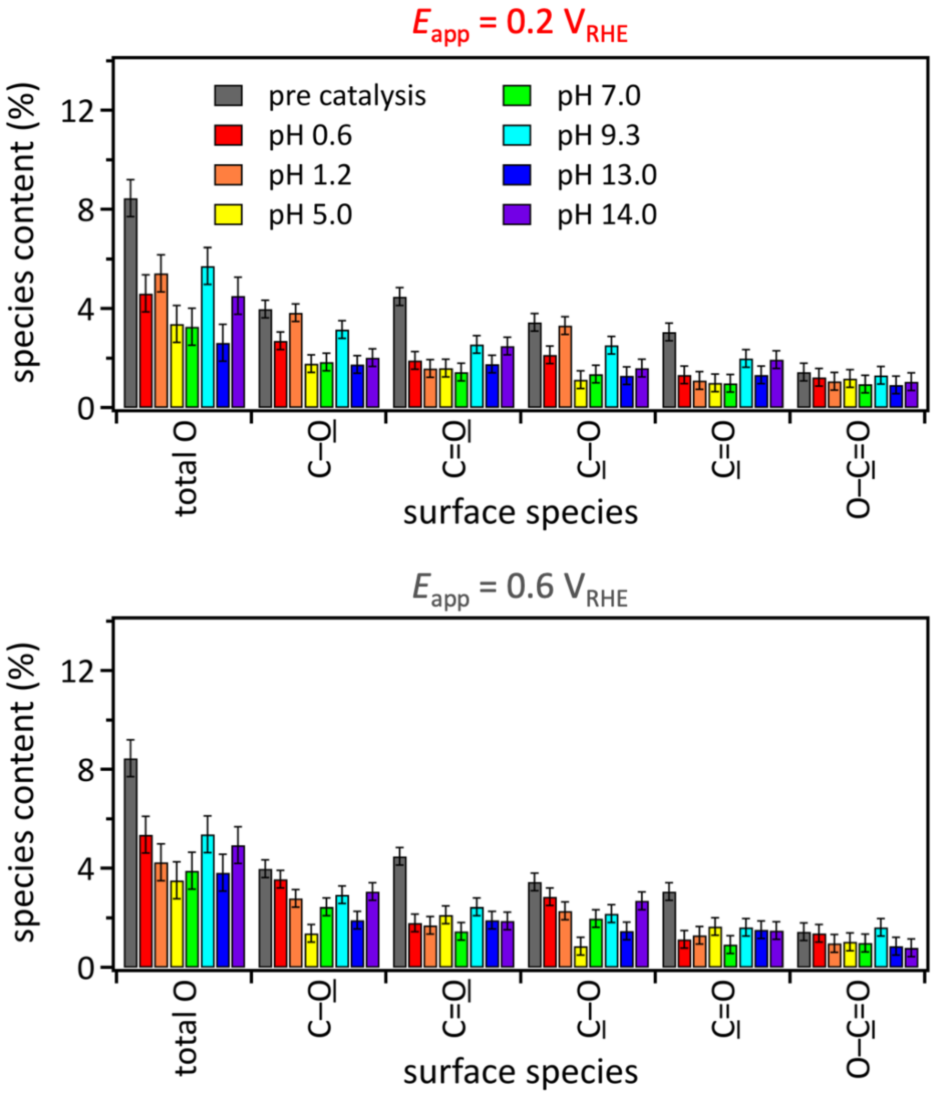


**Figure S33.** Carbon surface oxygenates. Surface contents derived from XPS data of total or individual oxygenated carbon species on hydrophilic carbon fiber paper pre catalysis and after 2 h of electrocatalysis in aqueous electrolytes with pH values ranging from 0.6 to 14.

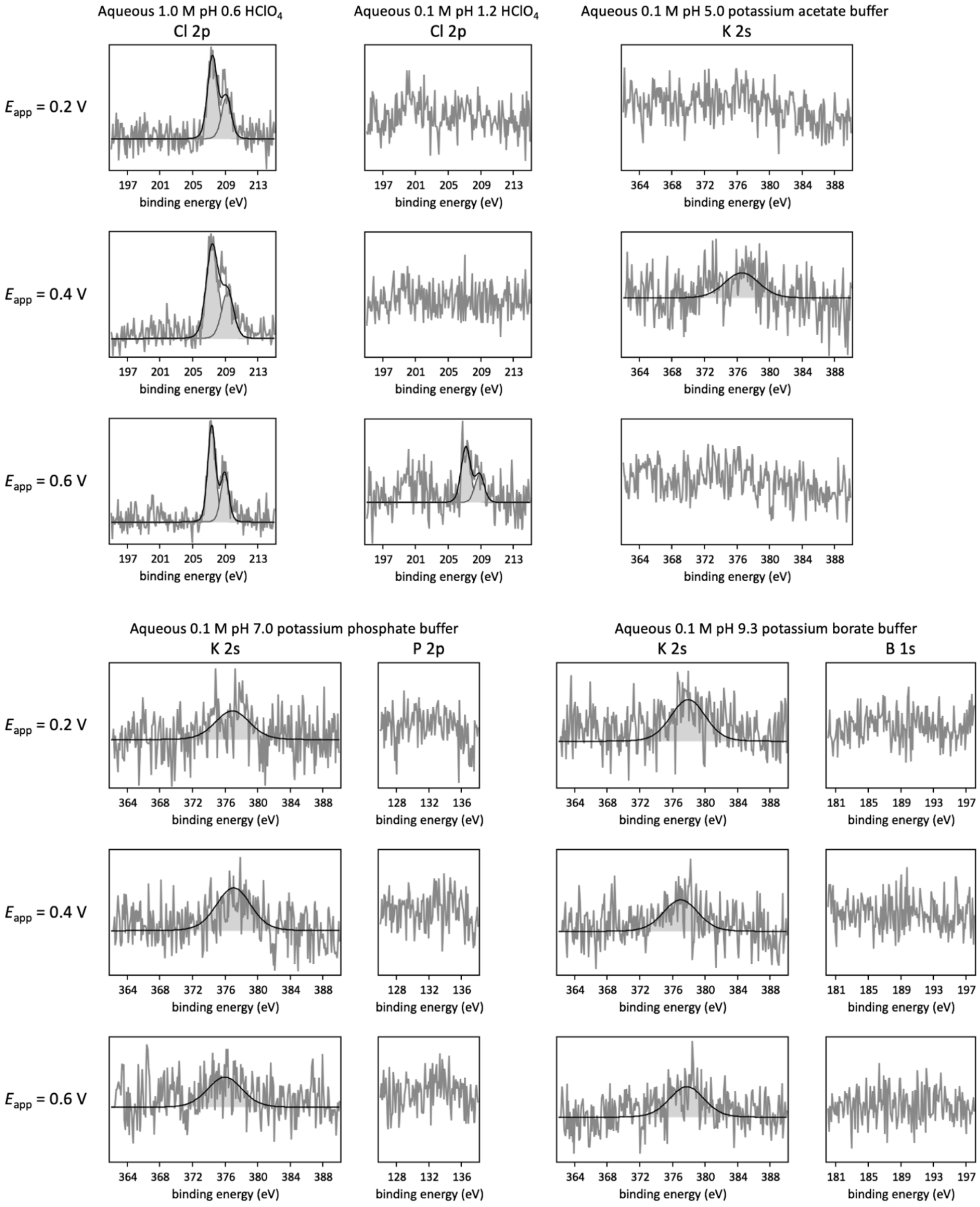


**Figure S34.** High resolution XPS data and peak fits of hydrophilic carbon fiber paper after 2 h of electrocatalysis in $O_2$-saturated aqueous electrolytes at 0.2, 0.4, or 0.6 V vs RHE.

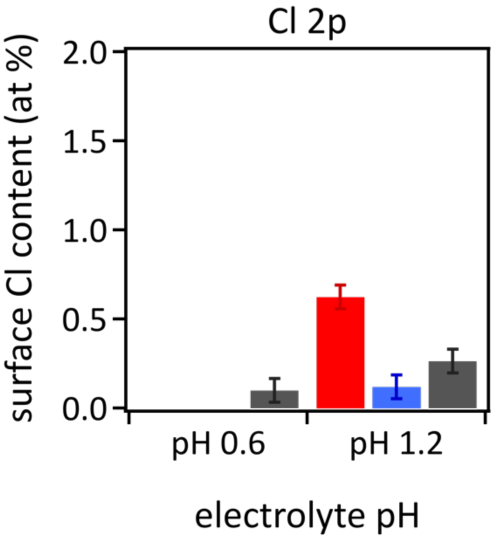


**Figure S35.** Surface chlorine content on hydrophilic carbon fiber paper post catalysis, derived from peak fits of XPS data. Colors: applied potential of 0.2 (red), 0.4 (blue), or 0.6 V vs RHE (gray).

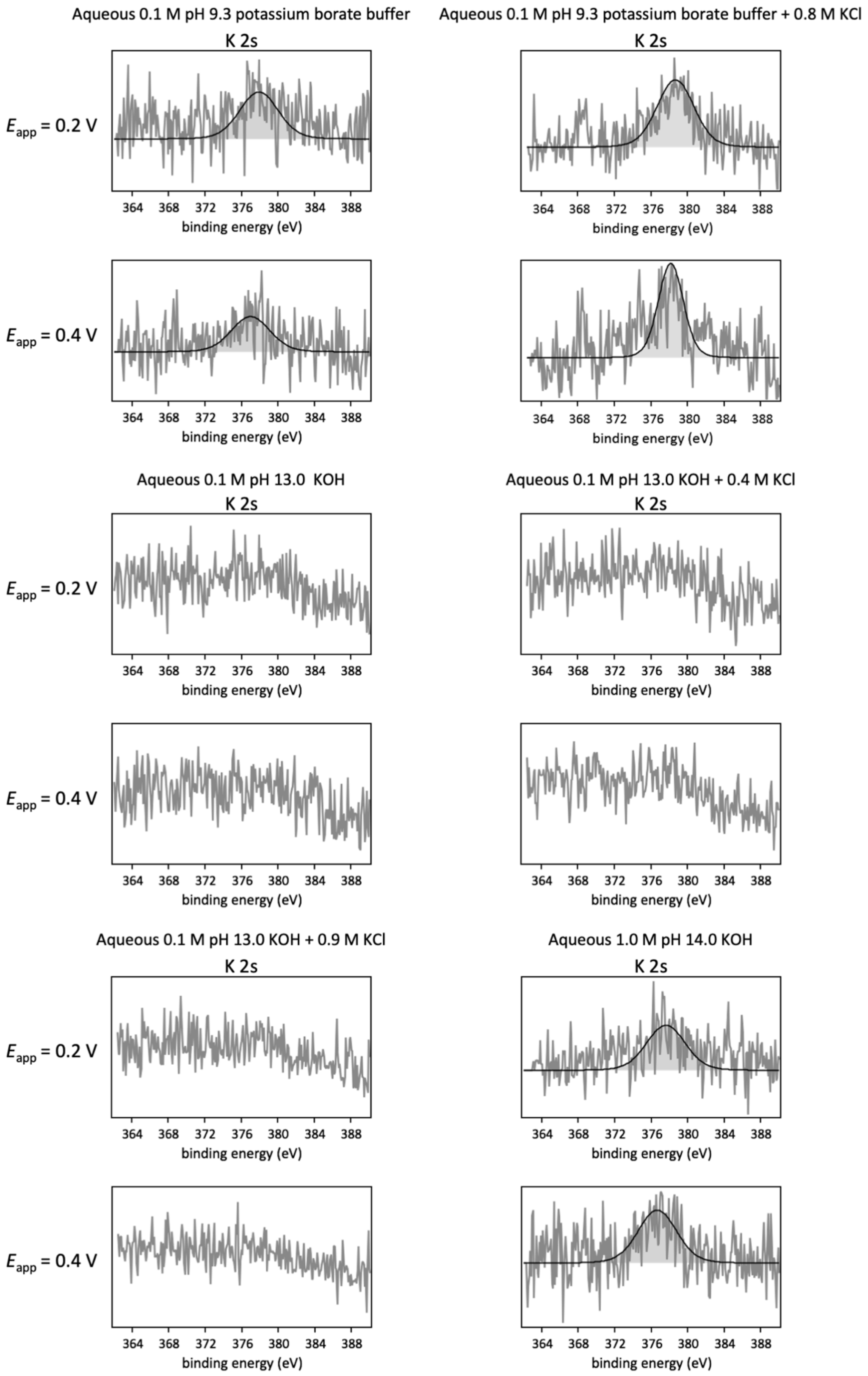


**Figure S36.** High-resolution K 2s XPS data and peak fits of hydrophilic carbon fiber paper after 2 h of electrocatalysis in $O_2$-saturated aqueous electrolytes at 0.2 or 0.4 V vs RHE.

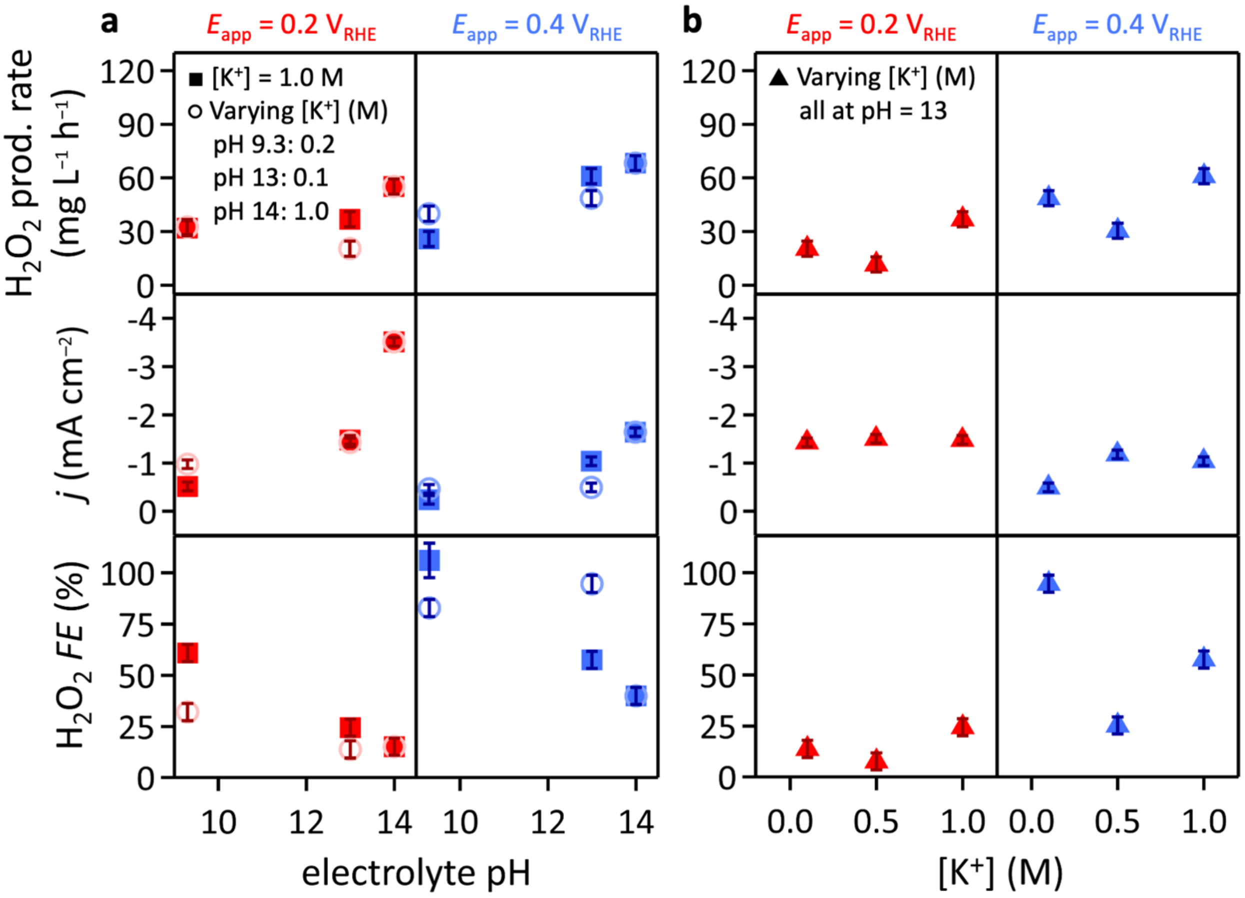


**Figure S37.** $H_2O_2$ production rate, generated current density, and $H_2O_2$ *FE* as a function of applied potential in (a) electrolytes of varying pH and constant $K^+$ concentration of 1.0 M (solid squares), or (b) electrolytes of varying $K^+$ concentration of 0.1 to 1.0 M and a constant pH of 13.0 (solid triangles). Also depicted are data from **Figure 2b** as open circles in panel (a).

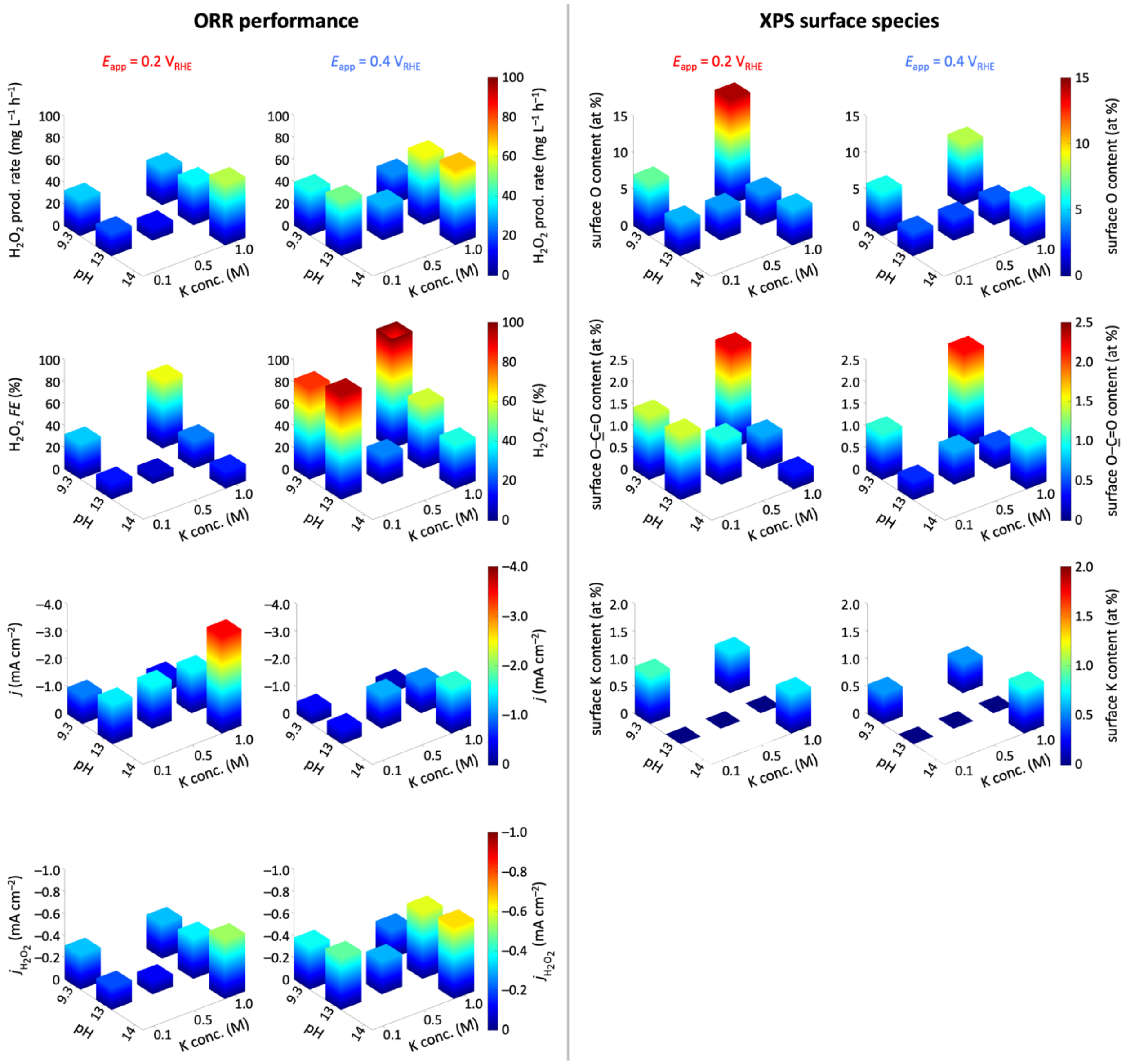


**Figure S38.** Bivariate representation of ORR performance metrics (left) and surface contents (right), at 0.2 and 0.4 V vs RHE in aqueous electrolytes with varying pH and $K^+$ concentration. XPS data and peak fits are shown in **Figure S36** and **Figure S39–S41**; $j_{H2O2}$, $H_2O_2$ partial current density.

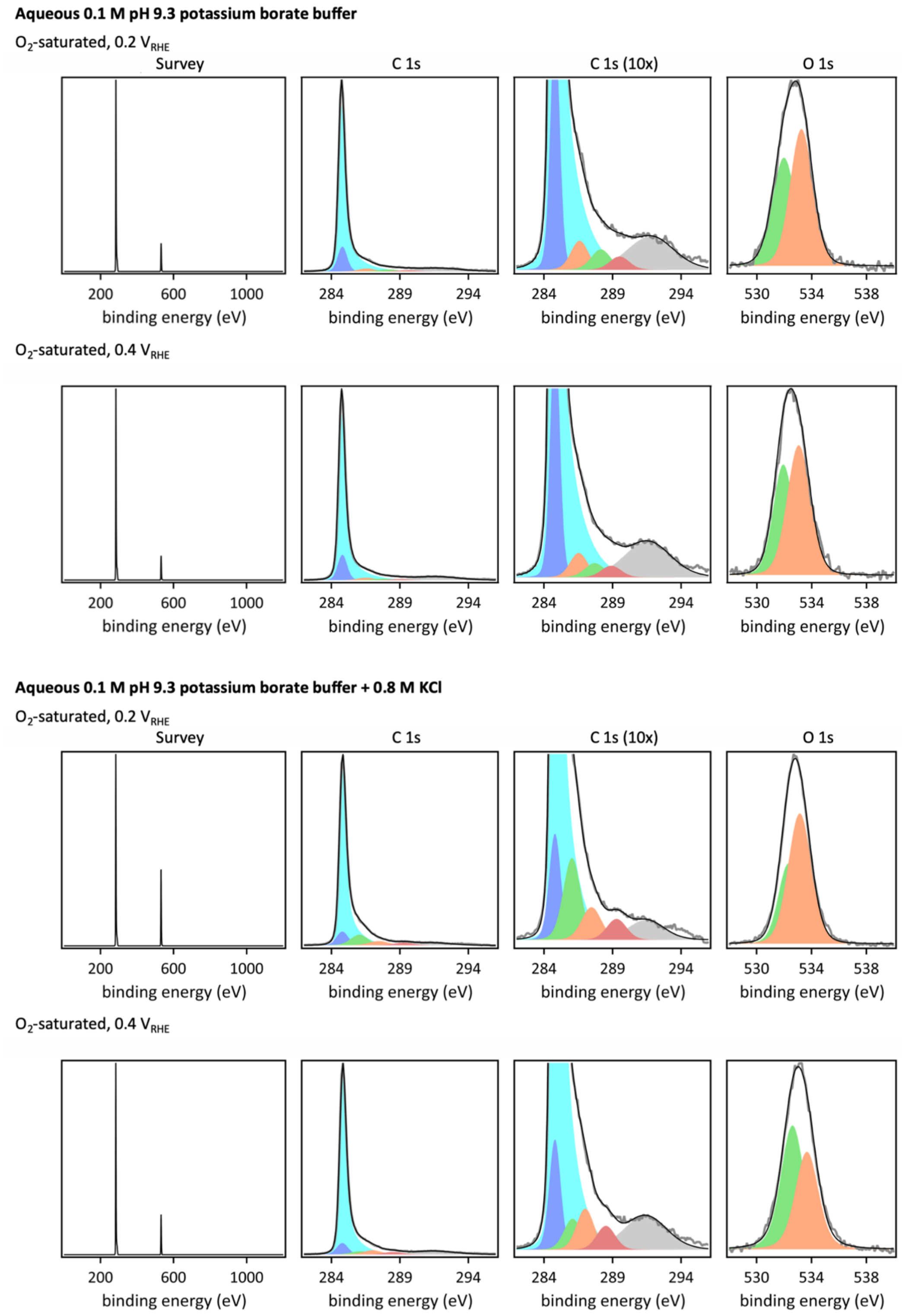


**Figure S39.** High-resolution C 1s and O 1s XPS data and peak fits of hydrophilic carbon fiber paper after 2 h of electrocatalysis in $O_2$-saturated aqueous electrolytes as specified at 0.2 or 0.4 V vs RHE; peaks: graphitic carbon (cyan), adventitious carbon (lavender), shake-up peak (gray), C–O (orange), C=O (green), O=C–O (red).

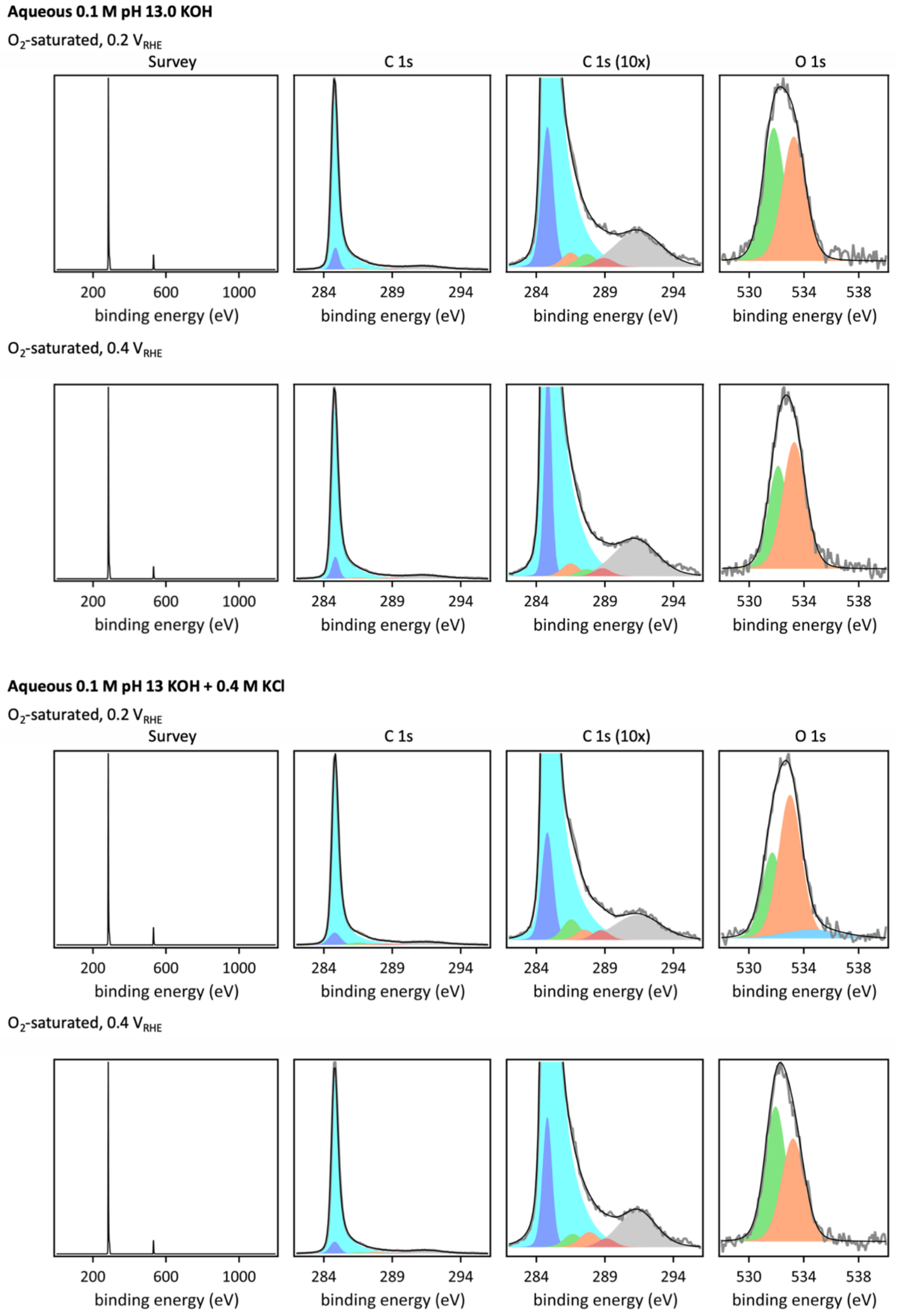


**Figure S40.** High-resolution C 1s and O 1s XPS data and peak fits of hydrophilic carbon fiber paper after 2 h of electrocatalysis in $O_2$-saturated aqueous electrolytes as specified at 0.2 or 0.4 V vs RHE; peaks: graphitic carbon (cyan), adventitious carbon (lavender), shake-up peak (gray), C–O (orange), C=O (green), O=C–O (red).

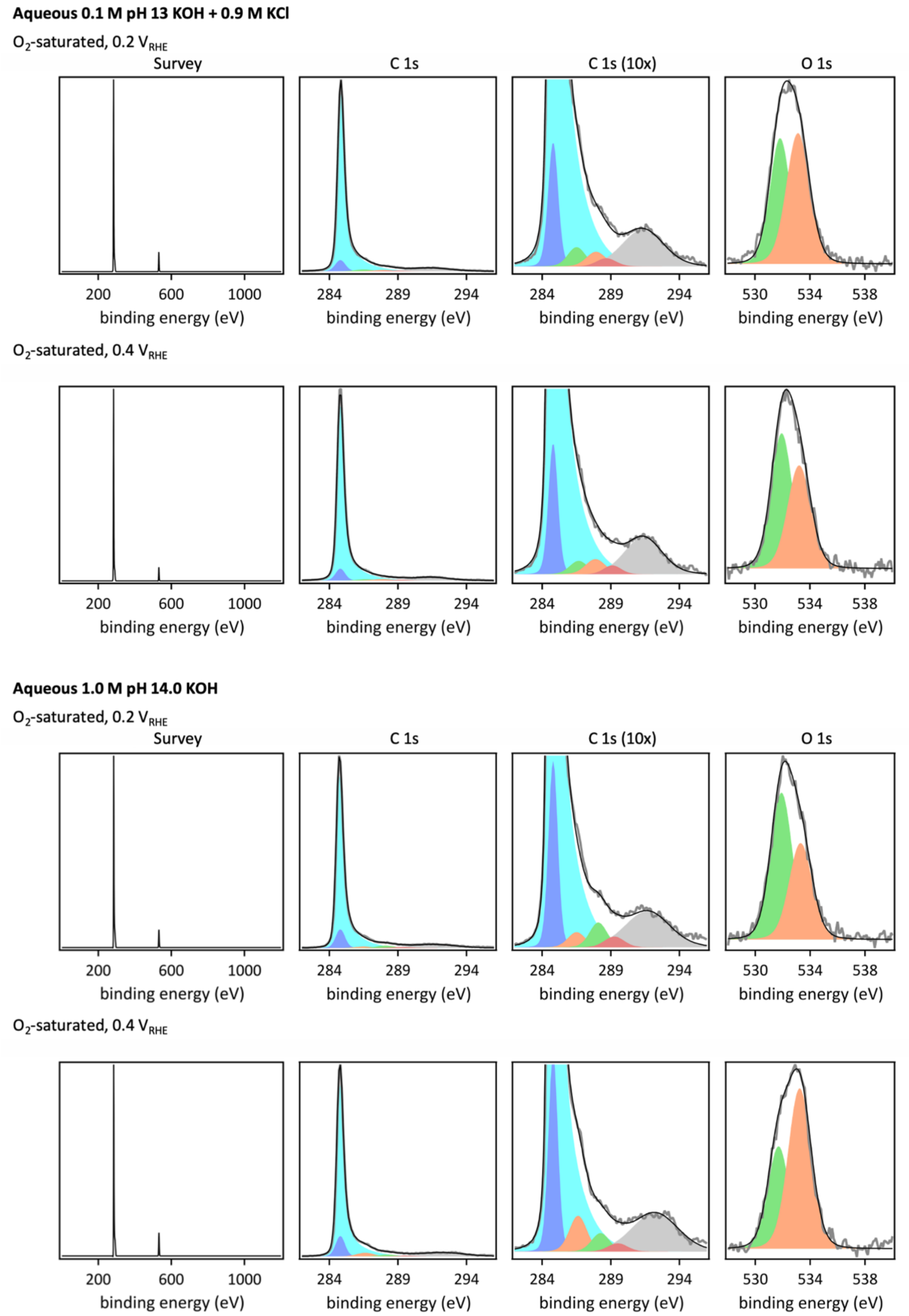


**Figure S41.** High-resolution C 1s and O 1s XPS data and peak fits of hydrophilic carbon fiber paper after 2 h of electrocatalysis in $O_2$-saturated aqueous electrolytes as specified at 0.2 or 0.4 V vs RHE; peaks: graphitic carbon (cyan), adventitious carbon (lavender), shake-up peak (gray), C–O (orange), C=O (green), O=C–O (red).

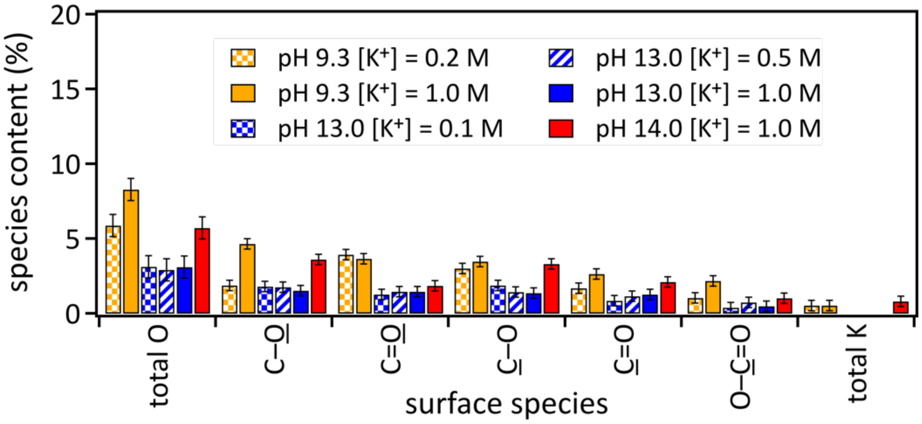


**Figure S42.** Surface carbon oxygenate and potassium contents, derived from XPS data of hydrophilic carbon fiber paper after 2 h of electrocatalysis at 0.4 V vs RHE in $O_2$-saturated aqueous electrolytes with pH values ranging from 9.3 to 14 and $K^+$ concentrations between 0.1 and 1.0 M. XPS data and peak fits are shown in **Figure S36** and **Figure S39–S41**.

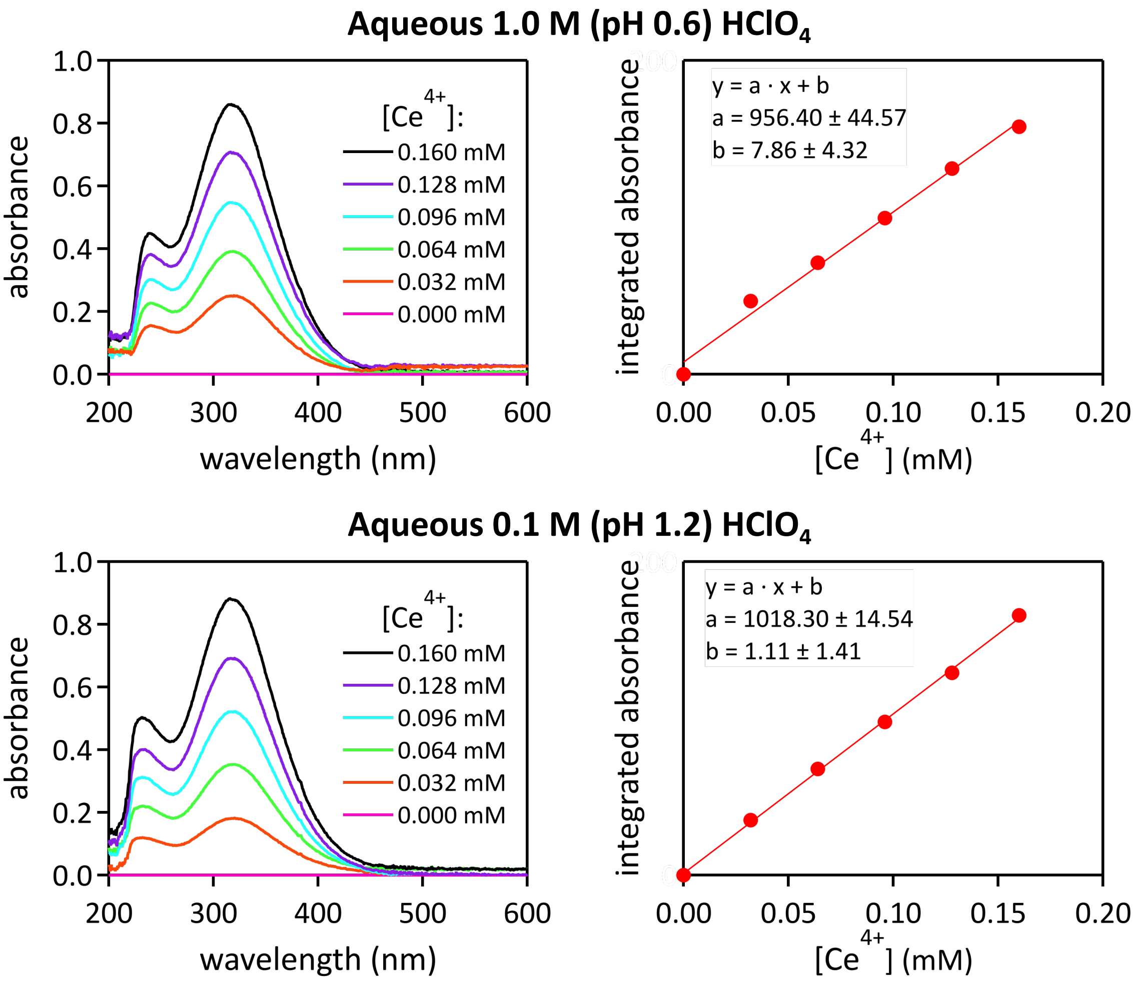


**Figure S43.** Optical spectra of solutions of $Ce(SO_4)_2$ with known concentrations in each electrolyte and associated calibration curves for electrolytes with pH values of 0.6 and 1.2.

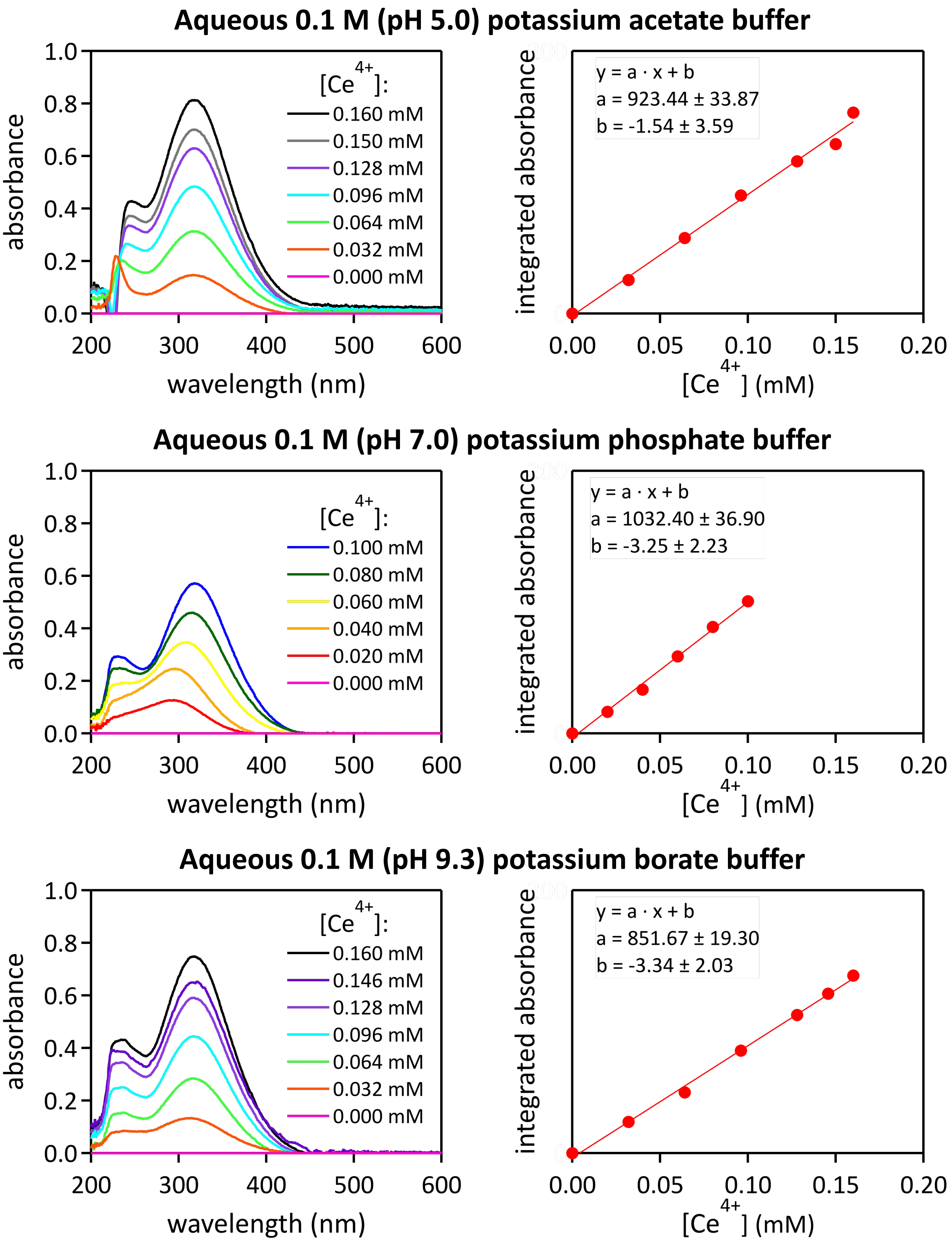


**Figure S44.** Optical spectra of solutions of $Ce(SO_4)_2$ with known concentrations in each electrolyte and associated calibration curves for electrolytes with pH values of 5.0 to 9.3.

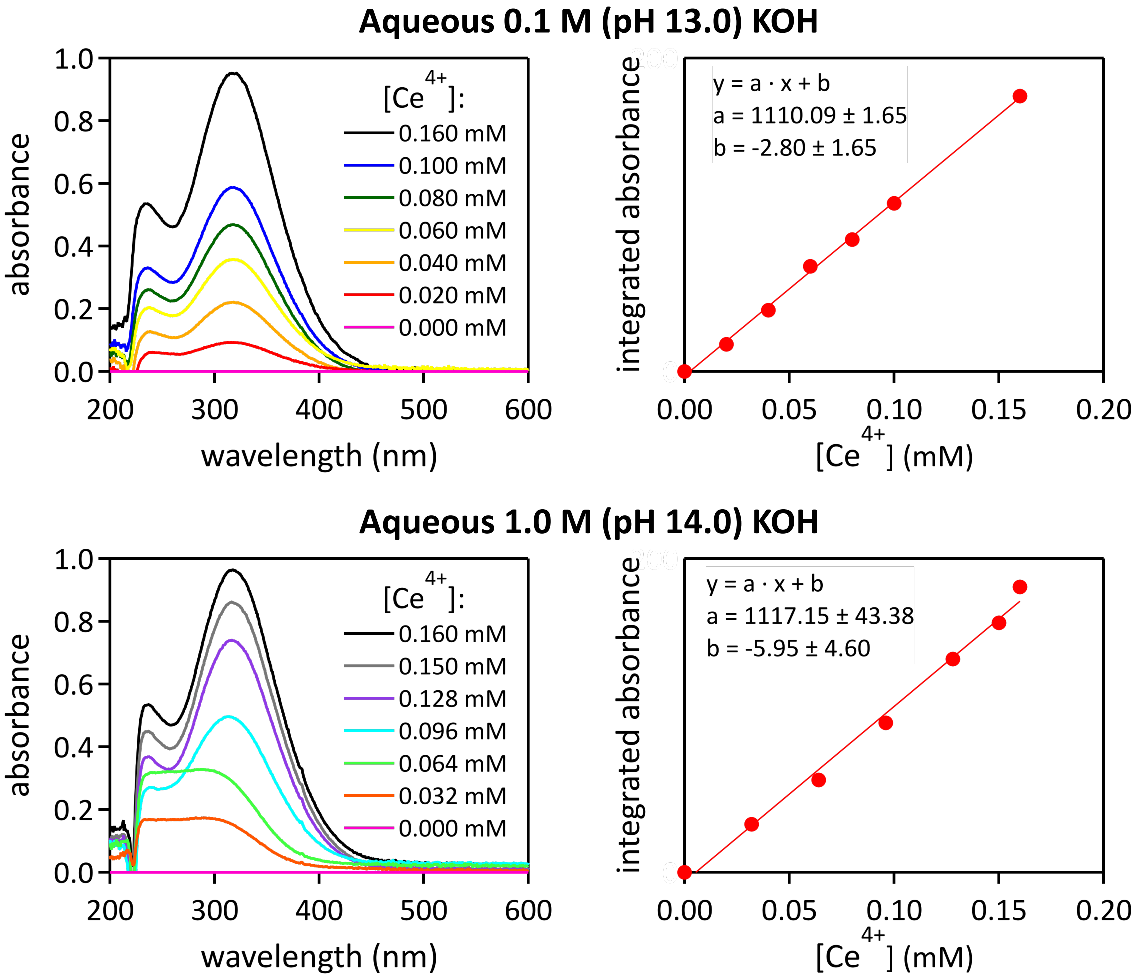


**Figure S45.** Optical spectra of solutions of $Ce(SO_4)_2$ with known concentrations in each electrolyte and associated calibration curves for electrolytes with pH values of 13.0 and 14.0.

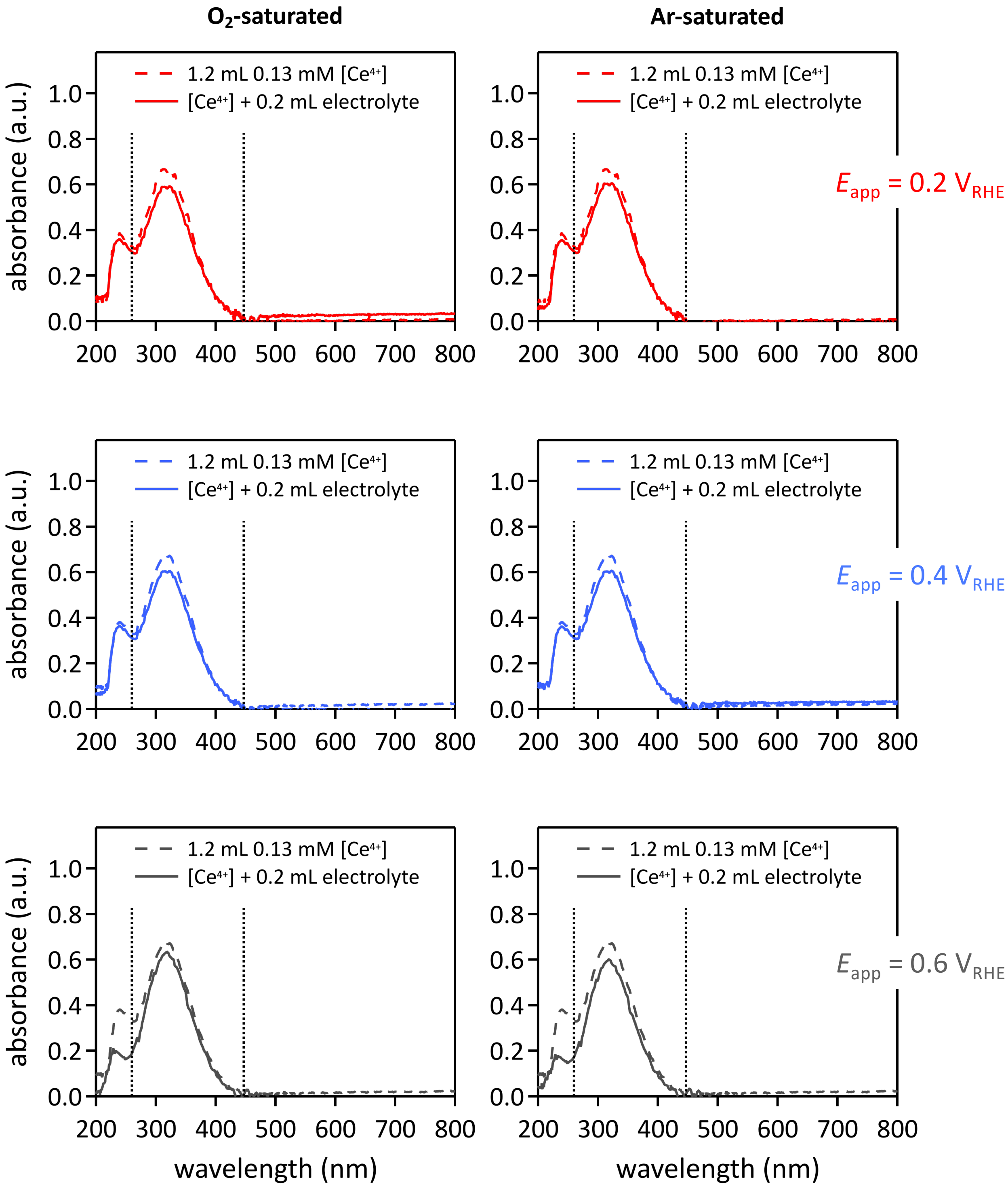


**Figure S46.** Optical spectra obtained for quantification of $H_2O_2$ production in pH 0.6 electrolyte. Spectra were integrated in the 260–447 nm range (between the dotted black lines) for improved accuracy over previously reported single-wavelength measurements.[6]

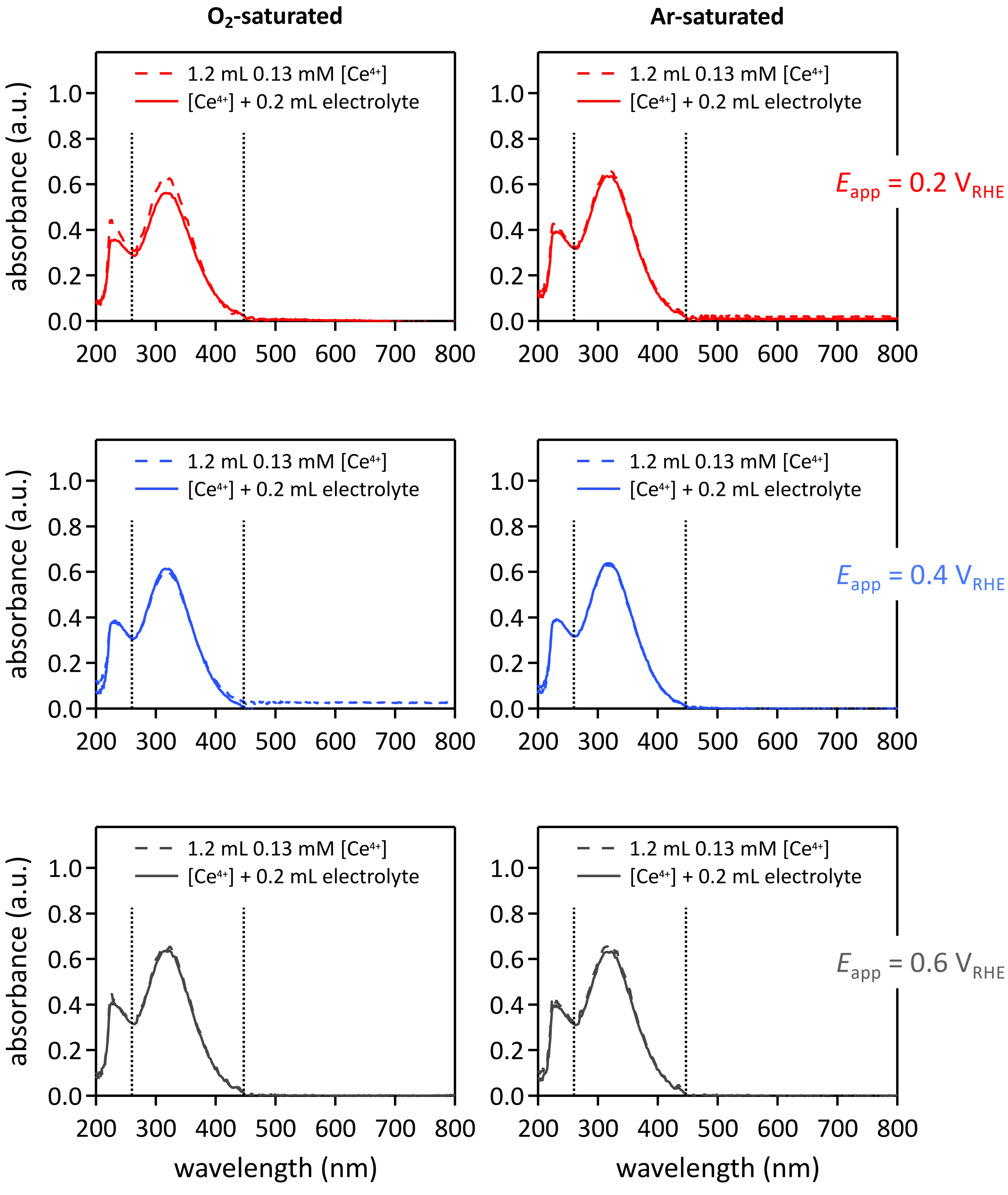


**Figure S47.** Optical spectra obtained for quantification of $H_2O_2$ production in pH 1.2 electrolyte. Spectra were integrated in the 260–447 nm range (between the dotted black lines) for improved accuracy over previously reported single-wavelength measurements.[6]

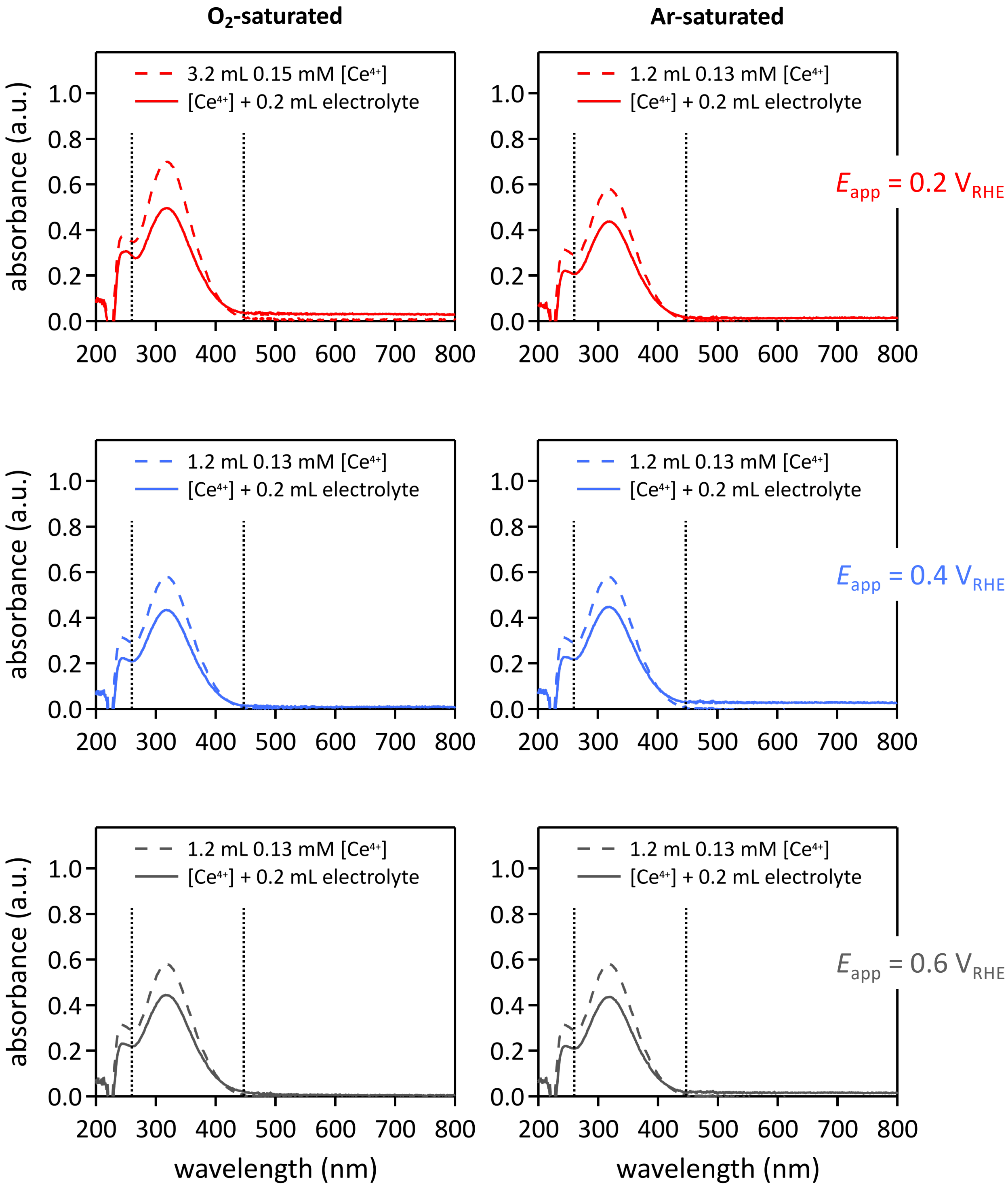


**Figure S48.** Optical spectra obtained for quantification of $H_2O_2$ production in pH 5.0 electrolyte. Spectra were integrated in the 260–447 nm range (between the dotted black lines) for improved accuracy over previously reported single-wavelength measurements.[6]

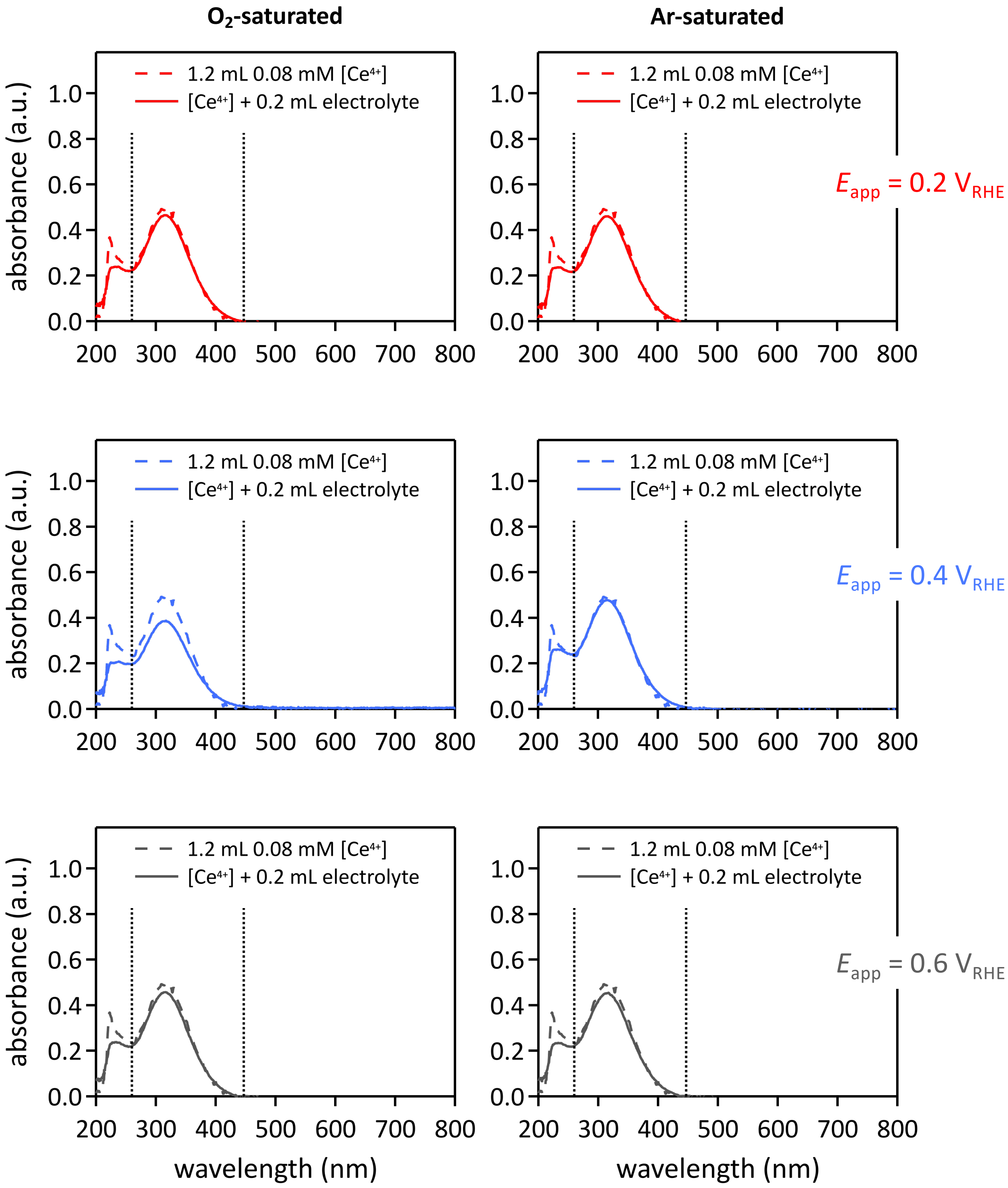


**Figure S49.** Optical spectra obtained for quantification of $H_2O_2$ production in pH 7.0 electrolyte. Spectra were integrated in the 260–447 nm range (between the dotted black lines) for improved accuracy over previously reported single-wavelength measurements.[6]

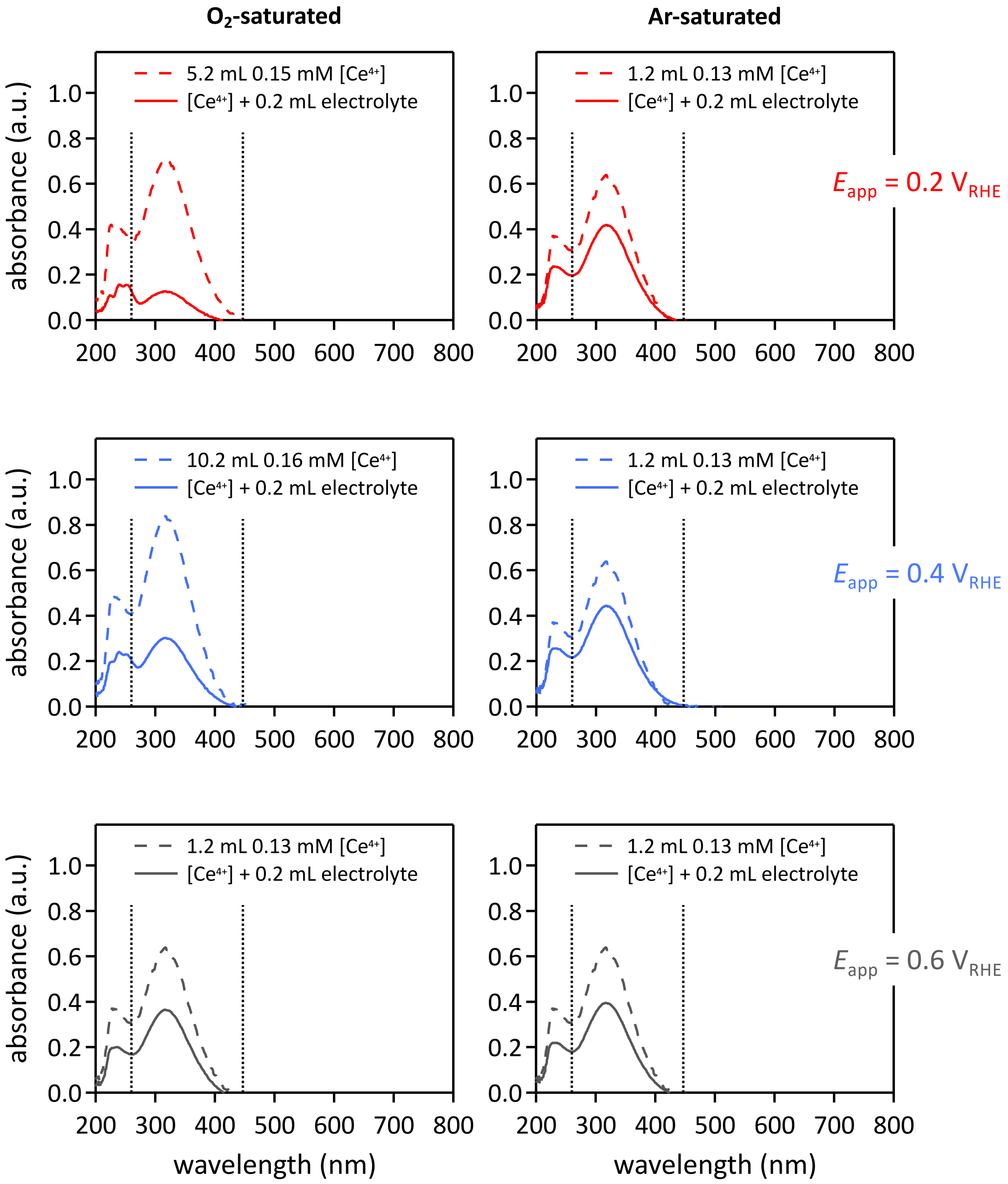


**Figure S50.** Optical spectra obtained for quantification of $H_2O_2$ production in pH 9.3 electrolyte. Spectra were integrated in the 260–447 nm range (between the dotted black lines) for improved accuracy over previously reported single-wavelength measurements.[6]

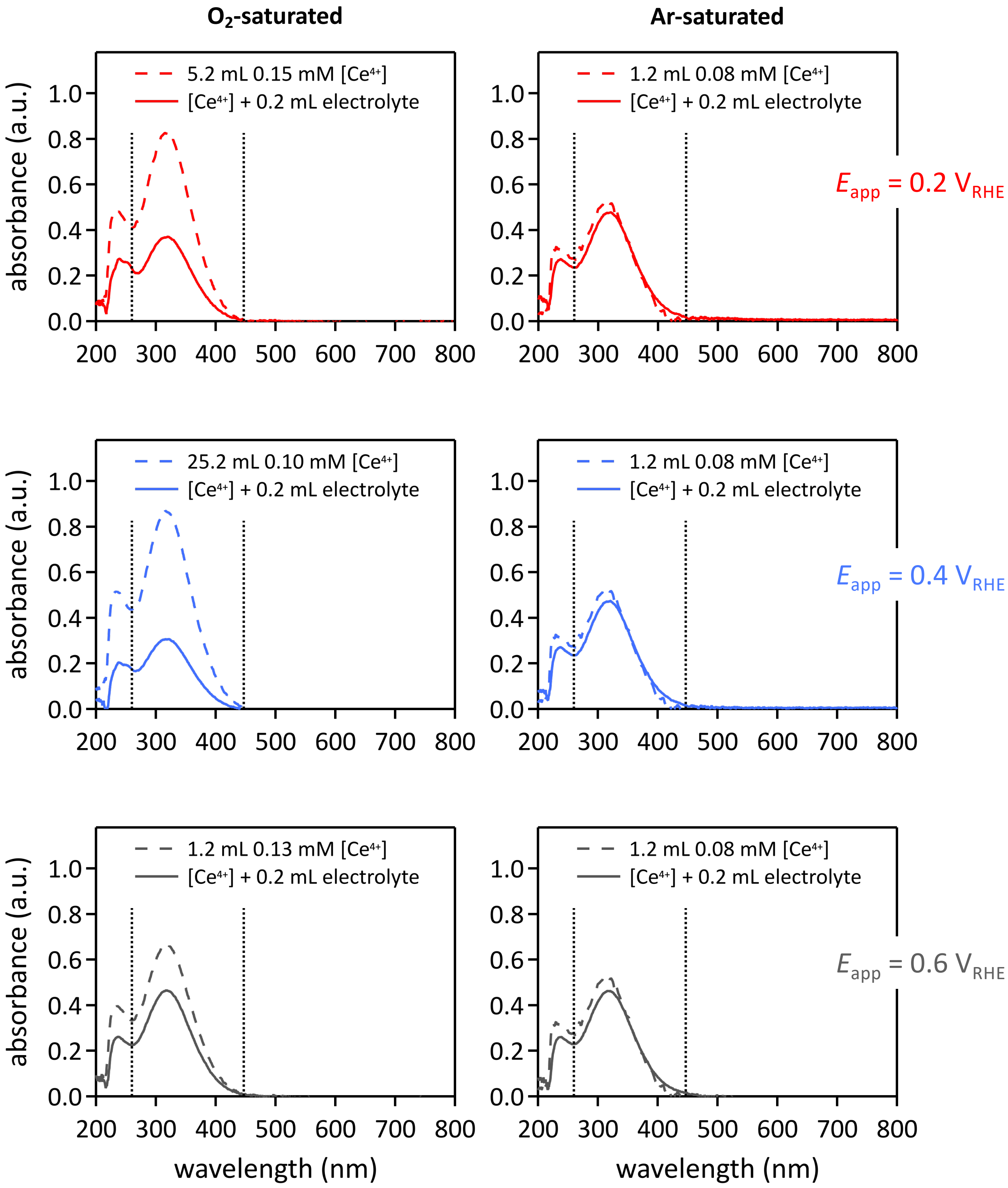


**Figure S51.** Optical spectra obtained for quantification of $H_2O_2$ production in pH 13.0 electrolyte. Spectra were integrated in the 260–447 nm range (between the dotted black lines) for improved accuracy over previously reported single-wavelength measurements.[6]

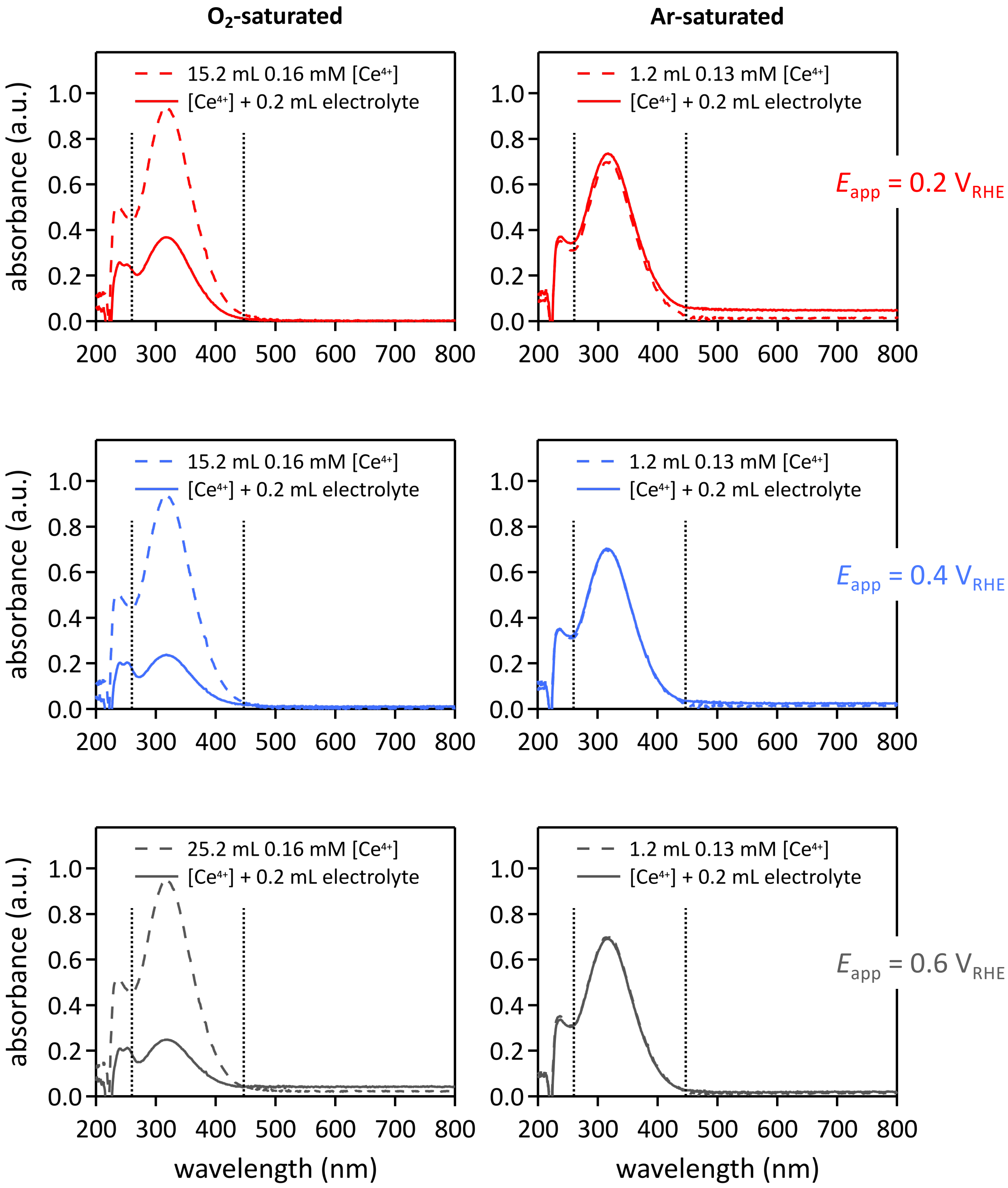


**Figure S52.** Optical spectra obtained for quantification of $H_2O_2$ production in pH 14.0 electrolyte. Spectra were integrated in the 260–447 nm range (between the dotted black lines) for improved accuracy over previously reported single-wavelength measurements.[6]

## REFERENCES


(1) Yang, S.; Verdaguer-Casadevall, A.; Arnarson, L.; Silvioli, L.; Čolić, V.; Frydendal, R.; Rossmeisl, J.; Chorkendorff, I.; Stephens, I. E. L. Toward the Decentralized Electrochemical Production of H2O2: A Focus on the Catalysis. *ACS Catal.* **2018**, *8*, 4064-4081.

(2) Yin, G.; Zhang, J., Rotating electrode methods and oxygen reduction electrocatalysts. Elsevier: 2014.

(3) Bard, A. J.; Faulkner, L. R., Electrochemical Methods: Fundamentals and Applications. 2nd ed.; Wiley: New York, NY, U.S.A., 1980.

(4) Han, G.-F.; Li, F.; Zou, W.; Karamad, M.; Jeon, J.-P.; Kim, S.-W.; Kim, S.-J.; Bu, Y.; Fu, Z.; Lu, Y. Building and identifying highly active oxygenated groups in carbon materials for oxygen reduction to H2O2. *Nat. Commun.* **2020**, *11*, 2209.

(5) Kim, H. W.; Ross, M. B.; Kornienko, N.; Zhang, L.; Guo, J.; Yang, P.; McCloskey, B. D. Efficient hydrogen peroxide generation using reduced graphene oxide-based oxygen reduction electrocatalysts. *Nat. Catal.* **2018**, *1*, 282-290.

(6) Lu, Z. Y.; Chen, G. X.; Siahrostami, S.; Chen, Z. H.; Liu, K.; Xie, J.; Liao, L.; Wu, T.; Lin, D. C.; Liu, Y. Y.; Jaramillo, T. F.; Norskov, J. K.; Cui, Y. High-efficiency oxygen reduction to hydrogen peroxide catalysed by oxidized carbon materials. *Nat. Catal.* **2018**, *1*, 156-162.

(7) Zhang, X.; Zhao, X.; Zhu, P.; Adler, Z.; Wu, Z.-Y.; Liu, Y.; Wang, H. Electrochemical oxygen reduction to hydrogen peroxide at practical rates in strong acidic media. *Nat. Commun.* **2022**, *13*, 2880.

(8) Dong, K.; Liang, J.; Wang, Y.; Xu, Z.; Liu, Q.; Luo, Y.; Li, T.; Li, L.; Shi, X.; Asiri, A. M. Honeycomb Carbon Nanofibers: A Superhydrophilic O2-Entrapping Electrocatalyst Enables Ultrahigh Mass Activity for the Two-Electron Oxygen Reduction Reaction. *Angew. Chem. Int. Ed.* **2021**, *60*, 10583-10587.

(9) Miao, J.; Zhu, H.; Tang, Y.; Chen, Y.; Wan, P. Graphite felt electrochemically modified in H2SO4 solution used as a cathode to produce H2O2 for pre-oxidation of drinking water. *Chem. Eng. J.* **2014**, *250*, 312-318.

(10) Vasconcelos, V. M.; Ponce-de-Leon, C.; Nava, J. L.; Lanza, M. R. d. V. Electrochemical degradation of RB-5 dye by anodic oxidation, electro-Fenton and by combining anodic oxidation–electro-Fenton in a filter-press flow cell. *J. Electroanal. Chem.* **2016**, *765*, 179-187.

(11) Wang, Z.; Li, Q.-K.; Zhang, C.; Cheng, Z.; Chen, W.; McHugh, E. A.; Carter, R. A.; Yakobson, B. I.; Tour, J. M. Hydrogen peroxide generation with 100% faradaic efficiency on metal-free carbon black. *ACS Catal.* **2021**, *11*, 2454-2459.

(12) Cornejo, O. M.; Sirés, I.; Nava, J. L. Cathodic generation of hydrogen peroxide sustained by electrolytic O2 in a rotating cylinder electrode (RCE) reactor. *Electrochim. Acta* **2022**, *404*, 139621.

(13) Karatas, O.; Gengec, N. A.; Gengec, E.; Khataee, A.; Kobya, M. High-performance carbon black electrode for oxygen reduction reaction and oxidation of atrazine by electro-Fenton process. *Chemosphere* **2022**, *287*, 132370.

(14) Li, C.; Hu, C.; Song, Y.; Sun, Y.-M.; Yang, W.; Ma, M. Graphene-based synthetic fabric cathodes with specific active oxygen functional groups for efficient hydrogen peroxide generation and homogeneous electro-Fenton processes. *Carbon* **2022**, *186*, 699-710.

(15) Mousset, E.; Wang, Z.; Hammaker, J.; Lefebvre, O. Physico-chemical properties of pristine graphene and its performance as electrode material for electro-Fenton treatment of wastewater. *Electrochim. Acta* **2016**, *214*, 217-230.

(16) Babaei-Sati, R.; Parsa, J. B. Electrogeneration of H2O2 using graphite cathode modified with electrochemically synthesized polypyrrole/MWCNT nanocomposite for electro-Fenton process. *J. Ind. Eng. Chem.* **2017**, *52*, 270-276.

(17) Ma, P.; Ma, H.; Galia, A.; Sabatino, S.; Scialdone, O. Reduction of oxygen to H2O2 at carbon felt cathode in undivided cells. Effect of the ratio between the anode and the cathode surfaces and of other operative parameters. *Sep. Purif. Technol.* **2019**, *208*, 116-122.

(18) Ergan, B. T.; Gengec, E. Dye degradation and kinetics of online Electro-Fenton system with thermally activated carbon fiber cathodes. *J. Env. Chem. Eng.* **2020**, *8*, 104217.

(19) Zhou, L.; Zhou, M.; Zhang, C.; Jiang, Y.; Bi, Z.; Yang, J. Electro-Fenton degradation of p-nitrophenol using the anodized graphite felts. *Chem. Eng. J.* **2013**, *233*, 185-192.

(20) Zhou, J.; An, X.; Lan, H.; Liu, H.; Qu, J. New insights into the surface-dependent activity of graphitic felts for the electro-generation of H2O2. *Appl. Surf. Sci.* **2020**, *509*, 144875.

(21) Xu, H.; Zhang, Z.; Guo, H.; Lin, X.; Li, N.; Xu, W. Electrogeneration of hydrogen peroxide by oxygen reduction using anodized graphite felt. *J. Taiwan Inst. Chem. Eng.* **2021**, *125*, 387-393.

(22) Guo, H.; Xu, H.; Zhao, C.; Hao, X.; Yang, Z.; Xu, W. High-effective generation of H2O2 by oxygen reduction utilizing organic acid anodized graphite felt as cathode. *J. Ind. Eng. Chem.* **2022**, *108*, 466-475.

(23) Xu, H.; Guo, H.; Chai, C.; Li, N.; Lin, X.; Xu, W. Anodized graphite felt as an efficient cathode for in-situ hydrogen peroxide production and Electro-Fenton degradation of rhodamine B. *Chemosphere* **2022**, *286*, 131936.

(24) Valim, R. B.; Reis, R. M.; Castro, P. S.; Lima, A. S.; Rocha, R. S.; Bertotti, M.; Lanza, M. R. Electrogeneration of hydrogen peroxide in gas diffusion electrodes modified with tert-butyl-anthraquinone on carbon black support. *Carbon* **2013**, *61*, 236-244.
